# Chemoenzymatic Asymmetric Synthesis of Complex Heterocycles: Dihydrobenzoxazinones and Dihydroquinoxalinones

**DOI:** 10.1021/acscatal.2c03008

**Published:** 2022-09-06

**Authors:** Mohammad Faizan Bhat, Alejandro Prats Luján, Mohammad Saifuddin, Gerrit J. Poelarends

**Affiliations:** Department of Chemical and Pharmaceutical Biology, Groningen Research Institute of Pharmacy, University of Groningen, Antonius Deusinglaan 1, 9713 AV Groningen, The Netherlands

**Keywords:** asymmetric synthesis, biocatalysis, dihydrobenzoxazinones, dihydroquinoxalinones, heterocycles

## Abstract

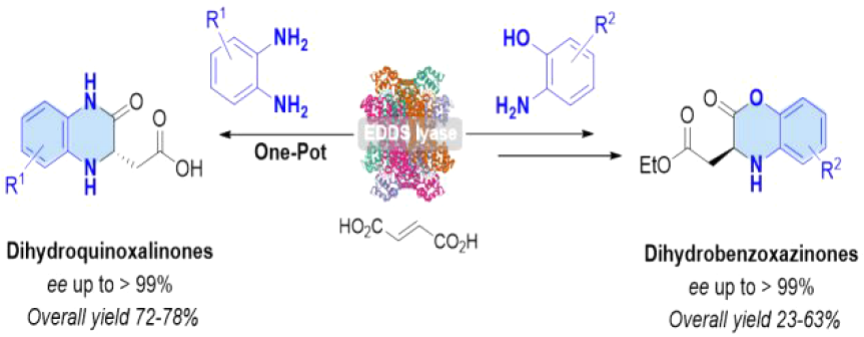

Chiral dihydrobenzoxazinones and dihydroquinoxalinones serve as essential building blocks for pharmaceuticals and agrochemicals. Here, we report short chemoenzymatic synthesis routes for the facile preparation of these complex heterocycles in an optically pure form. These synthetic routes involve a highly stereoselective hydroamination step catalyzed by ethylenediamine-*N*,*N′-*disuccinic acid lyase (EDDS lyase). This enzyme is capable of catalyzing the asymmetric addition of various substituted 2-aminophenols to fumarate to give a broad range of substituted *N-*(2-hydroxyphenyl)-l-aspartic acids with excellent enantiomeric excess (ee up to >99%). This biocatalytic hydroamination step was combined with an acid-catalyzed esterification–cyclization sequence to convert the enzymatically generated noncanonical amino acids into the desired dihydrobenzoxazinones in good overall yield (up to 63%) and high optical purity (ee up to >99%). By means of a similar one-pot, two-step chemoenzymatic approach, enantioenriched dihydroquinoxalinones (ee up to >99%) were prepared in good overall yield (up to 78%) using water as solvent for both steps. These chemoenzymatic methodologies offer attractive alternative routes to challenging dihydrobenzoxazinones and dihydroquinoxalinones, starting from simple and commercially available achiral building blocks.

Chiral dihydrobenzoxazinones (DHBs) and dihydroquinoxalinones (DHQs) are ubiquitous scaffolds that serve as important precursors for a broad range of pharmaceuticals, fungicides, and herbicides.^1a−1m^ For example, compounds **A**–**D** are important medicinal agents containing a DHB or DHQ pharmacophore with promising therapeutic efficacy (Figure 1). Compound **A**, a pyruvate kinase activator, can enhance the lifetime of red blood cells,^2^ while compound **B** finds use as a hypocholesterolemic agent.^3a,3b^ Compounds **C** and **D** contain a DHQ scaffold and find potential application in the treatment of leukemia^4^ and HIV-1, respectively (Figure 1).^1i,1j^ Conventional chemical strategies for the preparation of chiral DHBs involve the synthesis from optically pure amino acid precursors (Figure 2a),^5^*in situ* generation of ketenes followed by a highly stereoselective [4 + 2] cycloaddition with *o-*benzoquinone imides (Figure 2b),^6a,6b^ and catalytic asymmetric hydrogenation (Figure 2c).^7a−7i^ Optically enriched DHQs are synthesized via coupling of chiral amino acids (or the corresponding esters) with *o-*nitroaryl bromides/iodides or *o-*nitroaryl fluorides in the presence of catalytic Cu(I)^8^ or base,^9a−9l^ respectively, followed by a reduction–cyclization sequence (Figure 2d), asymmetric reduction starting from corresponding imine substrates (Figure 2e),^10a−10f^ and, finally, a difficult solid-phase synthesis employing numerous steps starting from *o*-nitro-benzenesulfonyl chloride (Figure 2f).^10a^ With current synthesis routes often suffering from limitations such as the use of chiral starting materials, heavy metals, multiple steps, and harsh reaction conditions, there is a necessity to investigate alternate asymmetric synthesis methods that are possibly greener, more sustainable, and more step-economic.

**Figure 1:**
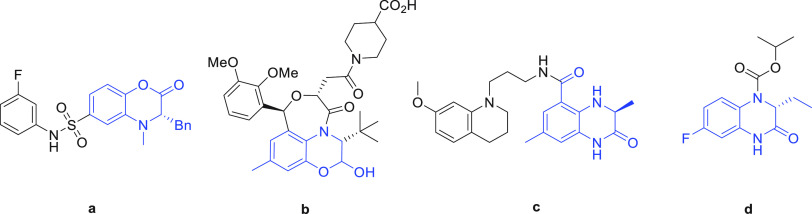
Bioactive molecules containing a chiral dihydrobenzoxazinone (a, pyruvate kinase activator; b, hypocholesterolemic agent) or dihydroquinoxalinone (c, leukemia agent; d, HIV-1 agent) scaffold.

**Figure 2:**
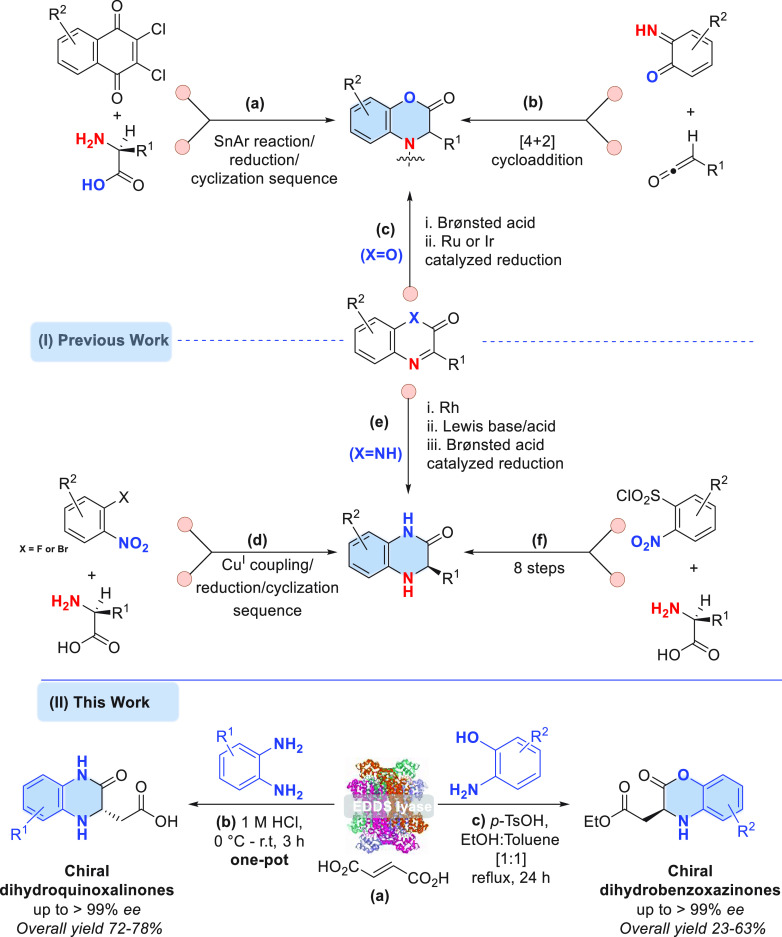
Methods toward the synthesis of chiral dihydroquinoxalinones and chiral dihydrobenzoxazinones. (Ia) SnAr reaction–reduction–cyclization sequence. (Ib) [4 + 2] cycloaddition. (Ic) Brønsted-acid- or Ru/Ir-catalyzed reduction. (Id) Cu^I^ coupling–reduction–cyclization sequence. (Ie) Rh or Lewis base/acid or Brønsted-acid-catalyzed reduction. (If) 8-step synthesis protocol. (IIa) EDDS-lyase-catalyzed stereoselective synthesis of substituted aspartic acids using fumarate and 2-aminophenols or *o*-phenylenediamines as substrates. (IIb) HCl assisted ring closure of the intermediate amino acid products into the desired DHQs. (IIc) *p*-TsOH assisted esterification and ring closure of the intermediate amino acid products into the desired DHBs.

Here, we report chemoenzymatic methodologies for the asymmetric synthesis of DHBs and DHQs from retrosynthetically designed substrates. These approaches highlight a highly enantioselective carbon–nitrogen bond-forming step catalyzed by ethylenediamine*-N*,*N′-*disuccinic acid (EDDS) lyase and provide alternative synthetic choices for the preparation of difficult DHB and DHQ products.

EDDS lyase from *Chelativorans* sp. BNC1 promotes the reversible deamination of (*S*,*S*)-EDDS to give ethylene diamine and two molecules of fumarate.^11^ We have previously demonstrated that this enzyme accepts a broad range of amines, ranging from linear and cyclic aliphatic amines to aromatic amines and hydrazines, in the stereoselective hydroamination of fumaric acid, leading to the corresponding N-substituted aspartic acids.^12a−12c^ Inspired by the extensive substrate scope of EDDS lyase, we envisaged that 2-aminophenol and *o*-phenylenediamine could potentially be used as non-native amine substrates in the EDDS-lyase-catalyzed asymmetric hydroamination reaction to give the corresponding amino acid products, which can then possibly be cyclized to obtain the desired DHB and DHQ heterocycles (Figure 2).

We started our investigations by testing whether EDDS lyase can accept 2-aminophenol (**1a**, Tabel S1) as an unnatural substrate in the hydroamination of fumarate. Interestingly, EDDS lyase accepted **1a** as a substrate, giving the resultant N-substituted aspartic acid product **3a** (Table 1) with outstanding conversion (92%) and in respectable yield (73%). Pleasingly, the enzyme also accepted a variety of substituted 2-aminophenols (**1b**–**1i**, Table S1) in the hydroamination reaction, yielding the desired amino acids **3b**–**3i** (Table 1) with good conversion (66–86%) and in moderate to good isolated yield (49–76%). EDDS-lyase did not process the 2-aminophenols **1j**–**1o** (Table S1).

**Table 1:**
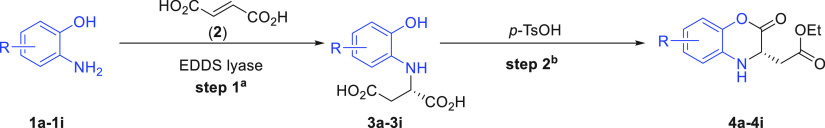

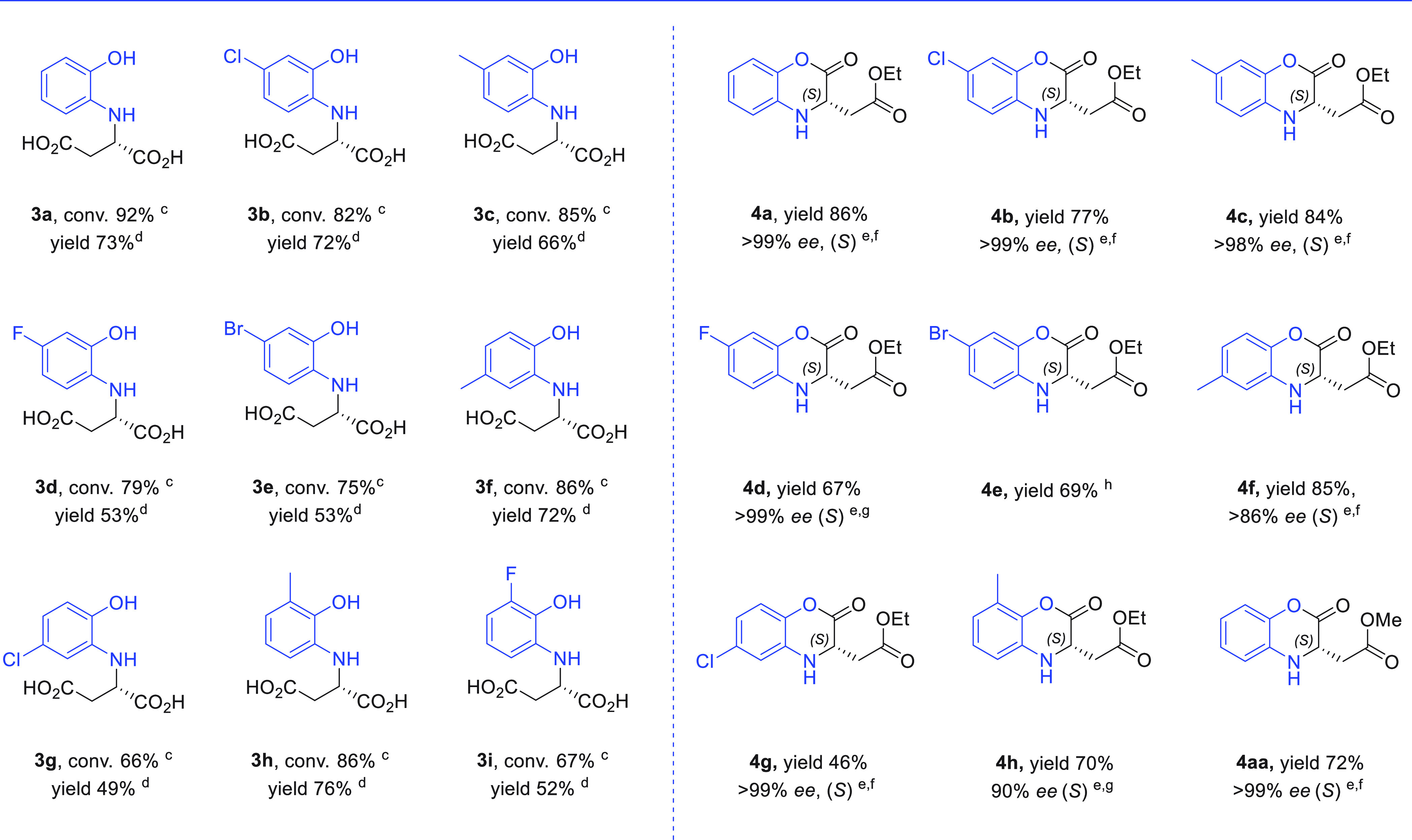
Chemoenzymatic Synthesis of DHBs

a The reaction mixture (40 mL) consisted of fumaric acid (**2**, 100 mM), 2-aminophenol substrate (**1a**–**1i**, 25 mM, except **1g** = 10 mM), and EDDS lyase (0.05 mol % based on 2-aminophenol) in 50 mM NaH_2_PO_4_/NaOH (pH 8.5, argon flushed), with DMSO (5%) as cosolvent at room temperature. A 5-fold excess of **2** (instead of an excess of amine) was used, facilitating product purification and avoiding enzyme inhibition as a result of high phenol substrate concentration.

b Stoichiometric amount of *p*-TsOH in toluene/EtOH [1:1, MeOH for **4aa**], reflux (24 h) under a nitrogen atmosphere (after 16 h, ethanol was removed, and reaction mixture refluxed in anhydrous toluene for additional 8 h).

c Conversions were measured by comparing ^1^H NMR signals of substrates and matching products.

d Isolated yield following cation-exchange chromatography.

e The enantiomeric excess (ee) was established by chiral HPLC using chemically prepared racemic standards.

f The absolute configurations were assigned as *S* by comparing the elution pattern of chemically prepared racemic standards and corresponding enzymatic products against previously reported chiral HPLC data.

g The absolute configuration was tentatively assigned as *S* based on analogy and in line with chiral HPLC data.

h Chiral HPLC separation could not be achieved. Cyclization could not be achieved for **3i**.

Although the biocatalytic preparation of the N-substituted aspartic acids **3a**–**3i** already shortens the synthesis of such medicinally important synthons by several steps,^13a^ we aimed to explore these compounds as precursors for the synthesis of more complex and pharmaceutically relevant chiral DHBs.^1a−1f^ Toward this end, we first tried to optimize the conditions for acid-catalyzed cyclization in water to give the corresponding enantiopure DHB from precursor **3a**. However, all the acidic conditions we tested (HCl, H_2_SO_4_, TFA, etc.) with varying temperatures (0–100 °C) gave either uncyclized starting material or multiple unidentified side products. To aid cyclization and purification, we then esterified amino acid **3a** using standard esterification conditions (SOCl_2_, cat. HCl in MeOH/EtOH) and obtained the corresponding ester product in quantitative yield. However, subsequent cyclization in the same solvent did not result in the final cyclized DHB. Therefore, we dissolved the ester product in a high-boiling solvent (toluene) to assist ring closure and obtained the final product **4a** in good isolated yield (81%) in the presence of stoichiometric amounts of *p*-TsOH. We then reasoned that if we use *p*-TsOH in the first esterification step in a toluene/ethanol mixture [1:1], we could get to the final product in a single esterification–cyclization step. Although the starting material was consumed after 18 h of refluxing conditions, we observed that the isolated compound was always a diester product, which is likely because of transesterification of the unstable cyclic **4a** in the presence of excess ethanol. Based on this data, after 16 h of reflux, ethanol was removed *in vacuo* and then the reaction mixture reheated in dry toluene until we reached full conversion to the desired DHB product **4a**, which was obtained in good isolated yield (86%).

Next, the optimized conditions for DHB formation were successfully used for the esterification–cyclization of the isolated amino acid intermediates **3a**–**3h** to produce the desired heterocycles **4a**–**4h** in moderate to good isolated yield (46–86%). Unfortunately, using the same conditions, we could not achieve the conversion of **3i** into **4i**. Analysis of the chemoenzymatically produced DHBs **4a**–**4h** by chiral HPLC, using chemically prepared racemic standards (see the Supporting Information), demonstrated that these heterocycles have excellent enantiopurity (up to >99% ee), possessing the *S* configuration, which is fully consistent with the well-characterized enantioselectivity of EDDS lyase.^13b^ As such, we have established a straightforward two-step chemoenzymatic route for the asymmetric preparation of enantioenriched DHBs in good overall yield (23–63%) and with high enantiopurity (up to >99% ee). Furthermore, the amino acid precursors **3a**–**3i**, which are synthesized in one enzymatic step, can be used as chiral synthons for pharmaceutically active compounds.^13c,13d^

Having established the two-step chemoenzymatic synthesis of enantiopure DHBs, we envisioned that a similar synthetic strategy could be used to produce biologically active DHQs.^1g,1i,4,10f,14^ To provide proof-of-concept for this strategy, we tested diamines **1p** and **1q** (Table S1) as non-native substrates for EDDS lyase. To our delight, EDDS lyase accepted these substrates in the hydroamination of fumarate to give the desired N-substituted aspartic acid products with high conversions. Next, we investigated if we could perform the intramolecular cyclization in one-pot to give the corresponding DHQ without isolating the intermediate amino acid. Toward this end, after completion of the enzymatic reaction (48 h), the reaction mixture was adjusted to 1 M hydrochloric acid with fuming HCl, giving the desired DHQ product (**5p** or **5q**, Figure 3) at room temperature in 3 h with good isolated overall yield (78% and 72%, respectively). Chiral HPLC examination, using chemically prepared reference compounds (see the Supporting Information), demonstrated that these products are highly enantioenriched (up to >99% ee), having the *S* configuration. Notably, EDDS lyase is able to accept a range of substituted aromatic diamines (**1r**–**1y**, Table S1) in the hydroamination of fumarate yielding the corresponding aspartic acid derivatives, potentially enabling the chemoenzymatic preparation of diverse DHQ synthons. The diamines **1z**–**1zd** (Table S1) were not accepted as alternative substrates by the enzyme.

**Figure 3:**
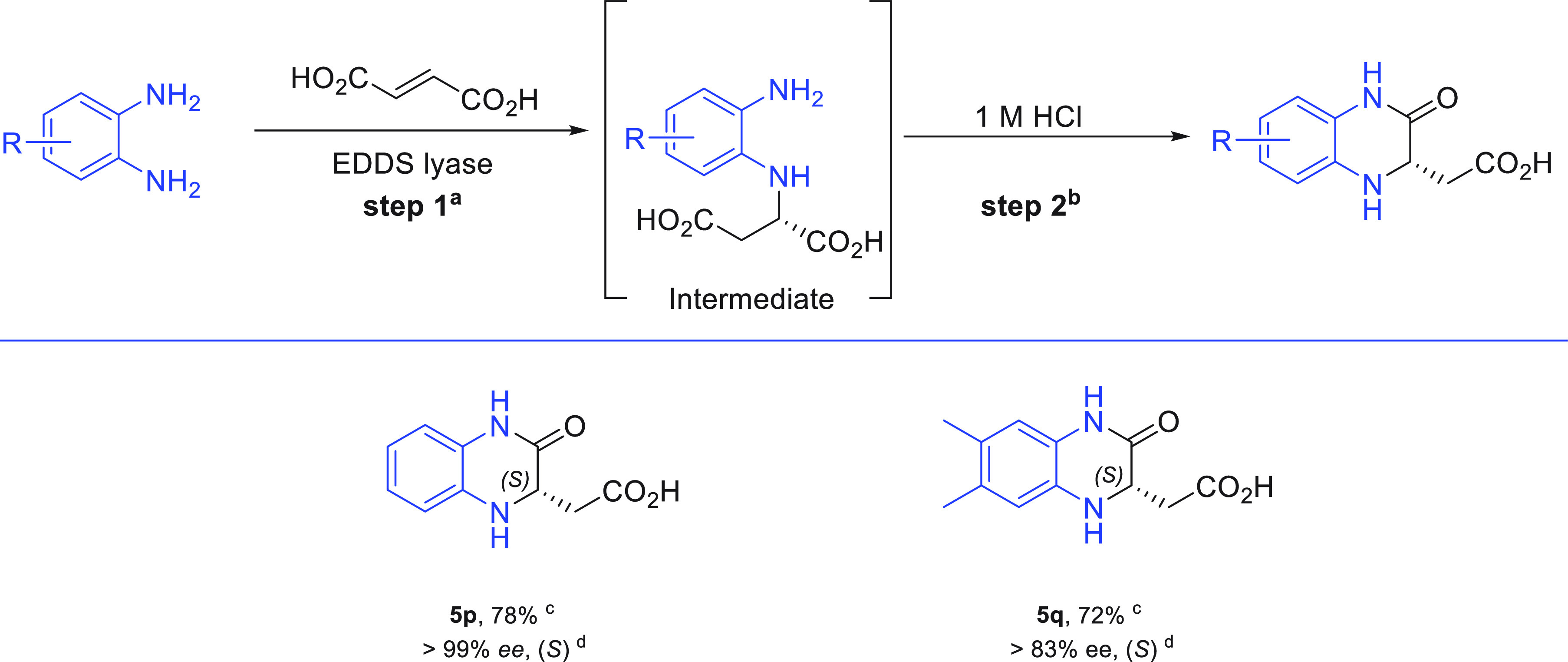
Chemoenzymatic synthesis of DHQs. Reagents and conditions: (a) The reaction mixture (40 mL) consisted of fumaric acid (**2**, 100 mM), diamine substrate **1p** or **1q** (25 mM), and EDDS lyase (0.05 mol % compared to diamine) in buffer (50 mM NaH_2_PO_4_/NaOH, pH 8.5, argon flushed), with DMSO (5%) as cosolvent at room temperature. A 5-fold excess of **2** (instead of an excess of amine) was used, accelerating product purification and avoiding enzyme inhibition as a result of high diamine substrate concentration. (b) Fuming HCl (1.6 mL) was used to adjust pH to 1 at 0 °C, and the reaction was continued for 3 h at room temperature. (c) Isolated yield after reverse-phase chromatography. (d) The enantiomeric excess (ee) was determined by HPLC on a chiral stationary phase using racemic standards. The absolute configuration of **5p** was assigned *S* using chiral HPLC by comparison with an authentic reference compound, and for **5q** based on analogy and in comparison with chiral HPLC data of a chemically synthesized racemic reference.

In conclusion, we developed convenient chemoenzymatic procedures for the rapid asymmetric synthesis of DHBs and DHQs from retrosynthetically designed substrates. These complex heterocycles were obtained with excellent conversion, good isolated yield, and high optical purity (up to >99% ee). It is important to note that, at higher concentrations (>100 mM) of both 2-aminophenols and diamines, we observed precipitation of the enzyme. In future work, we therefore aim to enhance the stability of EDDS lyase by directed evolution, improving its synthetic potential and enabling practical synthesis of DHBs and DHQs at a large scale. In addition, we intend to enlarge the arylamine scope of EDDS lyase by structure-guided protein engineering to access a broader range of enantiopure building blocks, leading to more complex and pharmaceutically important N-containing heterocycles. Current work in our group focuses on screening a large panel of EDDS lyase homologues for obtaining new biocatalysts for asymmetric hydroaminations using bulky arylamines that are not accepted by wild-type EDDS lyase. The results of this database mining approach will be reported in due course.

## Supporting Information Available

Detailed experimental procedures, NMR spectra demonstrating chemical structures, and chiral HPLC spectra of the chemoenzymatically prepared compounds (PDF)

## References

[ref1] aMahaney P. E.; Webb M. B.; Ye F.; Sabatucci J. P.; Steffan R. J.; Chadwick C. C.; Harnish D. C.; Trybulski E. J. Synthesis and Activity of a New Class of Pathway-Selective Estrogen Receptor Ligands: Hydroxybenzoyl-3, 4-Dihydroquinoxalin-2 (1H)-Ones. Bioorg. Med. Chem. 2006, 14 (10), 3455–3466. 10.1016/j.bmc.2006.01.001. 16427291

[ref2] Su S. M. Pyruvate Kinase Activators for Use for Increasing Lifetime of the Red Blood Cells and Treating Anemia. U.S. Patent 9,181,231 B2, 2015.

[ref3] aHayward C. M.; Scully D. A. Squalene Synthetase Inhibitor Agents. U.S. Patent 6,207,664 B1, 2001.

[ref4] Rooney T. P. C.; Filippakopoulos P.; Fedorov O.; Picaud S.; Cortopassi W. A.; Hay D. A.; Martin S.; Tumber A.; Rogers C. M.; Philpott M. A Series of Potent CREBBP Bromodomain Ligands Reveals an Induced-Fit Pocket Stabilized by a Cation−π Interaction. Angew. Chemie Int. Ed. 2014, 53 (24), 6126–6130. 10.1002/anie.201402750. PMC4298791 24821300

[ref5] Gorohovsky S.; Bittner S. Novel N-Quinonyl Amino Acids and Their Transformation to 3-Substituted p-Isoxazinones. Amino Acids 2001, 20 (2), 135–144. 10.1007/s007260170054. 11332448

[ref6] aWolfer J.; Bekele T.; Abraham C. J.; Dogo-Isonagie C.; Lectka T. Catalytic, Asymmetric Synthesis of 1, 4-Benzoxazinones: A Remarkably Enantioselective Route to Α-Amino Acid Derivatives from O-Benzoquinone Imides. Angew. Chemie Int. Ed. 2006, 45 (44), 7398–7400. 10.1002/anie.200602801. 17036371

[ref7] aRueping M.; Antonchick A. P.; Theissmann T. Remarkably Low Catalyst Loading in Brønsted Acid Catalyzed Transfer Hydrogenations: Enantioselective Reduction of Benzoxazines, Benzothiazines, and Benzoxazinones. Angew. Chemie - Int. Ed. 2006, 45 (40), 6751–6755. 10.1002/anie.200601832. 16986184

[ref8] Tanimori S.; Kashiwagi H.; Nishimura T.; Kirihata M. A General and Practical Access to Chiral Quinoxalinones with Low Copper-Catalyst Loading. Adv. Synth. Catal. 2010, 352 (14–15), 2531–2537. 10.1002/adsc.201000323.

[ref9] aYang Y.; Zhao L.; Xu B.; Yang L.; Zhang J.; Zhang H.; Zhou J. Design, Synthesis and Biological Evaluation of Dihydroquinoxalinone Derivatives as BRD4 Inhibitors. Bioorg. Chem. 2016, 68, 236–244. 10.1016/j.bioorg.2016.08.009. 27580186

[ref10] aCarbain B.; Schütznerová E.; Přibylka A.; Krchňák V. Solid-Phase Synthesis of 3, 4-Dihydroquinoxalin-2 (1H)-ones via the Cyclative Cleavage of N-Arylated Carboxamides. Adv. Synth. Catal. 2016, 358 (5), 701–706. 10.1002/adsc.201500826.

[ref11] Poddar H.; de Villiers J.; Zhang J.; Puthan Veetil V.; Raj H.; Thunnissen A.-M. W. H.; Poelarends G. J. Structural Basis for the Catalytic Mechanism of Ethylenediamine-N, N′-Disuccinic Acid Lyase, a Carbon–Nitrogen Bond-Forming Enzyme with a Broad Substrate Scope. Biochemistry 2018, 57 (26), 3752–3763. 10.1021/acs.biochem.8b00406. 29741885 PMC6034166

[ref12] aFu H.; Zhang J.; Saifuddin M.; Cruiming G.; Tepper P. G.; Poelarends G. J. Chemoenzymatic Asymmetric Synthesis of the Metallo-β-Lactamase Inhibitor Aspergillomarasmine A and Related Aminocarboxylic Acids. Nat. Catal. 2018, 1 (3), 186–191. 10.1038/s41929-018-0029-1.

[ref13] aClement J.-B.; Hayes J. F.; Sheldrake H. M.; Sheldrake P. W.; Wells A. S. Synthesis of SB-214857 Using Copper Catalysed Amination of Arylbromides with L-Aspartic Acid. Synlett 2001, 2001 (09), 1423–1427. 10.1055/s-2001-16780.

[ref14] aLi J.-L.; Han B.; Jiang K.; Du W.; Chen Y.-C. Organocatalytic Enantioselective Hetero-Diels–Alder Reaction of Aldehydes and o-Benzoquinone Diimide: Synthesis of Optically Active Hydroquinoxalines. Bioorg. Med. Chem. Lett. 2009, 19 (14), 3952–3954. 10.1016/j.bmcl.2009.03.013. 19318249

