# Supplementary material for: Chemoenzymatic Asymmetric Synthesis of Complex Heterocycles: Dihydrobenzoxazinones and Dihydroquinoxalinones
Source: ACS Catal. 2022 Sep 6;12(18):11421–7. doi: 10.1021/acscatal.2c03008 (PMC9486952; doi:10.1021/acscatal.2c03008)
Supplement: Supplementary file 1 — cs2c03008_si_001.pdf [file cs2c03008_si_001.pdf]

# Supporting Information

# Chemoenzymatic Asymmetric Synthesis of Complex Heterocycles: Dihydrobenzoxazinones and Dihydroquinoxalinones

Mohammad Faizan Bhat,[1] Alejandro Prats Luján,[1] Mohammad Saifuddin,[1] and Gerrit J. Poelarends[*1]

[1]Department of Chemical and Pharmaceutical Biology, Groningen Research Institute of Pharmacy, University of Groningen, Antonius Deusinglaan 1, 9713 AV Groningen, The Netherlands.

*Address correspondence to Prof. Dr. Gerrit J. Poelarends. Tel: +31-50-3633354; Fax: +31-50-3633000; E-mail: g.j.poelarends@rug.nl.

Table of Contents

I) General Information ....................3

II) Detailed experimental procedures ....................4

1.Expression and purification of EDDS lyase. ....................4

2. Substrate scope of EDDS lyase for substituted 2-aminophenols and diamines ....................5

3. Enzymatic synthesis of (*S*)-*N-(*2-hydroxyphenyl)-substituted aspartic acids (**3a-3i**) ....................6

4. Synthesis of chiral dihydrobenzoxazinones (**4a-4h** and **4aa**) ....................9

5. One-pot chemoenzymatic synthesis of chiral dihydroquinoxalinones (**5p, 5q**) ....................13

6. Chemical synthesis of racemic dihydrobenzoxazinone references (**7a-7h**and **7aa**) ....................15

7. Chemical synthesis of racemicdihydroquinoxalinone references (**9, 11**) ....................18

8. Chemical synthesis of chiral reference (*S*)-Methyl 2-(3-oxo-1,2,3,4-tetrahydroquinoxalin-2-yl)acetic acid (**S5**) ....................20

III) NMR Data ....................21

IV) HPLC DATA.. ....................53

References. ....................63

## I) General Information

Fumaric acid, 2-aminophenols and diamines were purchased from Sigma-Aldrich Chemical Co. (St. Louis, MO), TCI Europe N.V., Thermo Fisher Scientific (Geel, Belgium) or Fluorochem Co. (UK). Solvents were purchased from Biosolve (Valkenswaard, The Netherlands) or Sigma-Aldrich Chemical Co. Ingredients for buffers and media were obtained from Duchefa Biochemie (Haarlem, The Netherlands) or Merck (Darmstadt, Germany). Dowex 50W X8 resin (hydrogen form, 100-200 mesh) was purchased from Sigma-Aldrich Chemical Co. Ni sepharose 6 fast flow resin and a HiLoad 16/600 Superdex 200 pg column were purchased from GE Healthcare Bio-Sciences AB (Uppsala, Sweden). Proteins were analyzed by sodium dodecyl sulfate polyacrylamide gel electrophoresis (SDS-PAGE) under denaturing conditions on precast gels (NuPAGE™ 4-12% Bis-Tris protein gels). The gels were stained with Coomassie brilliant blue. High performance liquid chromatography (HPLC) was performed with a Shimadzu LC-10AT HPLC with a Shimadzu SPD-M10A diode array detector. NMR spectra wererecorded on a Bruker500 MHzspectrometer. NMR signals are reported as follows: chemical shift (δ ppm), multiplicity (s = singlet, brs = broad singlet, d = doublet, t = triplet, q = quartet, m = multiplet, dd = doublet of doublet), coupling constant (Hz), and integration. Data for $^{13}C$ NMR are reported in terms of chemical shift relative to Chloroform-*d*(77 ppm), Methanol-$d_4$(49 ppm). Electrospray ionization orbitrap high resolution mass spectrometry (HRMS) was performed by the Mass Spectrometry core facility of the University of Groningen.

## II) Detailed experimental procedures

### 1. Typical procedure for expression and purification of EDDS lyase.

Expression and purification of the protein was carried out using a previously published method with slight modifications.[1] Briefly, *E. coli* TOP10 cells containing the pBADN (EDDS lyase-His) plasmid were collected from a -80 °C glycerol stock and used to inoculate 2xYT/Amp medium (7 mL). After overnight incubation at 37 °C, the culture was used to inoculate fresh 2xYT/Amp medium (1 L). The cells were grown at 37°C for about 4 h until $OD_{600}$ reached 0.6-0.8. Arabinose (0.1%, w/v) was added to induce enzyme expression. Cultures were grown for 18 h at 18°C with vigorous shaking. Cells were harvested by centrifugation and stored at -20 °C until further use. In a typical purification experiment, 6 g of wet cells (from 1 L culture) were suspended in lysis buffer (20 mL; 50 mM Tris-HCl, 300 mM NaCl, 15 mM imidazole, pH 8.0). Cells were disrupted by sonication for 12 x 40 s (sonicator and sample were cooled down between each cycle) at a 70 W output. The unbroken cells and debris were removed by centrifugation. The cell-free extract was filtered through a pore filter (diameter 0.45 μm), and incubated with Ni-NTA resin (2 mL slurry in a small column, 40 mg/mL binding capacity) at 4 °C for 3 h, which had previously been equilibrated with lysis buffer (15 mL x 2). The unbound proteins were eluted from the column by gravity flow. The column was washed with lysis buffer (15 mL x 2) and washing buffer (15 mL x 2; 50 mM Tris-HCl, 300 mM NaCl, 25 mM imidazole, pH 8.0). Retained proteins were incubated for 30 minutes with elution buffer (5 mL; 50 mM Tris-HCl, 300 mM NaCl, 500 mM imidazole, pH 8.0) and eventually eluted. Fractions were analyzed by SDS-PAGE on gels containing acrylamide (4 - 12%). Fractions containing EDDS lyase were combined and loaded onto a PD-10 Sephadex G-25 gel-filtration column, which was previously equilibrated with reaction buffer A (25 mL; 50 mM $NaH_2PO_4$-NaOH buffer, pH 8.5). Proteins were eluted with buffer A (3.5 mL) and fractions were collected and analyzed by SDS-PAGE on gels containing acrylamide (4 - 12%). The purified enzyme was cryopreserved and stored at -20 °C until further use.

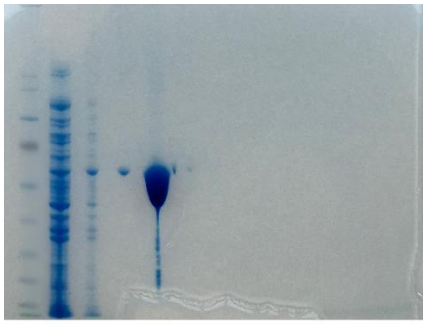


**Figure S0:** Purification of EDDS lyase by Ni-affinity chromatography. Lane 1: Prestained protein ladder (PageRuler™, Thermo Scientific). Lane 2: cell free extract. Lane 3: unbound proteins eluted in flow-through fractions. Lane 4: fraction from washing step with lysis buffer. Lane 5: EDDS lyase containing fraction, eluted with buffer A. The molecular weight of EDDS lyase with the His-tag is approximately 56 kDa.

## 2. Substrate scope of EDDS lyase for substituted 2-aminophenols and diamines

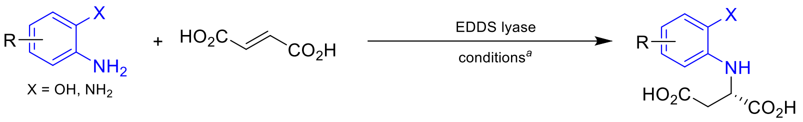


**1a-1zd** **2** **3a-3zd**

**Table S1.**Screening of *O*- and *N*- substituted aromatic amines as substrates for EDDS lyase.

| Entry | Ar | Conv.[b] [%] | Entry | Ar | Conv.[b] [%] | Entry | Ar | Conv.[b] [%] |
|---|---|---|---|---|---|---|---|---|
| 1 | 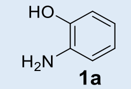 **1a** | 92 | 11 | 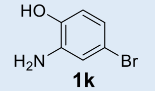 **1k** | 0 | 21 | 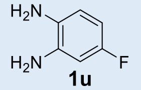 **1u** | 49[c] |
| 2 | 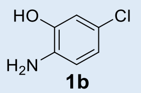 **1b** | 82 | 12 | 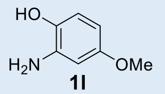 **1l** | 0 | 22 | 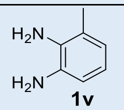 **1v** | 67[c] |
| 3 | 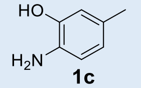 **1c** | 85 | 13 | 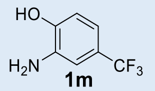 **1m** | 0 | 23 | 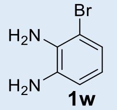 **1w** | 31[c] |
| 4 | 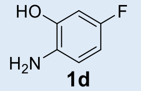 **1d** | 79 | 14 | 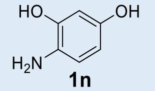 **1n** | 0 | 24 | 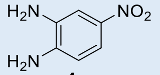 **1x** | 39[c] |
| 5 | 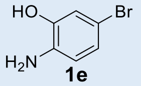 **1e** | 75 | 15 | 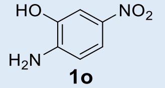 **1o** | 0 | 25 | 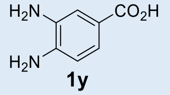 **1y** | 89[c] |
| 6 | 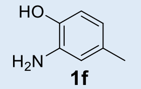 **1f** | 86 | 16 | 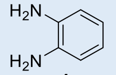 **1p** | 78 | 26 | 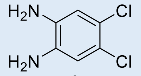 **1z** | 0 |
| 7 | 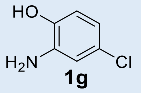 **1g** | 66 | 17 | 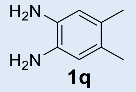 **1q** | 72 | 27 | 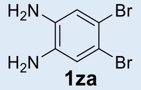 **1za** | 0 |
| 8 | 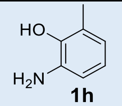 **1h** | 86 | 18 | 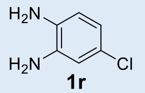 **1r** | 68[c] | 28 | 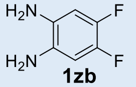 **1zb** | 0 |
| 9 | 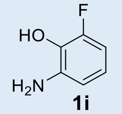 **1i** | 67[d] | 19 | 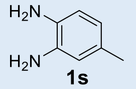 **1s** | 61[c] | 29 | 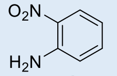 **1zc** | 0 |
| 10 | 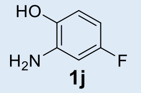 **1j** | 0 | 20 | 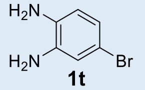 **1t** | 52[c] | 30 | 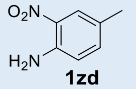 **1zd** | 0 |

[a]Conditions and reagents: amine (**1a-1zd**, 10 mM), EDDS lyase (0.05 mol% based on amine), fumaric acid **2** (50 mM) in degassed buffer (50 mM $NaH_2PO_4$/NaOH, pH 8.5), with 5% DMSO as co-solvent at room temperature.[b]Conversions were determined by comparing $^1H$ NMR signals of substrates and products. [c]Crude samples were analyzed by $^1H$ NMR but further product purification/cyclization proved difficult and requires optimization.[d]Cyclization could not be achieved.

**3. Procedure for the enzymatic synthesis of (*S*)-*N*-(2-hydroxyphenyl)-substituted aspartic acids (3a-3i)**

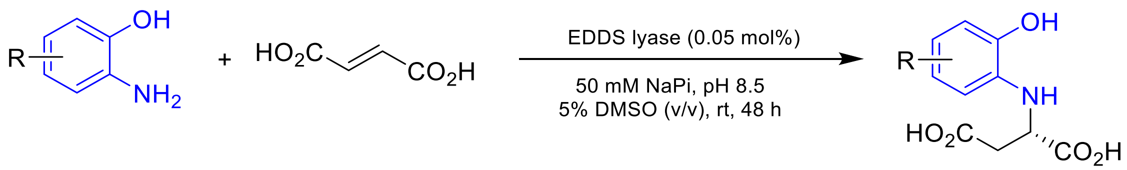


**1a-1i** **2** **3a-3i**

**General Procedure:**A mixture of amine(**1a-1i**, 25 mM) and fumaric acid (100 mM) was dissolved in 50 mM $NaH_2PO_4$-NaOH buffer (pH = 8.5) with 5% DMSO as co-solvent. The pH was adjusted to 8.5 followed by the addition of freshly purified EDDS lyase(0.05mol%).The final volume was adjusted to 40mL with the same buffer and the reaction mixture was incubated at room temperature for 48h. The corresponding amino-acid products werepurified by extraction with 3x40mL EtOAc, followed by cation exchange chromatography. The acidified product was loaded onto the activated resin three times for optimal binding and thenthe column was washed with water (7 column volumes) to remove unreacted fumaric acid and salts (monitored bypH paper until pH=7), and the final product was eluted with 2 M aqueous ammonia solution. All ninhydrin positive fractions were collected, concentrated*in vacuo* and lyophilized to give the desired products**3a-3i**as ammonia salts in 49% to 76% isolated yield.

***(S)*-N-(2-hydroxyphenyl)aspartic acid (3a)**

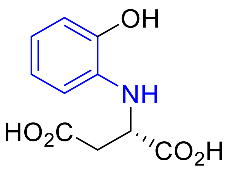


Brown solid;165mg (73% yield).$^{1}$H NMR (500 MHz, Methanol-*d₄*) δ 6.72 – 6.64 (m, 3H), 6.59 – 6.53 (m, 1H), 4.08 (dd, *J* = 7.7, 5.4 Hz, 1H), 2.74 (dd, *J* = 15.2, 5.4 Hz, 1H), 2.62 (dd, *J* = 15.2, 7.7 Hz, 1H); $^{13}$C NMR (126 MHz, $D_2O$) δ 181.43, 179.56, 144.15, 136.50, 121.13, 118.85, 114.96, 113.54, 57.51, 41.12; HRMS (ESI$^+$): calcd. for $C_{10}H_{12}NO_5$[M+H]$^+$: 226.0710, found: 226.0709.

***(S)*-N-(4-chloro-2-hydroxyphenyl)aspartic acid (3b)**

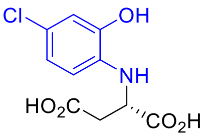


Brown solid; 141mg (72% yield).$^{1}$H NMR (500 MHz, $D_2O$) δ 6.98 (d, *J* = 8.5 Hz, 1H), 6.96 – 6.91 (m, 1H), 6.87 (dd, *J* = 8.6, 2.3 Hz, 1H), 4.40 (t, *J* = 5.5 Hz, 1H), 2.99 – 2.86 (m, 2H); $^{13}$C NMR (126 MHz, $D_2O$) δ 181.86, 179.74, 168.35, 156.27, 138.46, 122.42, 115.72, 112.60, 58.22, 41.53; HRMS (ESI$^+$): calcd. for $C_{10}H_{11}ClNO_5$[M+H]$^+$: 260.0320, found: 260.0322.

**(S)-N-(2-hydroxy-4-methylphenyl)aspartic acid (3c)**

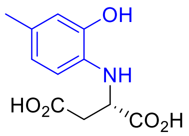


Yellow solid;160mg (66% yield).$^1$H NMR (500 MHz, Methanol-*d₄*) δ 6.65 (d, *J* = 7.9 Hz, 1H), 6.61 – 6.55 (m, 1H), 6.50 (dd, *J* = 8.0, 1.9 Hz, 1H), 3.99 (dd, *J* = 8.9, 4.4 Hz, 1H), 2.71 (dd, *J* = 15.1, 4.4 Hz, 1H), 2.57 (dd, *J* = 15.1, 9.0 Hz, 1H), 2.17 (s, 3H); $^{13}$C NMR (126 MHz, Methanol-d4) δ 182.00, 180.13, 148.09, 135.51, 130.29, 121.71, 121.48, 116.76, 60.76, 42.86, 21.17. HRMS (ESI$^+$): calcd. for $C_{11}H_{14}NO_5[M+H]^+$: 240.0866, found: 240.0863.

**(S)-N-(4-fluoro-2-hydroxyphenyl)aspartic acid (3d)**

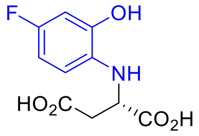


Brown solid;129 mg (53% yield).$^1$H NMR (500 MHz, Methanol-*d₄*) δ 6.69 – 6.63 (m, 1H), 6.51 – 6.44 (m, 1H), 6.43 – 6.35 (m, 1H), 4.00 (dd, *J* = 7.5, 5.5 Hz, 1H), 2.74 (dd, *J* = 15.6, 5.5 Hz, 1H),2.62 (dd, *J* = 15.7, 7.4 Hz, 1H);$^{13}$C NMR (126 MHz, Methanol-*d₄*) δ 181.10, 179.36, 158.18 (d, *J*= 235.0 Hz), 149.15 (d, *J* = 11.1 Hz), 133.88, 116.84 (d, *J* = 9.4 Hz), 106.04 – 105.17 (*m*), 102.90 (t, *J* = 27.0 Hz), 60.32, 41.9; HRMS (ESI$^+$): calcd. for $C_{10}H_{11}FNO_5[M+H]^+$: 244.0618, found: 244.0614.

**(S)-N-(4-bromo-2-hydroxyphenyl)aspartic acid (3e)**

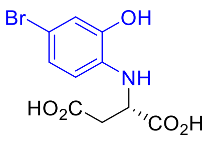


Brown solid; 163 mg (53% yield). $^1$H NMR (500 MHz, $D_2O$) δ 6.61 (dd, *J* = 8.4, 1.4 Hz, 1H), 6.54 (dd, *J* = 8.4, 2.4 Hz, 1H), 6.49 (d, *J* = 2.5 Hz, 1H), 3.96 (dd, *J* = 10.3, 3.8 Hz, 1H), 2.65 (dd, *J* = 14.9, 3.9 Hz, 1H), 2.41 (dd, *J* = 14.8, 10.3 Hz, 1H); $^{13}$C NMR (126 MHz, $D_2O$) δ 181.06, 179.44, 138.48, 123.85, 117.48, 115.48, 111.92, 111.58, 57.69, 41.05; HRMS (ESI$^+$): calcd. for $C_{10}H_{11}BrNO_5[M+H]^+$: 303.9815, found: 303.9813.

**(S)-N-(2-hydroxy-5-methylphenyl)aspartic acid (3f)**

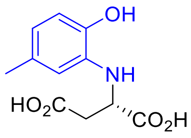


Yellow solid; 172mg (72% yield).$^1$H NMR (500 MHz, $D_2O$) δ 6.81 – 6.72 (m, 1H), 6.69 – 6.55 (m, 2H), 4.10 (dd, *J* = 9.1, 4.2 Hz, 1H), 2.71 (dd, *J* = 15.4, 4.1 Hz, 1H), 2.56 (dd, *J* = 15.4, 9.0 Hz, 1H), 2.19 (s, 3H); $^{13}$C NMR (126 MHz, $D_2O$) δ 179.78, 178.91, 142.72, 133.75, 131.04, 121.13, 116.42, 115.37, 58.64, 39.69, 19.84; HRMS (ESI$^+$): calcd. for $C_{11}H_{14}NO_5[M+H]^+$: 240.0866, found: 240.0866.

**(S)-N-(5-chloro-2-hydroxyphenyl)aspartic acid (3g)**

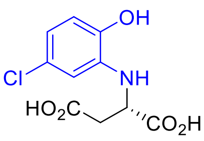


Brown solid; 51 mg (49% yield). $^1$H NMR (500 MHz, $D_2O$) δ 6.61 (dd, *J* = 8.4, 1.4 Hz, 1H), 6.54 (dd, *J* = 8.4, 2.4 Hz, 1H), 6.49 (d, *J* = 2.5 Hz, 1H), 3.96 (dd, *J* = 10.3, 3.8 Hz, 1H), 2.65 (dd, *J* = 14.9, 3.9 Hz, 1H), 2.41 (dd, *J* = 14.8, 10.3 Hz, 1H).; $^{13}$C NMR (126 MHz, $D_2O$) δ 181.06, 179.44, 138.48, 123.85, 117.48, 115.66, 111.92, 111.58, 57.69, 41.05; HRMS (ESI+): calcd. for $C_{10}H_{11}ClNO_5$[M+H]+: 260.0320, found: 260.0320.

**(S)-N-(2-hydroxy-3-methylphenyl)aspartic acid (3h)**

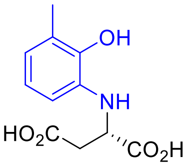


Yellow solid;176mg (76% yield).$^1$H NMR (500 MHz, $D_2O$) δ 6.87 – 6.78 (m, 1H), 6.79 – 6.73 (m, 1H), 6.72 – 6.65 (m, 1H), 4.05 (m, 1H), 2.70 (m, 1H), 2.55 (m, 1H), 2.19 (s, 3H); $^{13}$C NMR (126 MHz, $D_2O$) δ 179.70, 178.89, 143.22, 134.55, 126.41, 123.20, 121.44, 114.14, 58.63, 39.57, 15.40; HRMS (ESI$^+$): calcd. for $C_{11}H_{14}NO_5$[M+H]$^+$: 240.0866, found: 240.0867.

**(S)-N-(3-fluoro-2-hydroxyphenyl)aspartic acid(3i)**

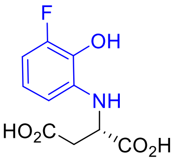


Brown solid;22mg (52% yield). $^1$H NMR (500 MHz, $D_2O$) δ 6.80 – 6.71 (m, 1H), 6.60 – 6.50 (m, 1H), 6.46 – 6.36 (m, 1H), 4.05 (dd, *J* = 10.4, 3.8 Hz, 1H), 2.69 (dd, *J* = 14.8, 3.8 Hz, 1H), 2.44 (dd, *J* = 14.9, 10.4 Hz, 1H); $^{13}$C NMR (126 MHz, $D_2O$) δ 181.21, 179.43, 152.04 (d, *J* = 235.2 Hz), 139.15 (d, *J* = 4.6 Hz), 131.40 (d, *J* = 16.9 Hz), 120.53, 109.37 – 107.89 (m), 106.63 – 104.59 (m), 58.02, 41.03.HRMS (ESI$^+$): calcd. for $C_{10}H_{11}FNO_5$[M+H]$^+$: 244.0616, found: 244.0615.

**4. Synthesis of chiral dihydrobenzoxazinones (4a-4h and 4aa)**

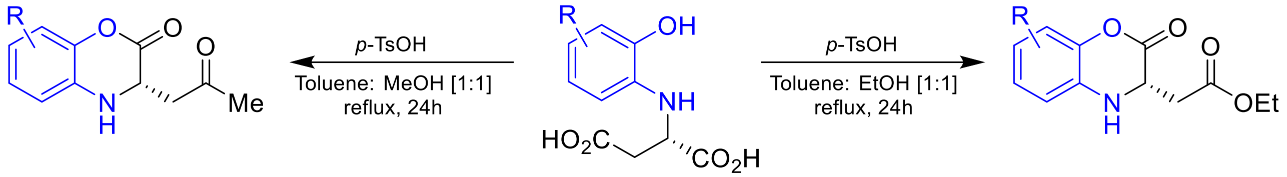


**4aa** **3a-3h** **4a-4h**

**General Procedure:**The purified product (**3a-3h**) was dissolved in a 1:1 mixture of Toluene/EtOH(MeOH was used as a co-solvent for **4aa** instead of EtOH) and to this 1 equiv. of *p*-toluenesulfonic acidwas added. The reaction mixture was slowly heated to reflux, and kept at the same temperature for further 24h under nitrogen atmosphere. After 24h, excess ethanol was removed and the reaction continued in dry toluene (3mL) till the reaction reached completion (monitored by TLC in 20% EtoAc/Pet. ether). The reaction was stopped and the crude reaction mixture dried *in vacuo* and directly loaded on silica gel. The enzymatic product was purified with a gradient ranging from 5% EtOAc/Pet. ether to 20% EtoAc/Pet. ether to give the desired compound (**4a-4h**, and **4aa**) in 46% to 86% yield. The spectral dataare in agreement with previously published data.

**(*S*)-Ethyl 2-(2-oxo-3,4-dihydro-2H-benzo[b][1,4]oxazin-3-yl)acetate (4a)**

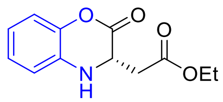

White solid;78 mg (86% yield).$^1$H NMR (500 MHz, Chloroform-*d*) δ 7.09 – 6.93 (m, 2H), 6.88 – 6.81 (m, 1H), 6.82 – 6.68 (m, 1H), 4.71 (*brs*, 1H), 4.28 (dt, *J* = 10.3, 2.9 Hz, 1H), 4.25 – 4.11 (m, 2H), 3.16 (dd, *J* = 17.5, 3.0 Hz, 1H), 2.81 (dd, *J* = 17.5, 10.2 Hz, 1H), 1.29 (t, *J* = 7.2 Hz, 3H);$^{13}$C NMR (126 MHz, Chloroform-*d*) δ 171.60, 166.00, 141.32, 132.72, 125.54, 120.91, 117.22, 115.77, 61.75, 51.77, 35.97, 14.47; HRMS (ESI$^+$): calcd. for $C_{12}H_{14}O_4N$ [M+H]$^+$: 236,0917, found: 236.0911. The chiral HPLC analysis was done on a Chiralcel OD-H column, with isocratic heptane/isopropanol (99%, v/v) as mobile phase with a flow rate of 1 mL/min, rt, UV detection at 210 nm, $t_R$ (*R*, minor) = 19.3 min, $t_R$ (*S*, major) = 20.6 min.The *ee* was determined to be >99% by chiral HPLC analysis using racemic standard and the absolute configuration was assigned (*S*) based on retention pattern of previously published chiral HPLC data[2].

**(*S*)-Ethyl 2-(7-chloro-2-oxo-3,4-dihydro-2H-benzo[b][1,4]oxazin-3-yl)acetate (4b)**

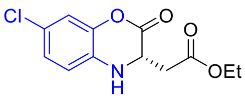

Yellow solid;56mg (77% yield).$^{1}$H NMR (500 MHz, Chloroform-*d*) δ 7.06 (d, *J* = 2.2 Hz, 1H), 7.01 (dd, *J* = 8.5, 2.2 Hz, 1H), 6.75 (d, *J* = 8.4 Hz, 1H), 4.83 (s, 1H), 4.33 – 4.18 (m, 3H), 3.19 (dd, *J* = 17.6, 2.8 Hz, 1H), 2.84 (dd, *J* = 17.6, 10.4 Hz, 1H), 1.33 (t, *J* = 7.2 Hz, 3H); $^{13}$C NMR (126 MHz, Chloroform-*d*) δ 171.08, 164.72, 141.00, 130.95, 125.00, 124.97, 117.08, 116.00, 61.41, 51.14, 35.41, 14.00; HRMS (ESI$^{+}$): calcd. for $C_{12}H_{13}O_4NCl$ $[M+H]^{+}$: 270.0528, found: 270.0528. The chiral HPLC analysis was done on a Chiralcel OD-H column, with isocratic heptane/isopropanol (98%, v/v) as mobile phase with a flow rate of 1 mL/min, rt, UV detection at 210 nm, $t_R$ (*R*, minor) = 17.2 min, $t_R$ (*S*, major) = 25.5 min. The *ee* was determined to be >99% by chiral HPLC analysis using racemic standard and the absolute configuration was assigned (*S*) based on retention pattern of previously published chiral HPLC data[2].

**(*S*)-Ethyl 2-(7-methyl-2-oxo-3,4-dihydro-2H-benzo[b][1,4]oxazin-3-yl)acetate (4c)**

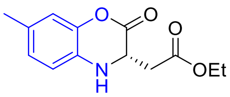

White solid;61mg (84% yield).$^{1}$H NMR (500 MHz, Chloroform-*d*) δ 6.85 – 6.79 (m, 2H), 6.69 (d, *J* = 7.9 Hz, 1H), 4.63 (*brs*, 1H), 4.35 – 4.13 (m, 3H), 3.15 (dd, *J* = 17.5, 2.9 Hz, 1H), 2.80 (dd, *J* = 17.5, 10.2 Hz, 1H), 2.27 (s, 3H), 1.29 (t, *J* = 7.1 Hz, 3H); $^{13}$C NMR (126 MHz, Chloroform-*d*) δ 171.67, 166.27, 141.36, 130.96, 130.18, 126.02, 117.63, 115.71, 61.73, 52.00, 35.89, 20.98, 14.50; HRMS (ESI$^{+}$): calcd. for $C_{13}H_{16}O_4N$ $[M+H]^{+}$: 250.1074, found: 250.1070.The chiral HPLC analysis was done on a Chiralcel OD-H column, with isocratic heptane/isopropanol (95%, v/v) as mobile phase with a flow rate of 1 mL/min, rt, UV detection at 210 nm, $t_R$ (*R*, minor) = 9.6 min, $t_R$ (*S*, major) = 10.9 min. The *ee* was determined to be 98% by chiral HPLC analysis using racemic standard and the absolute configuration was assigned (*S*) based on retention pattern of previously published chiral HPLC data[2].

**(*S*)-Ethyl 2-(7-fluoro-2-oxo-3,4-dihydro-2H-benzo[b][1,4]oxazin-3-yl)acetate (4d)**

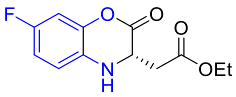

Yellow solid;49 (67% yield).$^{1}$H NMR (500 MHz, Chloroform-*d*) δ 6.80 – 6.77 (m, 1H), 6.74 (d, *J* = 1.6 Hz, 1H), 6.73 (d, *J* = 1.6 Hz, 1H), 4.86 – 4.47 (*brs*, 1H), 4.28 – 4.13 (m, 3H), 3.15 (dd, *J* = 17.5, 2.9 Hz, 1H), 2.80 (dd, *J* = 17.5, 10.1 Hz, 1H), 1.29 (t, *J* = 7.2 Hz, 3H);$^{13}$C NMR (126 MHz, Chloroform-*d*) δ 171.01, 164.98, 156.72 (d, *J* = 239.7 Hz), 140.98 (d, *J* = 11.8 Hz), 128.60 (d, *J* = 2.8 Hz), 115.73 (d, *J* = 8.9 Hz), 111.50 (d, *J* = 22.7 Hz), 104.63 (d, *J* = 26.9 Hz), 61.29, 51.25, 35.23, 13.93; HRMS (ESI$^{+}$): calcd. for $C_{12}H_{13}O_4NF$ $[M+H]^{+}$: 254.0823, found254.0821. The chiral HPLC analysis was done on a Chiralcel OD-H column, with isocratic heptane/isopropanol (95%, v/v,) as mobile phase with a flow rate of 1 mL/min, rt, UV detection at 210 nm, $t_R$ (*R*, minor) = 10.5 min, $t_R$ (*S*, major) = 11.7 min. The *ee*

was determined to be >99% by chiral HPLC analysis using racemic standard and the absolute configuration was tentatively assigned (*S*) based on analogy.

**(*S*)-Ethyl 2-(7-bromo-2-oxo-3,4-dihydro-2H-benzo[b][1,4]oxazin-3-yl)acetate (4e)**

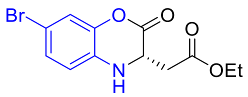


Brown solid; 69 mg (69% yield). $^{1}$H NMR (500 MHz, Chloroform-*d*) δ 7.19 (d, *J* = 2.1 Hz, 1H), 7.14 (dd, *J* = 8.4, 2.2 Hz, 1H), 6.70 (d, *J* = 8.4 Hz, 1H), 4.91 – 4.74 (*brs*, 1H), 4.30 (m, 1H), 4.27 – 4.20 (m, 2H), 3.19 (dd, *J* = 17.5, 2.8 Hz, 1H), 2.84 (dd, *J* = 17.5, 10.3 Hz, 1H), 1.32 (t, *J* = 7.1 Hz, 3H), $^{13}$C NMR (126 MHz, Chloroform-*d*) δ 171.03, 164.61, 141.22, 131.41, 127.87, 119.84, 116.41, 111.58, 61.40, 51.11, 35.46, 13.99; HRMS (ESI$^{+}$): calcd. for $C_{12}H_{13}O_4NBr$ $[M+H]^{+}$: 314.0022, found314,0021. Because of difficulties in synthesizing a racemic standard, the ee was not determined. The absolute configuration was tentatively assigned (*S*) based on analogy.

**(*S*)-Ethyl 2-(6-methyl-2-oxo-3,4-dihydro-2H-benzo[b][1,4]oxazin-3-yl)acetate (4f)**

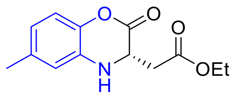


White solid;62mg(85% yield).$^{1}$H NMR (500 MHz, Chloroform-*d*) δ 6.89 (d, *J* = 8.2 Hz, 1H), 6.67 – 6.58 (m, 2H), 4.67 (*brs*, 1H), 4.33 – 4.13 (m, 3H), 3.15 (dd,*J* = 17.5, 2.8 Hz,1H), 2.80 (dd, *J* = 17.5, 2.8 Hz, 1H), 2.26 (s, 3H), 1.29 (t, *J* = 7.2 Hz, 3H); $^{13}$C NMR (126 MHz, Chloroform-*d*) δ 171.65, 166.17, 139.35, 135.42, 132.38, 121.51, 116.92, 116.24, 61.74, 51.84, 35.95, 21.30, 14.49; HRMS (ESI$^{+}$): calcd. for $C_{13}H_{16}O_4N$ $[M+H]^{+}$: 250.1074, found250.1071. The chiral HPLC analysis was done on a Chiralcel AD-H column, with isocratic heptane/isopropanol (98%, v/v) as mobile phase with a flow rate of 1 mL/min, rt, UV detection at 210 nm, $t_R$ (*R*, minor) = 21.6 min, $t_R$ (*S*, major) = 23.4 min. The *ee* was determined to be 86% by chiral HPLC analysis using racemic standard and the absolute configuration was assigned (*S*) based on retention pattern of previously published chiral HPLC data[2].

**(*S*)-Ethyl 2-(6-Choro-2-oxo-3,4-dihydro-2H-benzo[b][1,4]oxazin-3-yl)acetate (4g)**

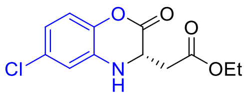


White solid; 12 mg (46% yield). $^{1}$H NMR (500 MHz, Chloroform-*d*) δ 7.00 – 6.85 (m, 1H), 6.76 – 6.56 (m, 2H), 4.35 – 4.16 (m, 3H), 3.18 (dd, *J* = 17.5, 2.9 Hz, 1H), 2.81 (dd, *J* = 17.5, 10.3 Hz, 1H), 2.29 (s, 3H), 1.30 (t, *J* = 7.1 Hz, 3H); $^{13}$C NMR (126 MHz, Chloroform-*d*) δ 171.02, 164.78, 139.26, 133.10, 130.09, 120.18, 117.77, 115.08, 61.44, 50.93, 35.45, 14.00; HRMS (ESI$^{+}$): calcd. for $C_{12}H_{13}O_4NCl$ $[M+H]^{+}$: 270.0528, found270.0527. The chiral HPLC analysis was done on a Chiralcel AD-H column, with isocratic heptane/isopropanol (97%, v/v) as mobile phase with a flow rate of 1 mL/min, rt, UV detection at 210 nm, $t_R$ (*S*, major) = 27.5 min, $t_R$ (*R*, minor) = 39.4 min. The *ee* was determined to be >99% by chiral HPLC analysis using racemic standard and the absolute configuration was assigned (*S*) based on retention pattern of previously published chiral HPLC data[2].

**(*S*)-Ethyl 2-(8-methyl-2-oxo-3,4-dihydro-2H-benzo[b][1,4]oxazin-3-yl)acetate (4h)**

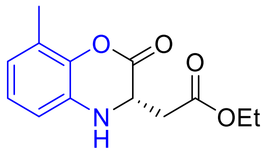


Brown solid;45mg (70% yield).$^{1}$H NMR (500 MHz, Chloroform-*d*) δ 6.90 (t, *J* = 7.7 Hz, 1H), 6.78 – 6.66 (m, 1H), 6.64 (m, *J* = 7.9, 1.5 Hz, 1H), 4.26 – 4.15 (m, 3H), 3.18 (dd, *J* = 17.5, 2.9 Hz, 1H), 2.81 (dd, *J* = 17.6, 10.3 Hz, 1H), 2.29 (s, 3H), 1.29 (t, *J* = 7.1 Hz, 3H); $^{13}$C NMR (126 MHz, Chloroform-*d*)) δ 171.67, 166.18, 139.80, 132.65, 126.83, 124.90, 122.71, 113.46, 61.73, 51.70, 35.90, 15.73, 14.49; HRMS (ESI$^{+}$): calcd. for $C_{13}H_{16}O_4N$ $[M+H]^+$: 250.1074, found250.1073.The chiral HPLC analysis was done on a Chiralpak ID column, with isocratic heptane/isopropanol (95%, v/v) as mobile phase with a flow rate of 1 mL/min, rt, UV detection at 210 nm, $t_R$ (*R*, minor) = 10.5 min, $t_R$ (*S*, major) = 13.2 min. The *ee* was determined to be 90% by chiral HPLC analysis using racemic standard and the absolute configuration was tentatively assigned (*S*) based on analogy.

**(*S*)-Methyl 2-(2-oxo-3,4-dihydro-2H-benzo[b][1,4]oxazin-3-yl)acetate (4aa)**

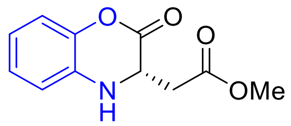


Yellow solid; 50 mg (72% yield). $^{1}$H NMR (500 MHz, Chloroform-*d*) δ 7.06 – 6.99 (m, 2H), 6.90 – 6.84 (m, 1H), 6.82 – 6.78 (m, 1H), 4.73 (s, 1H), 4.29 (dt, *J* = 10.3, 2.7 Hz, 1H), 3.77 (s, 3H), 3.20 (dd, *J* = 17.5, 2.8 Hz, 1H), 2.84 (dd, *J* = 17.5, 10.3 Hz, 1H).$^{13}$C NMR (126 MHz, Chloroform-*d*) δ 171.58, 165.42, 140.88, 132.18, 125.08, 120.52, 116.78, 115.32, 52.16, 51.30, 35.29; HRMS (ESI$^{+}$): calcd. for $C_{11}H_{12}O_4N$ $[M+H]^+$: 222.0761, found: 222.0759. The chiral HPLC analysis was done on a Chiralcel AD-H column, with isocratic heptane/isopropanol (95%, v/v) as mobile phase with a flow rate of 1 mL/min, rt, UV detection at 210 nm, $t_R$ (*S*, major) = 17.8 min, $t_R$ (*R*, minor) = 23.6 min. The *ee* was determined to be >99% by chiral HPLC analysis using racemic standard and the absolute configuration was assigned (*S*) based on retention pattern of previously published chiral HPLC data[2].

## 5. One-pot chemoenzymatic synthesis of chiral dihydroquinoxalinones (5p, 5q)

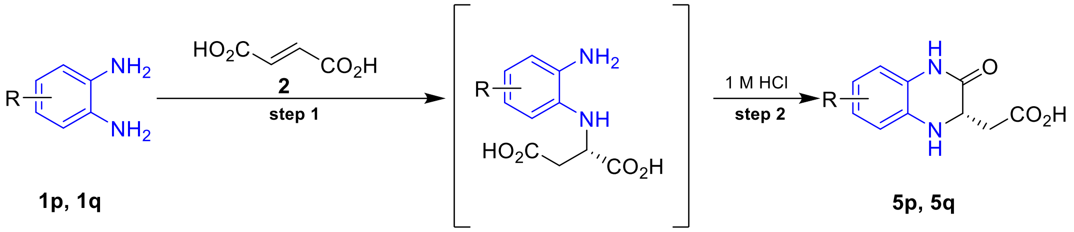


**1p, 1q** **5p, 5q**

**General Procedure:**

**Step 1.**Diamine (**1p**, **1q**, 25 mM) and fumaric acid (100 mM) weredissolved in 20mL degassed buffer (50 mM $NaH_2PO_4$-NaOH, pH 8.5) with 5% DMSO as co-solvent under nitrogen atmosphere. The pH was adjusted to 8.5 and freshly purified EDDS lyase (0.05 mol%) was added to the reaction mixture; the mixture was incubated at room temperature for 72 h. The reaction progress was monitored with $^1$H NMR spectroscopy by comparing the signals of starting material with the final products.After completion, the reaction was extracted with 20mL EtOAc to remove any unreacted amines and subjected to the next step without further purification.

**Step 2.**The reaction mixture was maintained at 0 °C and to this, 1.6 mL fuming HCl was added dropwise. The reaction mixture was then stirred at 0 °C for 30 min, after which the ice-bath was removed and the reaction left at room temperature for 3 h until completion (monitored by $^1$H NMR spectroscopy). The reaction mixture was then concentrated *in vacuo* and the desired product was purified with C18 column chromatography (5% to 50% $CH_3CN$ in $H_2O$ as the eluent) to give **5p** and **5q** in 78% and 72% yield, respectively.

***(S)*-2-(3-oxo-1,2,3,4-tetrahydroquinoxalin-2-yl)acetic acid (5p)**

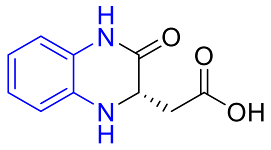


Yellow solid; 57mg (78% yield).$^1$H NMR (500 MHz, Methanol-$d_4$) δ 6.89 – 6.81 (m, 1H), 6.79 – 6.68 (m, 3H), 4.22 (dd, $J$ = 8.3, 4.3 Hz, 1H), 2.86 (dd, $J$ = 16.6, 4.4 Hz, 1H), 2.65 (dd, $J$ = 16.6, 8.2 Hz, 1H); $^{13}$C NMR (126 MHz, Methanol-$d_4$) δ 174.14, 169.23, 134.69, 126.60, 124.53, 119.90, 115.98 (d, $J_{NC}$ = 15.0 Hz), 115.00 (d, $J_{NC}$ = 15.1 Hz), 53.91 (d, $J_{NC}$ = 11.1 Hz), 36.70; HRMS (ESI$^+$): calcd. for $C_{10}H_9O_3N_2$ $[M+H]^+$: 205.0608, found: 205.0617. The chiral HPLC analysis was done on a Chiralcel AD-RH column, with isocratic water/acetonitrile (85%, v/v, 0.1% formic acid) as mobile phase with a flow rate of 1 mL/min, rt, UV detection at 220 nm, $t_R$ (*R*, minor) = 10.6 min, $t_R$ (*S*, major) = 13.7 min. The *ee* was determined to be >99% by chiral HPLC analysis using racemic standard, and the absolute configuration was assigned (*S*) as compared to the authentic (*S*) standard synthesized by following the previously published procedure[5].

**(*S*)-2-(6,7-dimethyl-3-oxo-1,2,3,4-tetrahydroquinoxalin-2-yl)acetic acid(5q)**

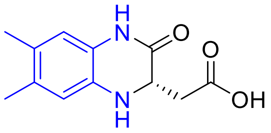

Brown solid; 62mg (72% yield).$^{1}$H NMR (500 MHz, Methanol-$d_4$) δ 6.56 (d, $J$ = 5.6 Hz, 2H), 4.15 (dd, $J$ = 8.5, 4.2 Hz, 1H), 2.82 (dd, $J$ = 16.5, 4.3 Hz, 1H), 2.60 (dd, $J$ = 16.5, 8.5 Hz, 1H), 2.14 (d, $J$ = 2.2 Hz, 6H); $^{13}$C NMR (126 MHz, Methanol-$d_4$) δ 174.25, 169.16, 132.35, 132.14, 127.85, 124.42, 116.98 (d, $J_{NC}$ = 15.7 Hz), 116.40 (d, $J_{NC}$ = 15.9 Hz), 54.09 (d, $J_{NC}$= 13.7 Hz), 36.43, 18.86, 18.52; HRMS (ESI+): calcd. for $C_{12}H_{13}O_3N_2$ [M+H]+:233.0921, found: 233.0918.The chiral HPLC analysis was done on a Chiralcel OD-RH column, with isocratic water/acetonitrile (80%, v/v, 1% AcOH) as mobile phase with a flow rate of 1 mL/min, rt, UV detection at 220 nm, column oven temperature = 60 °C, $t_R$ (*R*, minor) = 4.6 min, $t_R$ (*S*, major) = 7.4 min. The *ee* was determined to be 83% by chiral HPLC analysis using racemic standard, and the absolute configuration was tentatively assigned (*S*) based on analogy.

## 6. Chemical synthesis of racemic dihydrobenzoxazinone references (7a-7g and 7aa)

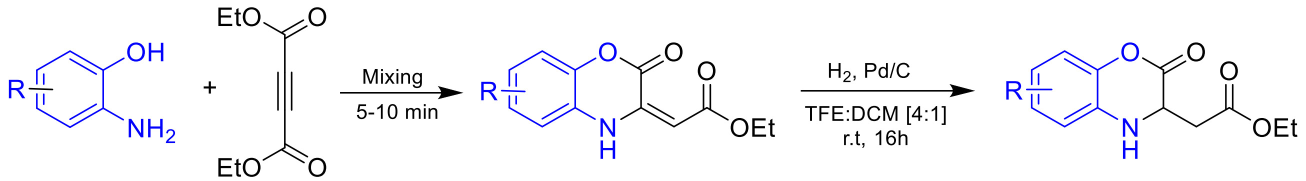


**1a-1h** | **5** or Et = Me for **7aa** | **6a-6h** and Et = Me for **6aa** | **7a-7h** and Et = Me for **7aa**

**General Procedure:**

For the synthesis of racemic standards, a general procedure published elsewhere was followed with slight modifications[2].

**Step 1:**0.2 g of 2-aminophenol(**1a-1h**, 1.83mmol) was placed in a beaker and 1 equiv. of diethyl acetylenedicarboxylate5(dimethylacetylenedicarboxylate for **7aa**)was added dropwise, and stirred with a spatula until a solid and dry product was obtained which was then washed with a few drops of methanol over a filter paper to obtain **6a-6h**and **6aa**in high yields; each product was used in the next step without purification.

**Step 2:**0.2g (0.85 mmol)of the intermediate (**6a-6h**)was dissolved in 8 mL TFE and 2 mL DCM, followed by addition of 10 mol% Pd/C and the reaction was left to stir at room temperature until completion under hydrogen atmosphere (Balloon, 6-16h, monitored by TLC in 20% EtoAc/Pet. ether). The reaction mixture was filtered through a short plug of celite and the desired product purified with flash chromatography over silica gel (gradient from 5% EtOAc to 20% EtOAc/Pet. ether) to obtain racemic products**7a-7h**in 47-79% yield. All spectral data are in agreement with previously published data[2].

**Ethyl 2-(2-oxo-3,4-dihydro-2H-benzo[b][1,4]oxazin-3-yl)acetate (7a)**

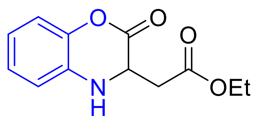

Yellow solid;150mg (74% yield).$^1$H NMR (500 MHz, Chloroform-*d*) δ 7.04 – 6.96 (m, 2H), 6.88 – 6.82 (m, 1H), 6.82 – 6.76 (m, 1H), 4.76 (*brs*, 1H), 4.28 (dt,*J* = 10.3, 2.7,1H), 4.24 – 4.18 (m, 2H), 3.16 (dd, *J* = 17.5, 2.9 Hz, 1H), 2.81 (dd, *J* = 17.5, 10.2 Hz, 1H), 1.29 (t, *J* = 7.2 Hz, 3H); $^{13}$C NMR (126 MHz, Chloroform-*d*) δ 171.11, 165.50, 140.82, 132.22, 125.04, 120.41, 116.73, 115.27, 61.25, 51.27, 35.47, 13.97.

**Ethyl 2-(7-chloro-2-oxo-3,4-dihydro-2H-benzo[b][1,4]oxazin-3-yl)acetate (7b)**

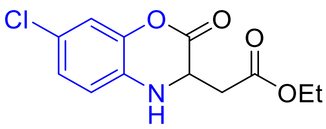


White solid; 120mg (60% yield).$^{1}$H NMR (500 MHz, Chloroform-*d*) δ 7.03 (d, *J* = 2.4 Hz, 1H), 6.98 (dd, *J* = 8.4, 2.3 Hz, 1H), 6.72 (d, *J* = 8.4 Hz, 1H), 4.8 (*brs*, 1H ) 4.30 – 4.17 (m, 3H), 3.17 (dd, *J* = 17.5, 2.8 Hz, 1H), 2.81 (dd, *J* = 17.6, 10.3 Hz, 1H), 1.30 (t, *J* = 7.1 Hz, 3H);$^{13}$C NMR (126 MHz, Chloroform-*d*) δ 171.54, 165.25, 141.43, 131.43, 125.00, 117.51, 116.48, 61.88, 51.57, 35.87, 14.47.

**Ethyl 2-(7-methyl-2-oxo-3,4-dihydro-2H-benzo[b][1,4]oxazin-3-yl)acetate (7c)**

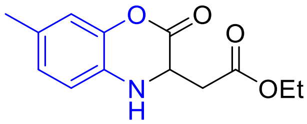


White solid; 160mg (79% yield).$^{1}$H NMR (500 MHz, Chloroform-*d*) δ 6.89 – 6.77 (m, 2H), 6.69 (d, *J* = 7.9 Hz, 1H), 4.62 (*brs*, 1H), 4.32 – 4.13 (m, 3H), 3.16 (dd, *J* = 17.5, 2.8 Hz, 1H), 2.81 (dd, *J* = 17.5, 10.3 Hz, 1H), 2.28 (s, 3H), 1.29 (t, *J* = 7.2 Hz, 3H);$^{13}$C NMR (126 MHz, Chloroform-*d*) δ 171.60, 166.26, 141.28, 130.86, 130.15, 125.96, 117.54, 115.66, 61.68, 51.92, 35.83, 20.93, 14.45.

**Ethyl 2-(7-fluoro-2-oxo-3,4-dihydro-2H-benzo[b][1,4]oxazin-3-yl)acetate (7d)**

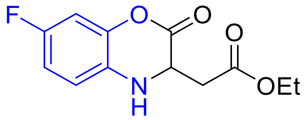


White solid; 143mg (71% yield).$^{1}$H NMR (500 MHz, Chloroform-*d*) δ 6.83 – 6.78 (m, 1H), 6.76 – 6.72 (m, 2H), 4.69 (*brs*, 1H), 4.41 – 3.98 (m, 3H), 3.17 (dd, *J* = 17.5, 2.8 Hz, 1H), 2.81 (dd, *J* = 17.5, 10.2 Hz, 1H), 1.31 (s, 3H);$^{13}$C NMR (126 MHz, Chloroform-*d*) δ 171.14, 165.00, 153.60, 128.58, 122.22, 115.76, 111.59 (d, *J* = 22.6 Hz), 104.75 (d, *J* = 26.8 Hz), 61.38, 51.28, 35.22, 14.01.

**Ethyl 2-(6-methyl-2-oxo-3,4-dihydro-2H-benzo[b][1,4]oxazin-3-yl)acetate (7f)**

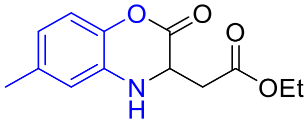


White solid; 135mg (67% yield). $^{1}$H NMR (500 MHz, Chloroform-*d*) δ 6.90 (d, *J* = 8.2 Hz, 1H), 6.67 – 6.58 (m, 2H), 4.36 – 4.14 (m, 3H), 3.15 (dd, *J* = 17.5, 3.6 Hz, 1H), 2.80 (dd, *J* = 17.5, 10.4 Hz, 1H), 2.27 (s, 3H), 1.30 (t, *J* = 7.1 Hz, 3H); $^{13}$C NMR (126 MHz, Chloroform-*d*) δ 171.27, 165.90, 139.00, 135.06, 132.09, 121.12, 116.53, 115.91, 61.39, 51.49, 35.63, 20.96, 14.16.

**Ethyl 2-(6-chloro-2-oxo-3,4-dihydro-2H-benzo[b][1,4]oxazin-3-yl)acetate (7g)**

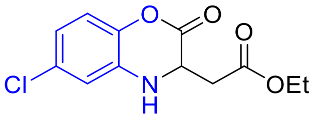


White solid; 94 mg (47% yield).$^{1}$H NMR (500 MHz, Chloroform-*d*) δ 6.94 (d, *J* = 8.5 Hz, 1H), 6.83 – 6.78 (m, 2H), 4.39 – 4.17 (m, 3H), 3.16 (dd, *J* = 17.6, 2.8 Hz, 1H), 2.81 (dd, *J* = 17.6, 10.4 Hz, 1H), 1.30 (t, *J* = 7.1 Hz, 3H); $^{13}$C NMR (126 MHz, Chloroform-*d*) δ 170.85, 164.80, 139.13, 133.07, 129.94, 119.94, 117.61, 114.96, 61.32, 50.82, 35.44, 13.89.

**Ethyl 2-(8-methyl-2-oxo-3,4-dihydro-2H-benzo[b][1,4]oxazin-3-yl)acetate (7h)**

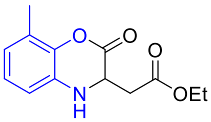


White solid; 153mg (76% yield).$^{1}$H NMR (500 MHz, Chloroform-*d*) δ 6.95 – 6.81 (m, 1H), 6.70 (dd, *J* = 7.6, 1.4 Hz, 1H), 6.63 (dd, *J* = 7.9, 1.4 Hz, 1H), 4.72 (*brs*, 1H), 4.30 – 4.11 (m, 3H), 3.16 (dd, *J* = 17.5, 3.0 Hz, 1H), 2.80 (dd, *J* = 17.5, 10.2 Hz, 1H), 2.28 (s, 3H), 1.29 (t, *J* = 7.2 Hz, 3H); $^{13}$C NMR (126 MHz, Chloroform-*d*) δ 171.49, 166.13, 139.67, 132.61, 126.62, 124.78, 122.52, 113.35, 61.59, 51.56, 35.80, 15.62, 14.37.

**Methyl 2-(2-oxo-3,4-dihydro-2H-benzo[b][1,4]oxazin-3-yl)acetate (7aa)**

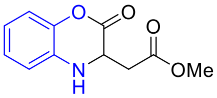


White solid; 153 mg (76% yield). $^{1}$H NMR (500 MHz, Chloroform-*d*) δ 7.12 – 6.94 (m, 1H), 6.90 – 6.83 (m, 1H), 6.80 – 6.74 (m, 1H), 4.29 (dd, *J* = 10.3, 2.8 Hz, 1H), 3.76 (s, 3H), 3.20 (dd, *J* = 17.5, 2.9 Hz, 1H), 2.84 (dd, *J* = 17.5, 10.3 Hz, 1H); $^{13}$C NMR (126 MHz, Chloroform-*d*) δ 171.54, 165.46, 140.79, 132.16, 125.05, 120.45, 116.71, 115.28, 52.16, 51.21, 35.22.

### 7. Synthesis of *rac*- 2-(3-oxo-1,2,3,4-tetrahydroquinoxalin-2-yl)acetic acid (9, 11)

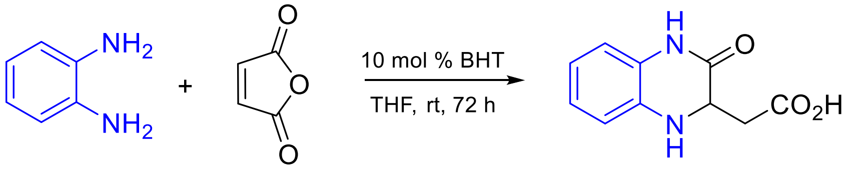


**General procedure.**The synthesis of racemic standard **9**was performed following the procedure published elsewhere with a slight modification[3]. Briefly, **1p**(0.5 g, 4.62 mmol) and BHT (0.2 g, 0.231 mmol)weredissolved in 20mL THF and to this, a 20mL THF solution containing maleic anhydride**8** (0.5 g, 5.09 mmol) was added dropwise and left to stir at room temperature for 72 h. After completion of the reaction (monitored by TLC in EtOAc/Pet ether/formic acid [9:1:0.1] ), the solvent was removed under vacuum and the desired product directly purified with silica gel (gradient from 50% EtOAc/Pet. ether to 90% EtOAc/Pet. ether) to give racemic reference compound **9**in 36% yield. The spectral data is consistent with previously published data[3].

### 7a. Synthesis of *rac*- 2-(6,7-dimethyl-3-oxo-1,2,3,4-tetrahydroquinoxalin-2-yl)acetic acid (11)

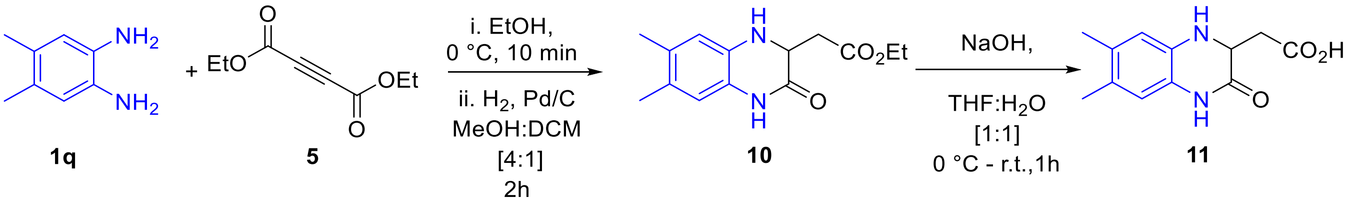


For the synthesis of **11**, a previously published procedurewas followed with modification[4]. Briefly, **1q** (0.5g, 3.67 mmol) was dissolved in 10mL absolute ethanol and stirred at 0 °C, followed by dropwise addition of diethylacetylene dicarboxylate **5**(538 µL, 3.67 mmol). After 10 min reaction time, a yellow precipitate was observed whichwas filtered, washed with 10mL of ethanol, and dried under vaccum. The crude productwas purified with flash chromatography (10% EtoAc/Pet. ether)to give an intermediate imineas yellow fluffy solid (0.83g, 86% yield), that was used immediately for the next step.

Thefreshly obtainedimine (0.5g, 1.92 mmol) was dissolved in 30mL methanol and 6mL DCM [4:1] and to this Pd/C (0.02g, 0.192 mmol) was added and the reaction stirred at room temperature for 2 h under hydrogen atmosphere (balloon). After completion of the reaction (monitored by TLC, 20% EtoAc/Pet. ether, r.f. 0.2), the reaction was stopped, and the reaction

mixture was filtered over a short plug of celite.The filtrate was dried under vacuum to give product **10**as white solid(0.45g, 89% yield),the filtrate was then used in the next step without further purification.

Finally,**10**(0.1g, 0.381 mmol)was dissolved in 3 mL THF and 3 mL $H_2O$at 0 °C (maintained with an ice bath), and to thissodium hydroxide (0.017g, 0.419mmol)was added. After 5 min, the ice bath was removed and the reaction continued for 2 h (monitored by TLC, 50% EtoAc/Pet. ether). THF was removed *in vacuo*,and the aqueous layer lyophilized to give the crude product, which was further purified with silica gel column chromatography (gradient from 10% MeOH/DCM to 50% MeOH/DCM) to give the final racemic standard **11**as a white fluffy solid (0.04g, 45% yield).

**2-(3-oxo-1,2,3,4-tetrahydroquinoxalin-2-yl)acetic acid (9)**

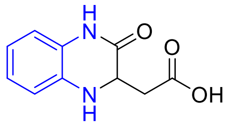


Yellow solid;350mg (36% yield).$^{1}$H NMR (500 MHz, Methanol-$d_4$) δ 6.87 – 6.81 (m, 1H), 6.79 – 6.65 (m, 3H), 4.22 (dd, $J$ = 8.2, 4.3 Hz, 1H), 2.85 (dd, $J$ = 16.6, 4.3 Hz, 1H), 2.64 (dd, $J$ = 16.6, 8.3 Hz, 1H);$^{13}$C NMR (126 MHz, Methanol-$d_4$)δ 174.14, 169.23, 134.69, 126.60, 124.52, 119.84 (d, $J_{NC}$= 15.0 Hz), 115.98 (d, $J_{NC}$ = 15.0 Hz), 115.01 (d, $J_{NC}$= 14.2 Hz), 53.95, 53.87, 36.70.

**2-(6,7-dimethyl-3-oxo-1,2,3,4-tetrahydroquinoxalin-2-yl)acetic acid (11)**

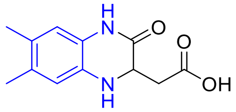


White solid. 20mg (42% yield).$^{1}$H NMR (500 MHz, Methanol-$d_4$) δ 6.55 (d, $J$ = 6.1 Hz, 2H), 4.14 (dd, $J$ = 8.6, 4.2 Hz, 1H), 2.81 (dd, $J$ = 16.5, 4.3 Hz, 1H), 2.59 (dd, $J$ = 16.5, 8.6 Hz, 1H), 2.13 (s, 6H);$^{13}$C NMR (126 MHz, Methanol-$d_4$) δ 179.22, 171.47, 141.63, 133.24, 133.14, 128.71, 125.50, 117.46 (d, $J_{NC}$ = 57.1 Hz), 56.52, 39.86, 19.64.

## 8. Synthesis ofreference (*S*)-2-(3-oxo-1,2,3,4-tetrahydroquinoxalin-2-yl)acetic acid(S5)

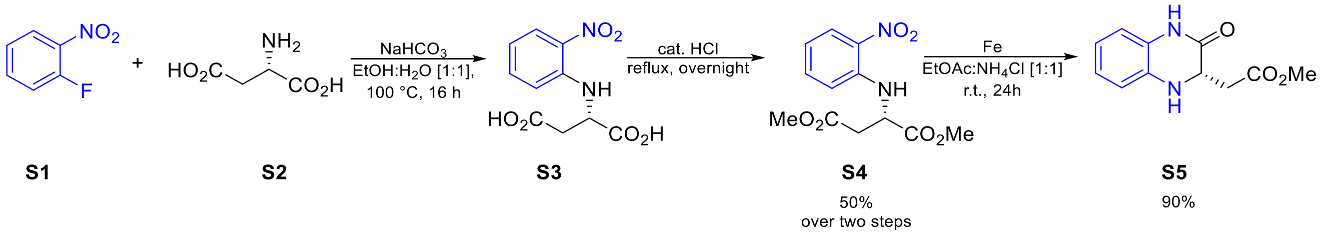


**General procedure.**For the synthesis of reference **S5**,a published procedure was followed with slight modification[5]. Briefly, **S2** (0.5 g, 3.76 mmol) was dissolved in 6mL ethanol and 6mL distilled water followed by addition of $Na_2HCO_3$ (0.63 g, 7.51 mmol) and **S1**(0.58 g, 4.13 mmol);the reaction mixture was then heated to 100 °C and incubated overnight. After completion (monitored by TLC), the reaction mixture was cooled to room temperature, and then extracted with 3x30mL EtOAc. The combined organic layerswere dried under vacuum, and the crude product **S3** was used in the next step without purification. **S3**was dissolved in 10mL methanol, followed by addition of 100 µL of conc. HCl, and left to reflux overnight to give **S4**in quantitative yield, which wasfurther used in the next step without purification. For reduction and cyclization, **S4** was dissolved in 6mL EtOAc and 6mL sat. $NH_4Cl$, and to this reaction mixture Fe (6 equiv.) was added and the mixture was left to stir at room temperature for 24h. After completion of the reaction (monitored by TLC in 50% EtOAC/Pet. ether), the reaction mixture was extracted with 3x30mL EtOAc. The organic layerswere combined and dried with $Na_2SO_4$,followed by evaporation*in vacuo* to give crude product, whichwas then further purified by flash chromatography (from 10% to 50% EtOAc/hexanes gradient) to give **S5**in 90% yield. The NMR data is in agreement with NMR data reported in the literature[5].

### (*S*)-Methyl 2-(3-oxo-1,2,3,4-tetrahydroquinoxalin-2-yl)acetate(S5)

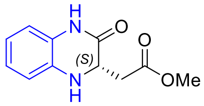


Yellow solid; 257mg (45% yield).$^1$H NMR (500 MHz, Chloroform-*d*) δ 9.58 (s, 1H), 6.96 – 6.89 (m, 1H), 6.84 – 6.76 (m, 2H), 6.75 – 6.68 (m, 1H), 4.39 (dd, *J* = 10.4, 2.7 Hz, 1H), 3.77 (s, 3H), 3.16 (dd, *J* = 17.3, 2.8 Hz, 1H), 2.79 (dd, *J* = 17.3, 10.4 Hz, 1H); $^{13}$C NMR (126 MHz, Chloroform-*d*)) δ 172.21, 167.64, 132.61, 124.89, 123.95, 119.55, 115.51, 114.28, 52.62, 51.97, 35.65.

## III) NMR Data

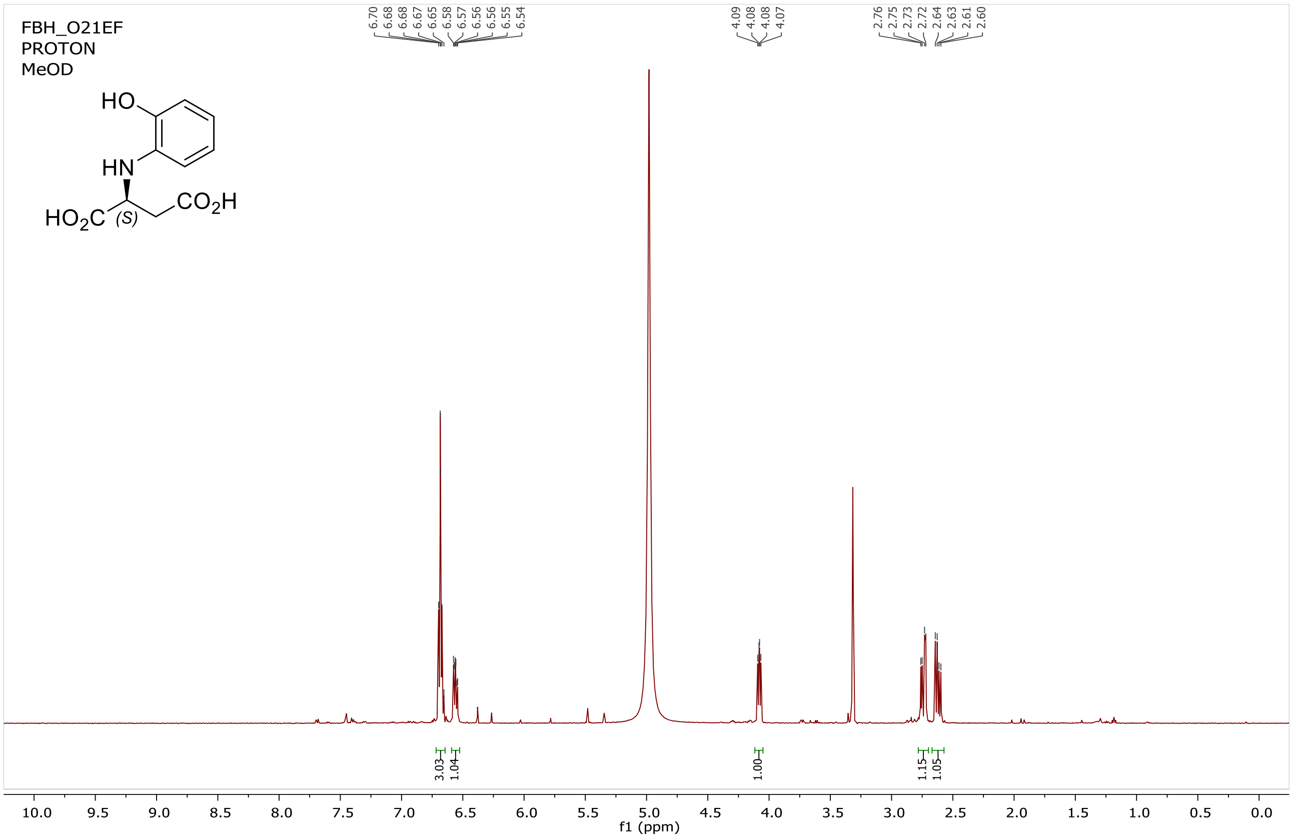


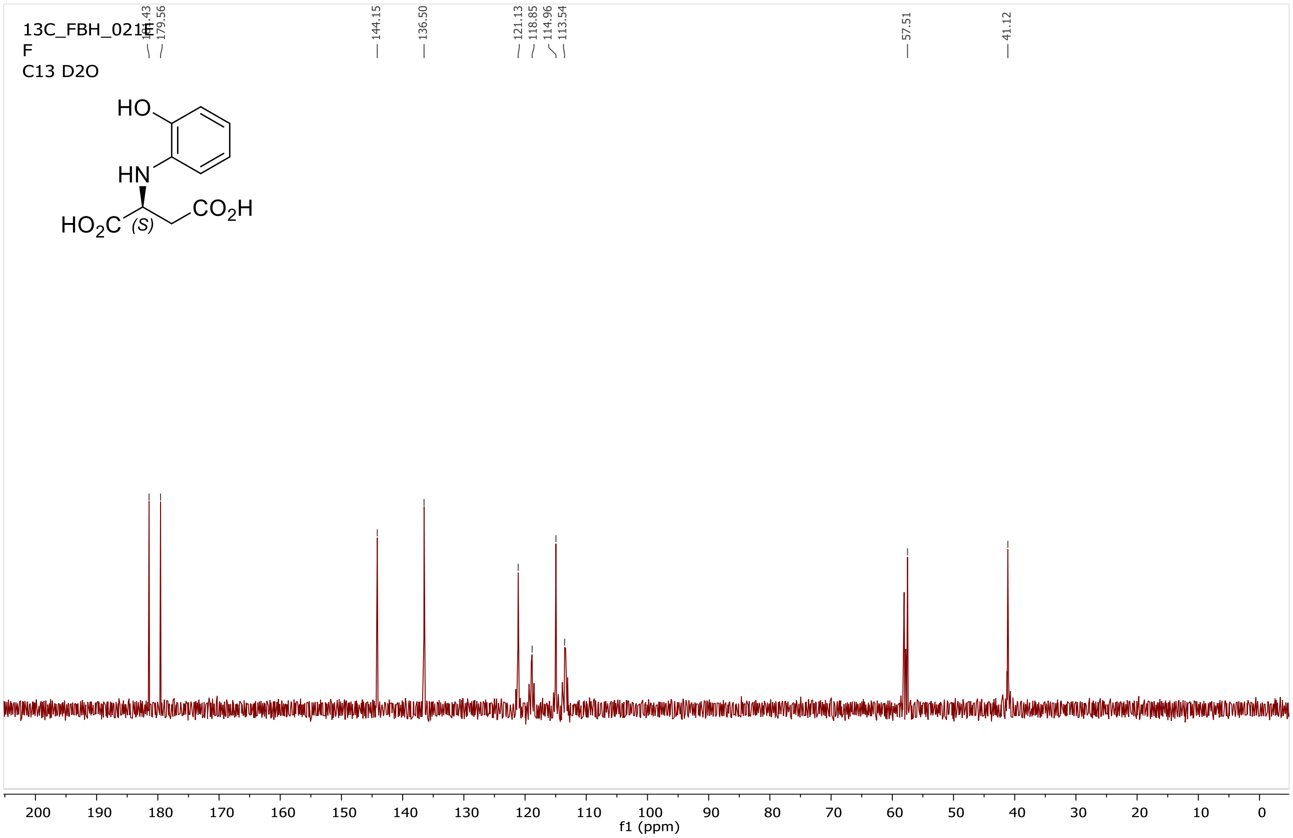


**Figure S1**: $^1$H NMR (top) and $^{13}$C NMR (bottom)of *(S)*-N-(2-hydroxyphenyl)aspartic acid (**3a**)

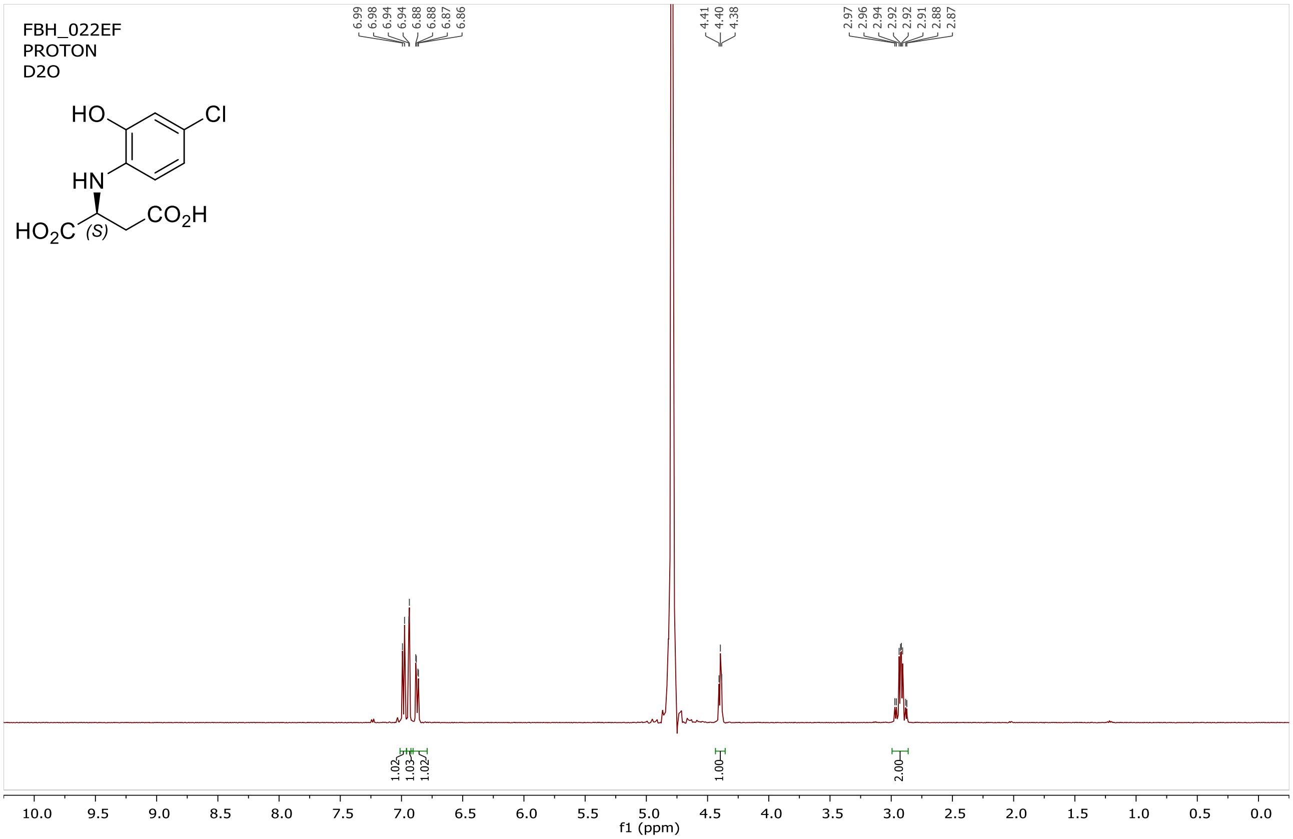


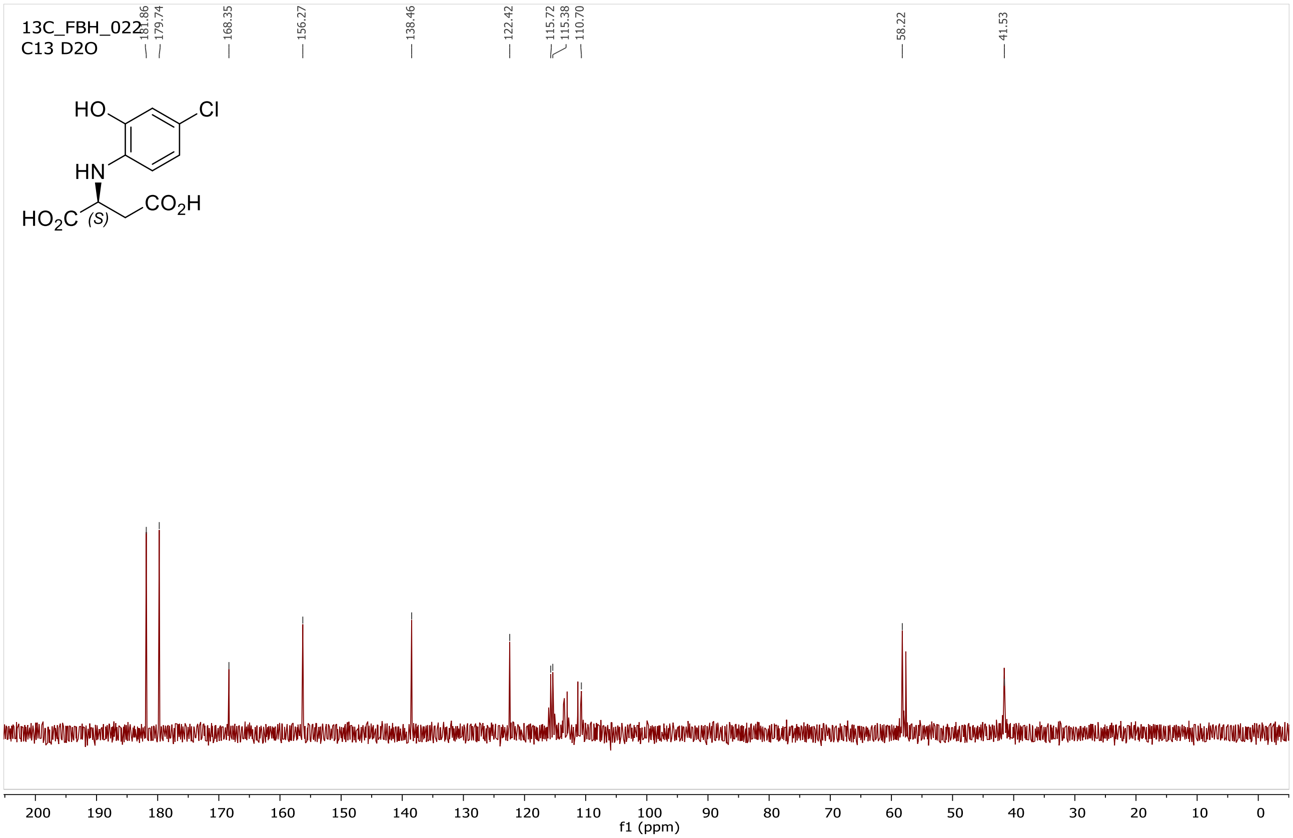


**Figure S2:**$^{1}$H NMR (top) and $^{13}$C NMR (bottom)of *(S)*-N-(4-chloro-2-hydroxyphenyl)aspartic acid(**3b**)

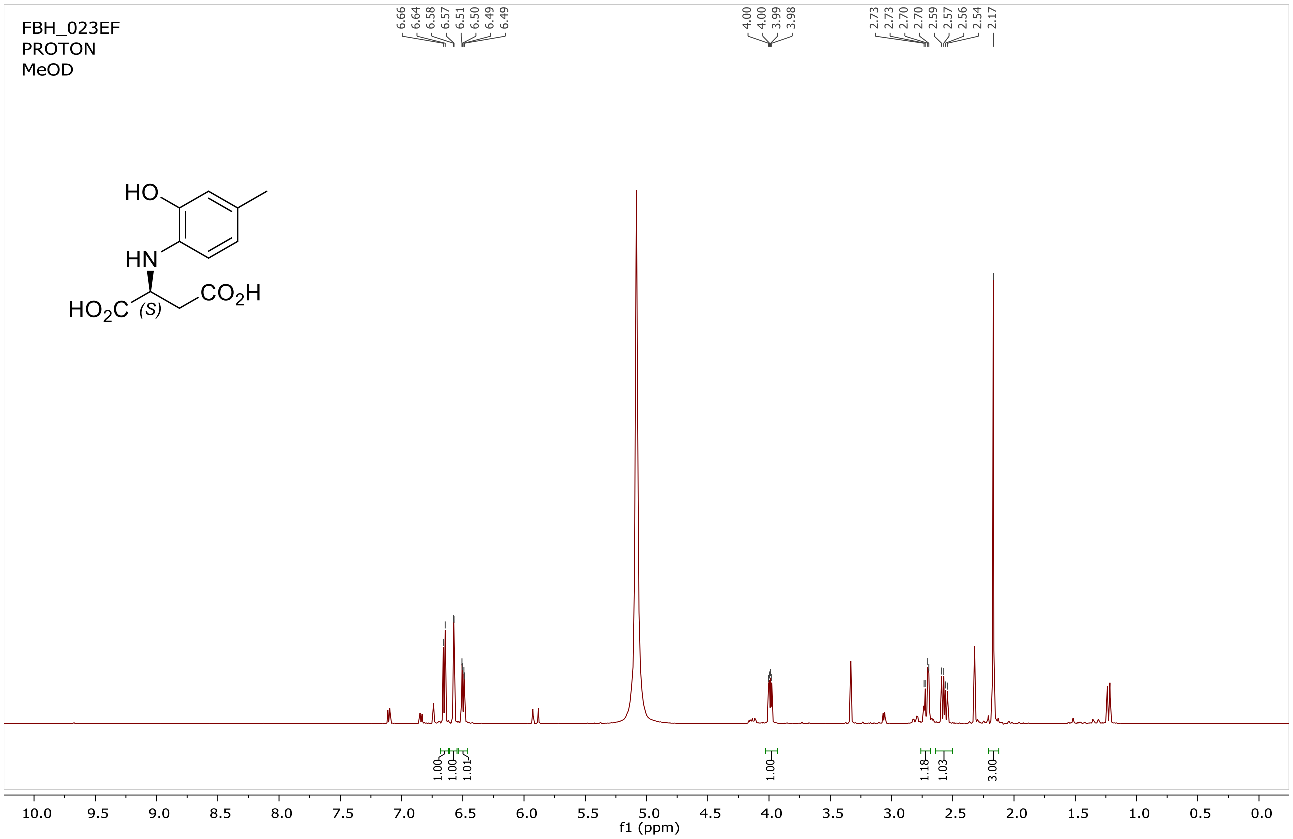


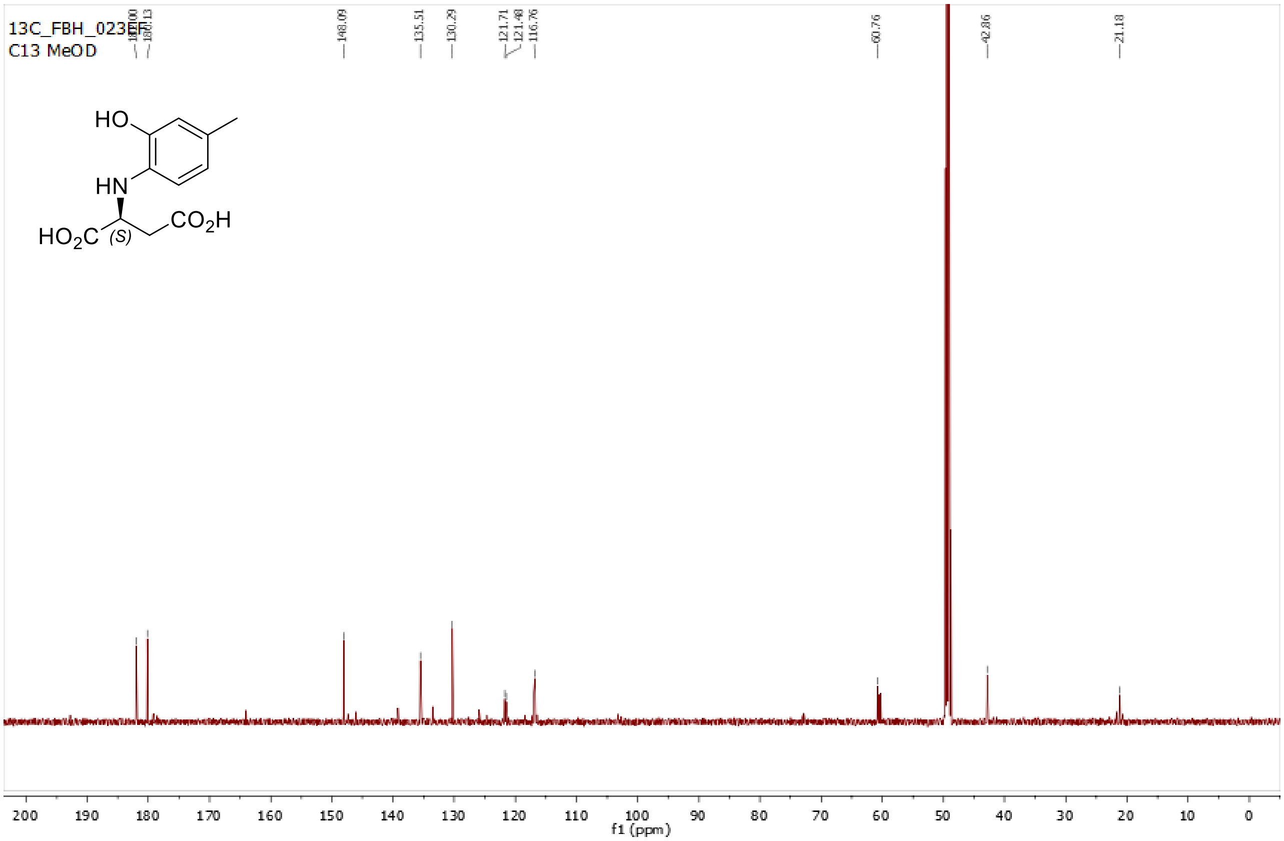


**Figure S3:**$^{1}$H NMR (top) and $^{13}$C NMR (bottom)of *(S)*-N-(2-hydroxy-4-methylphenyl)aspartic acid (**3c**)

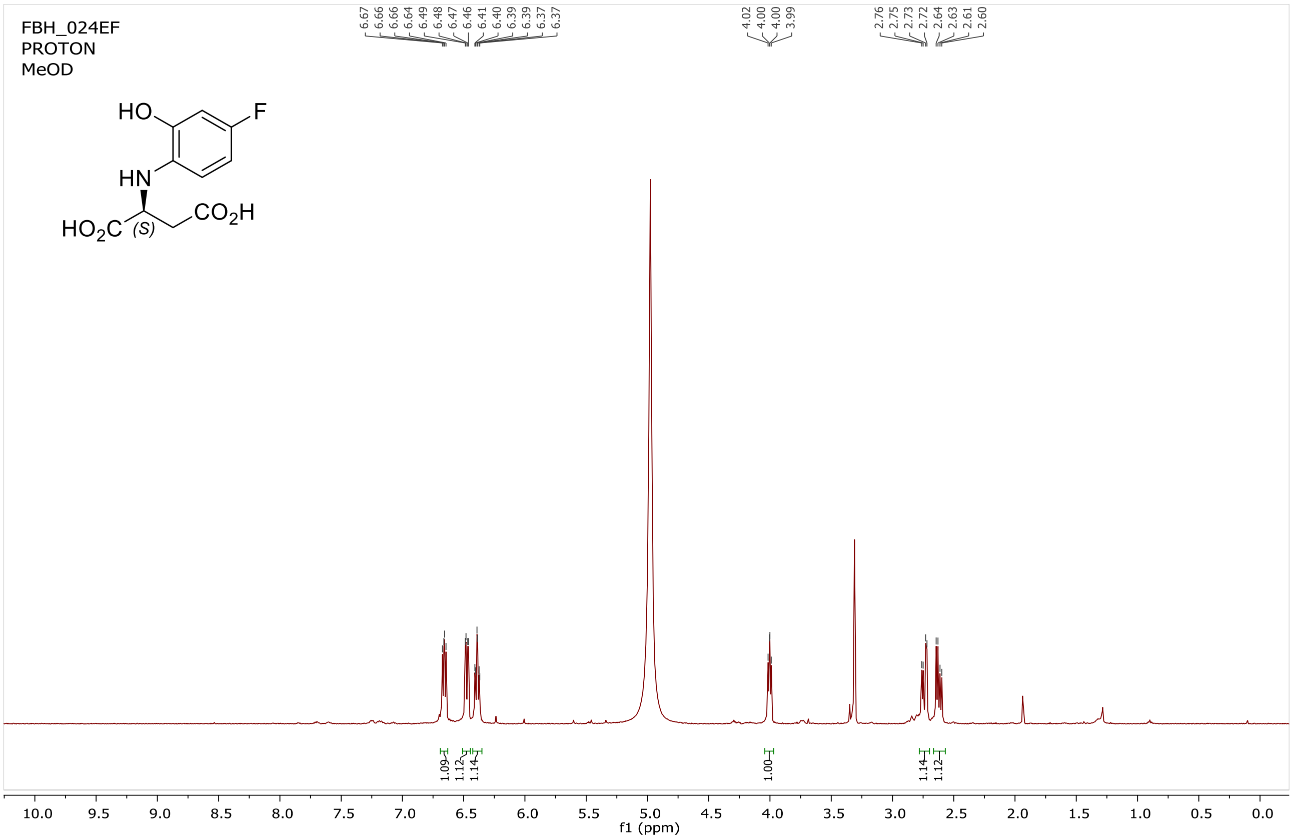


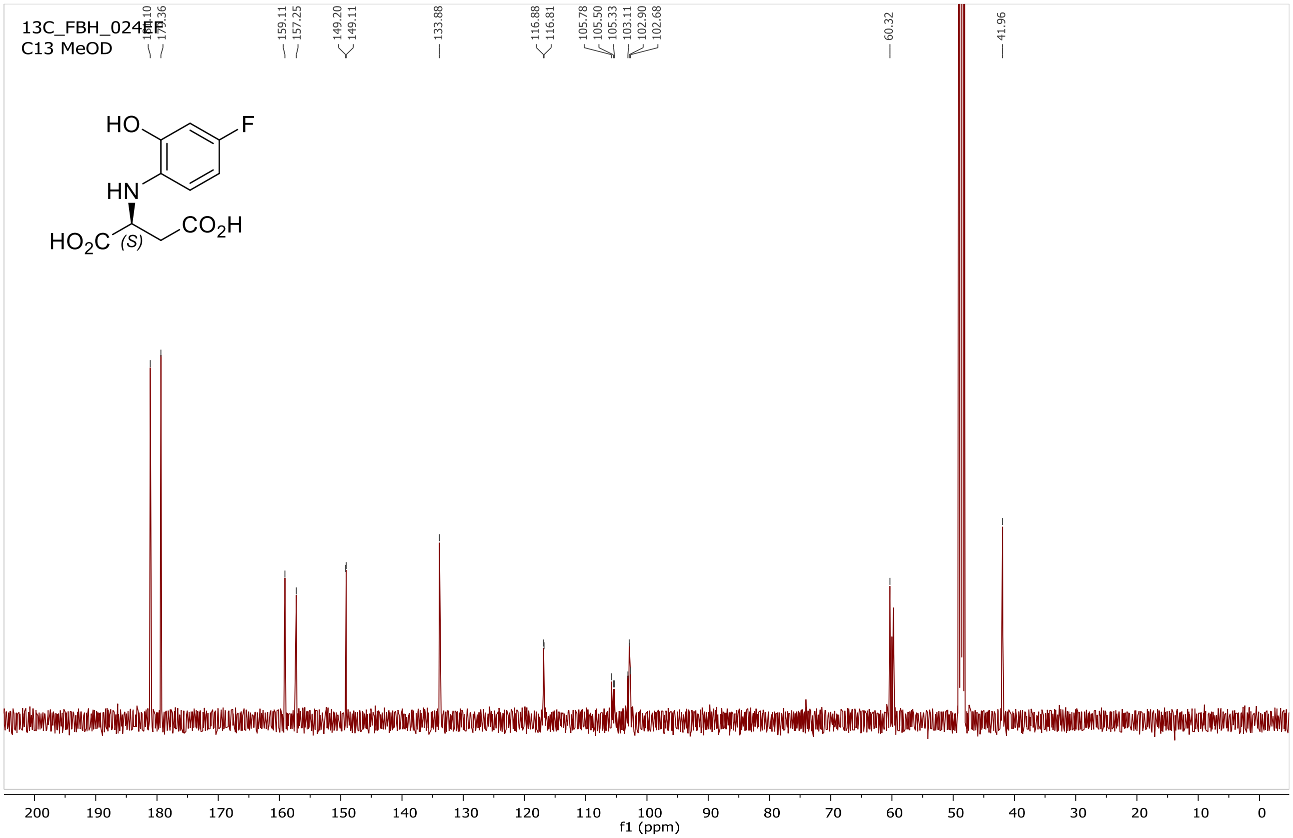


**Figure S4:**$^{1}$H NMR (top) and $^{13}$C NMR (bottom)of *(S)*-N-(4-fluoro-2-hydroxyphenyl)aspartic acid (**3d**)

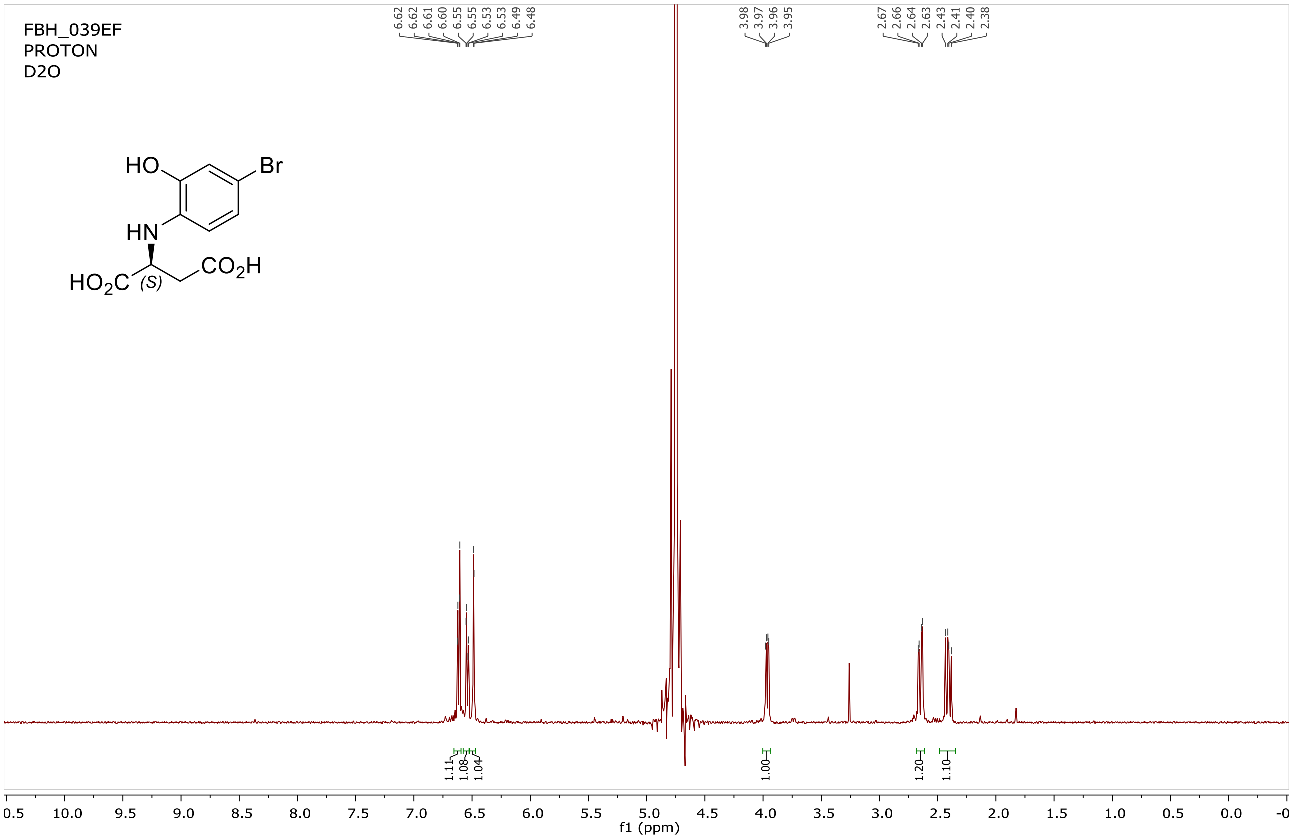


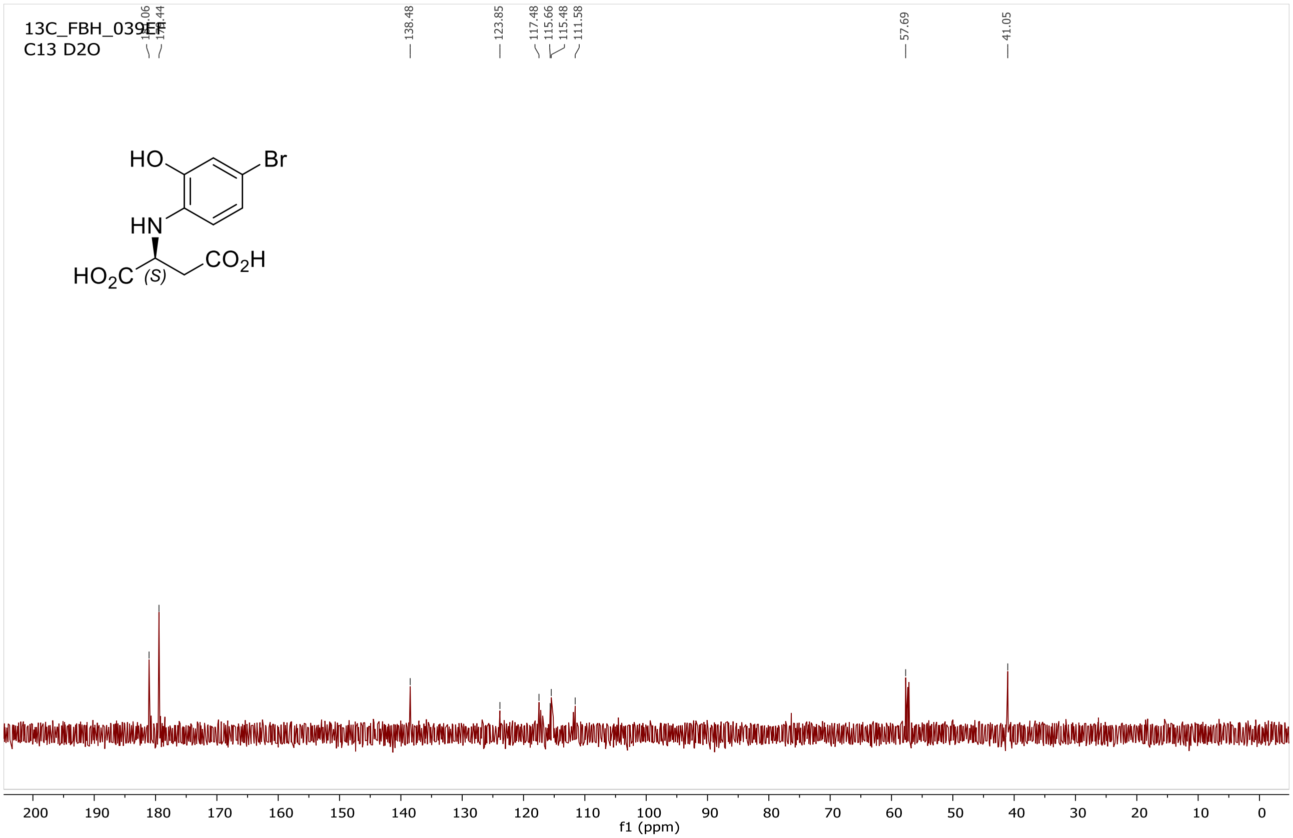


**Figure S5**: $^{1}$H NMR (top) and $^{13}$C NMR (bottom) of *(S)*-N-(4-bromo-2-hydroxyphenyl)aspartic acid (**3e**)

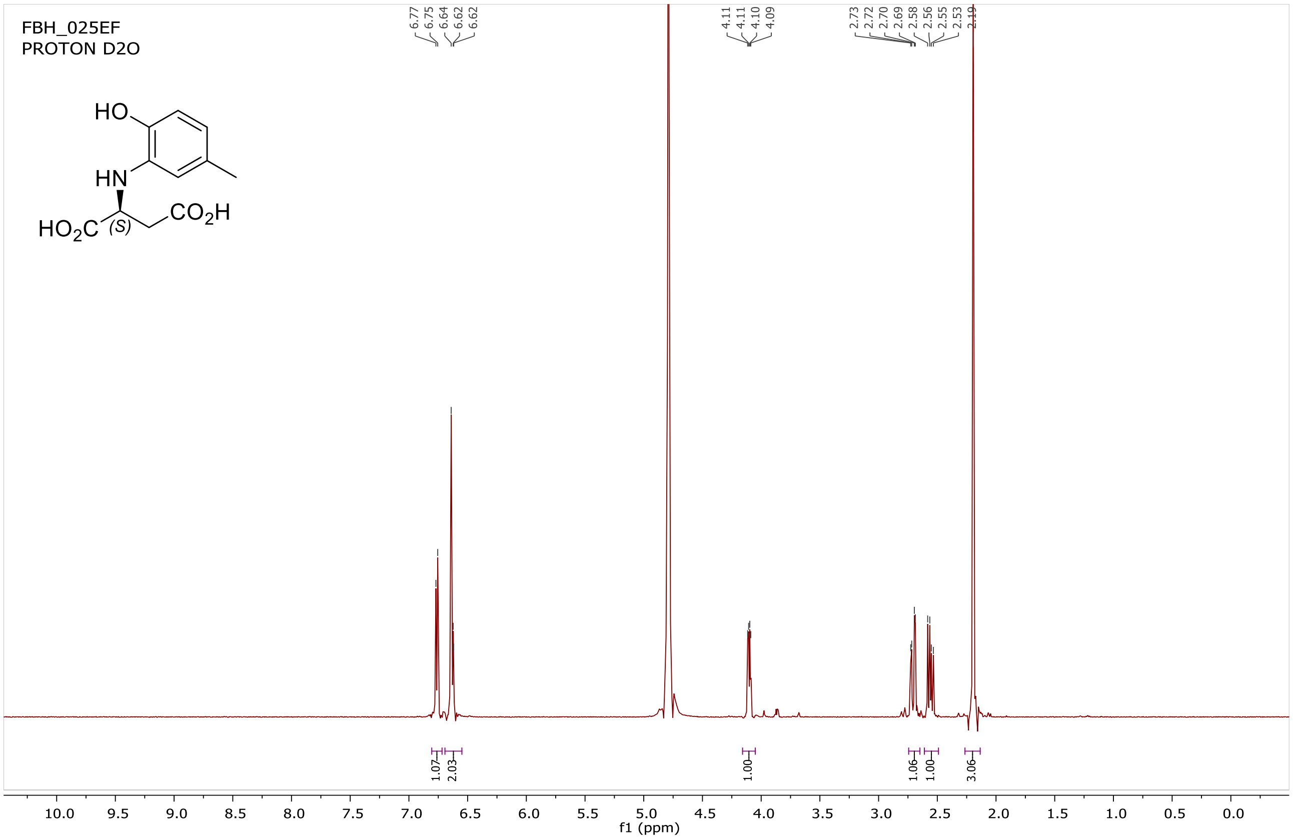


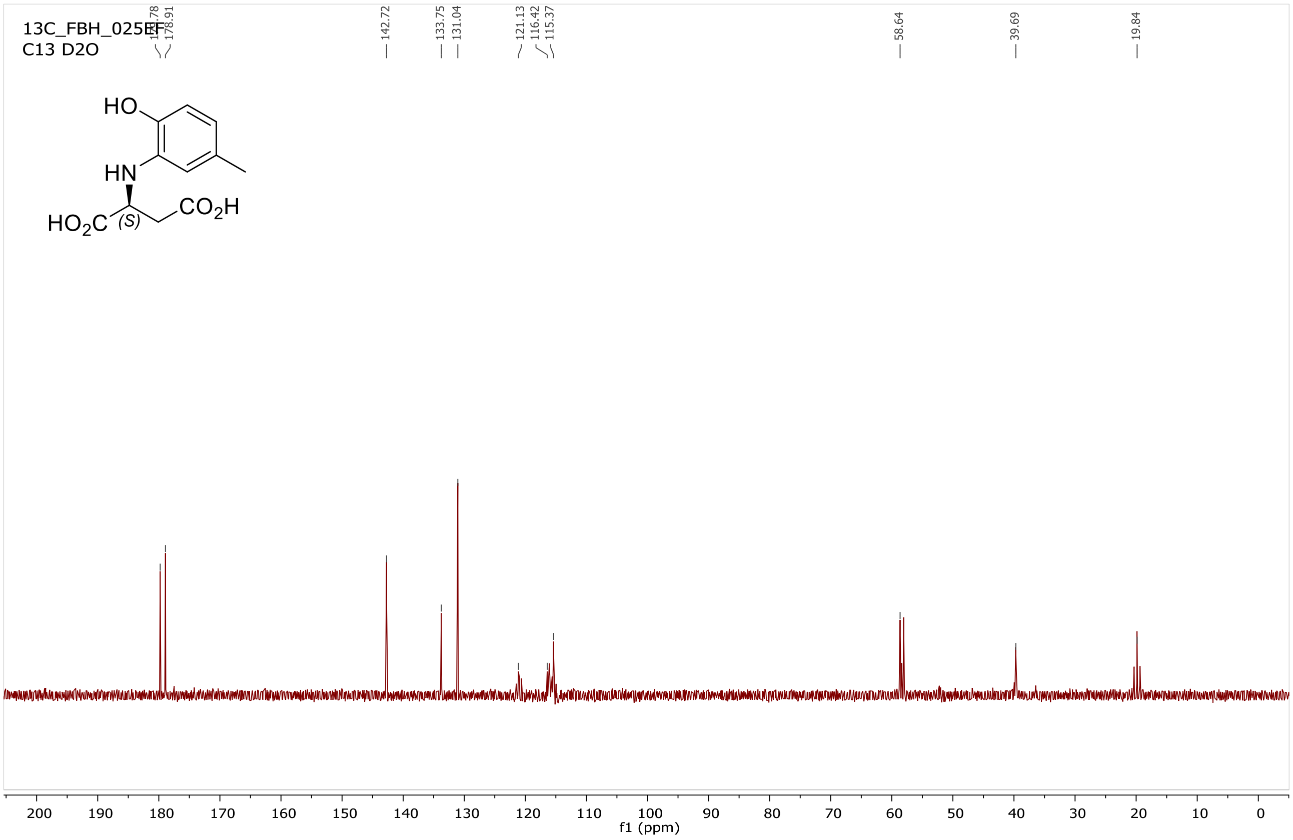


**Figure S6:** $^{1}H$ NMR (top) and $^{13}C$ NMR (bottom)of *(S)*-N-(2-hydroxy-5-methylphenyl)aspartic acid (**3f**)

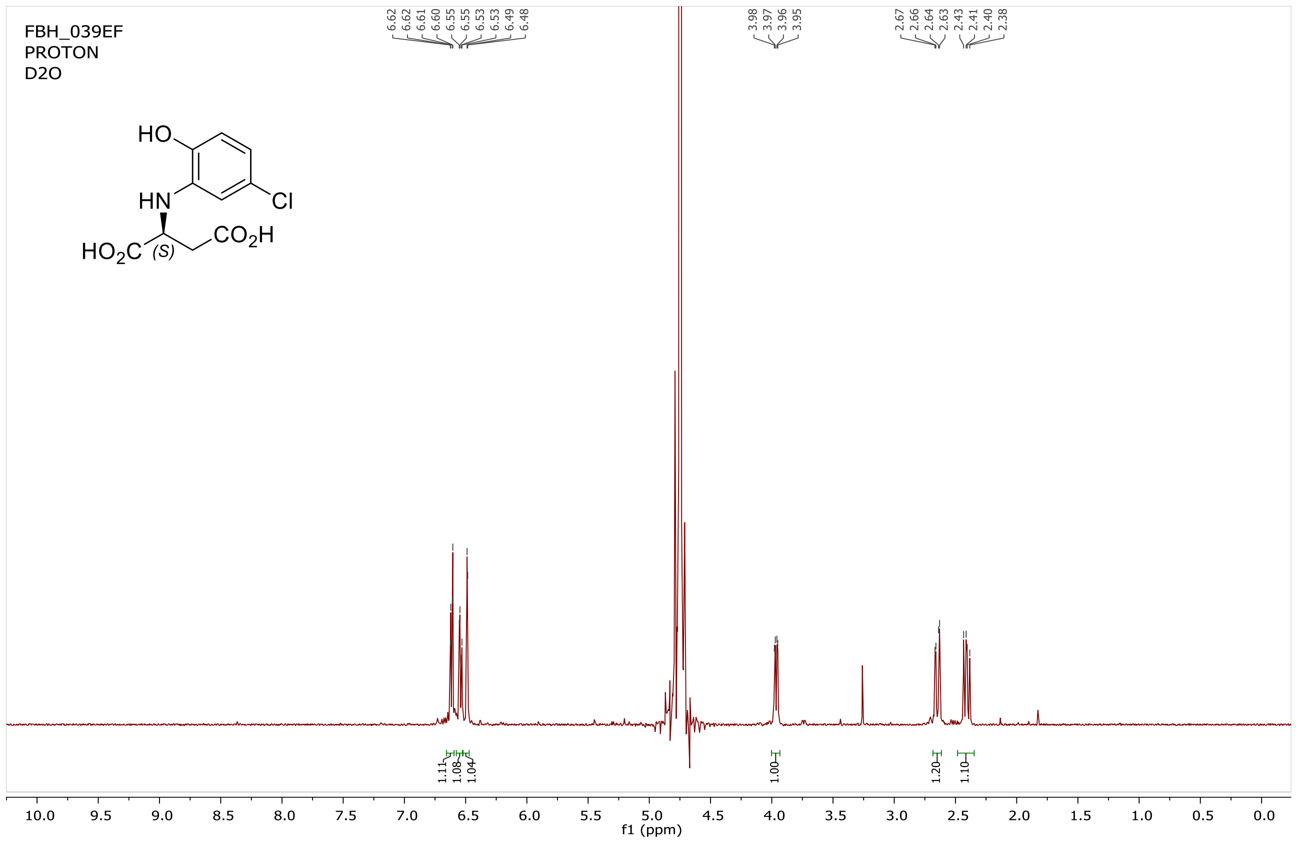


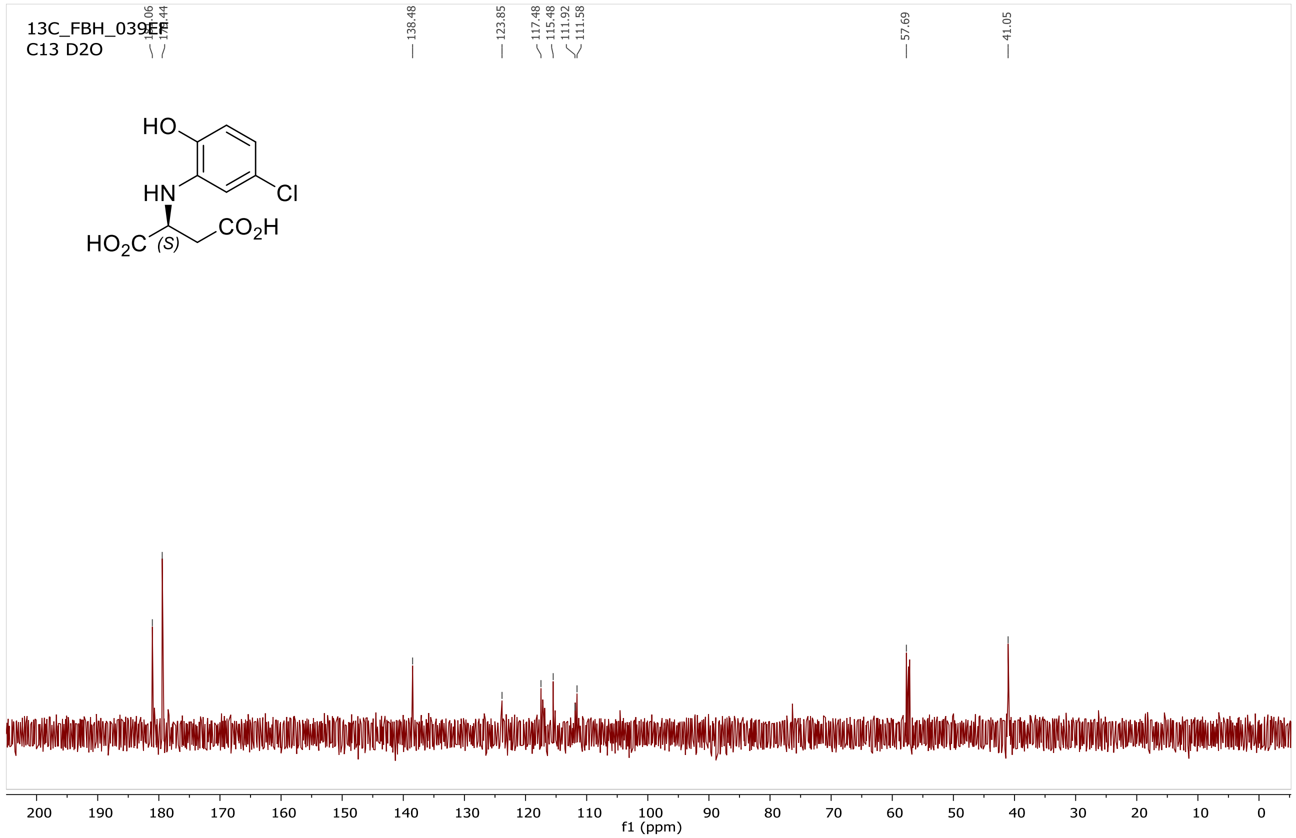


**Figure S7:**$^{1}$H NMR (top) and $^{13}$C NMR (bottom) of *(S)*-N-(5-chloro-2-hydroxyphenyl)aspartic acid (**3g**).

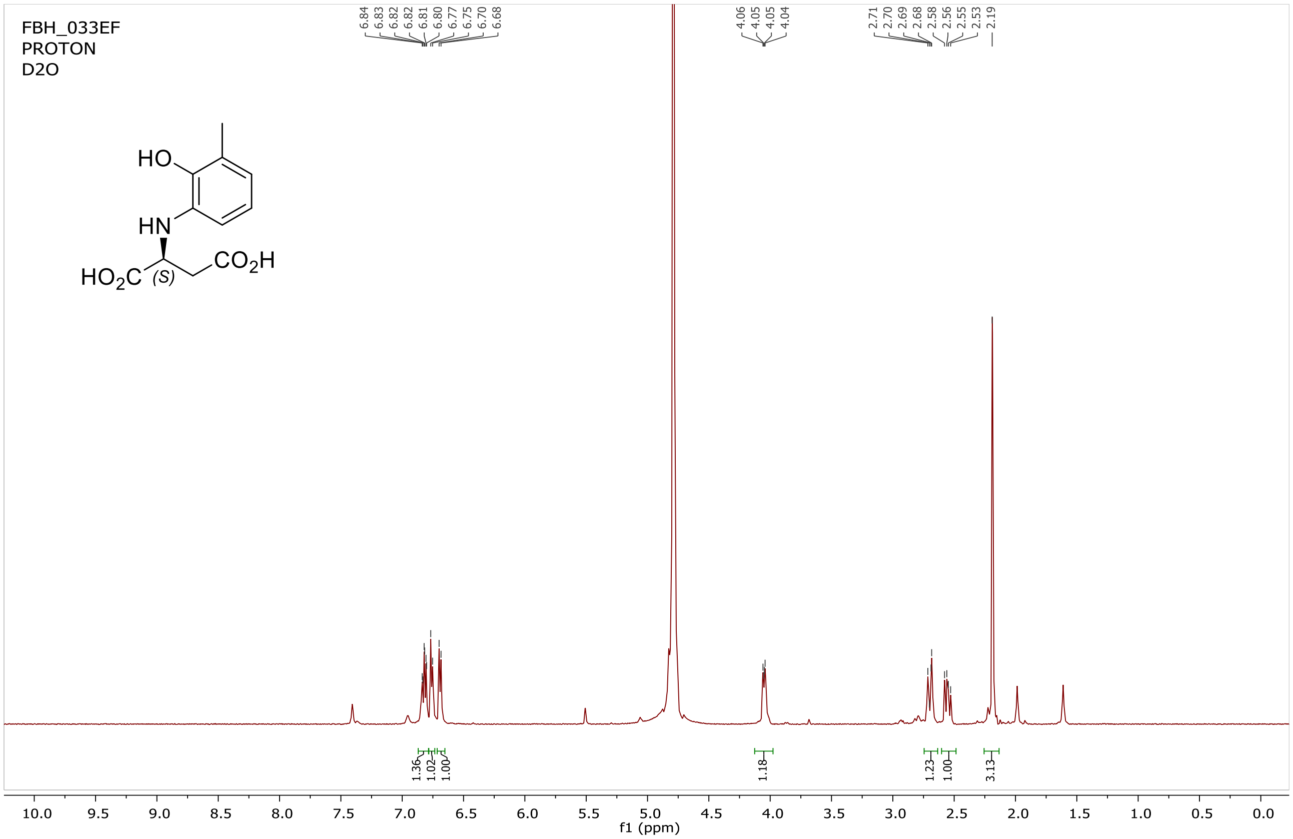


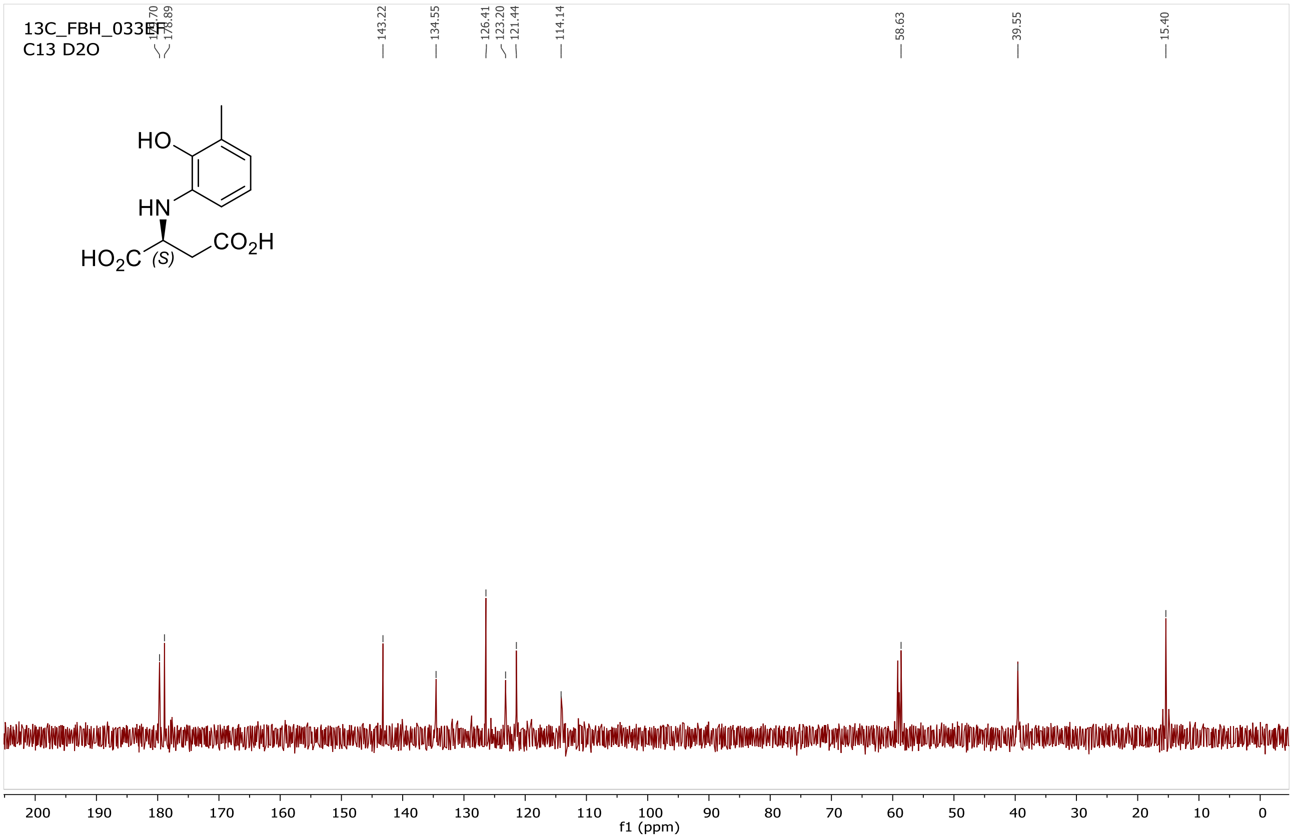


**Figure S8:** $^{1}$H NMR (top) and $^{13}$C NMR (bottom) of *(S)*-N-(2-hydroxy-3-methylphenyl)aspartic acid (**3h**)

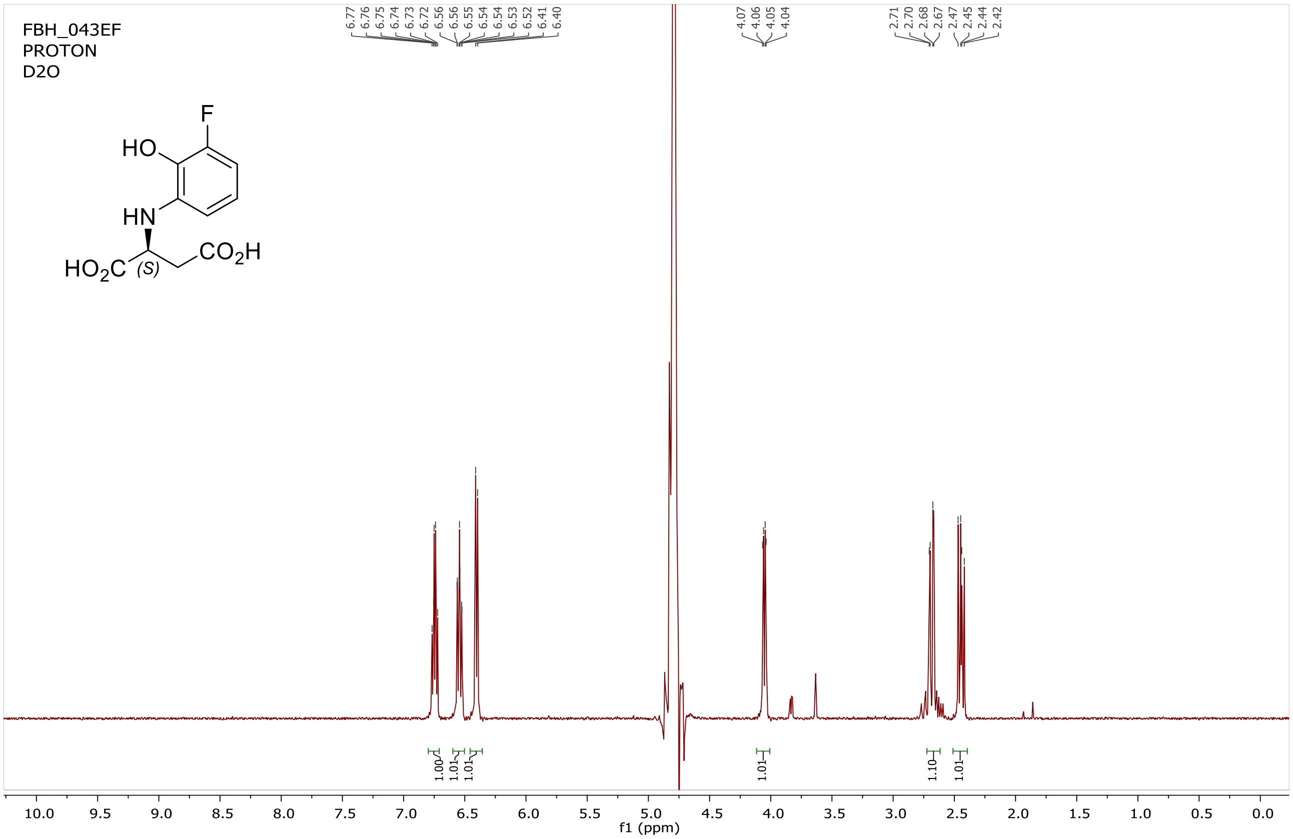


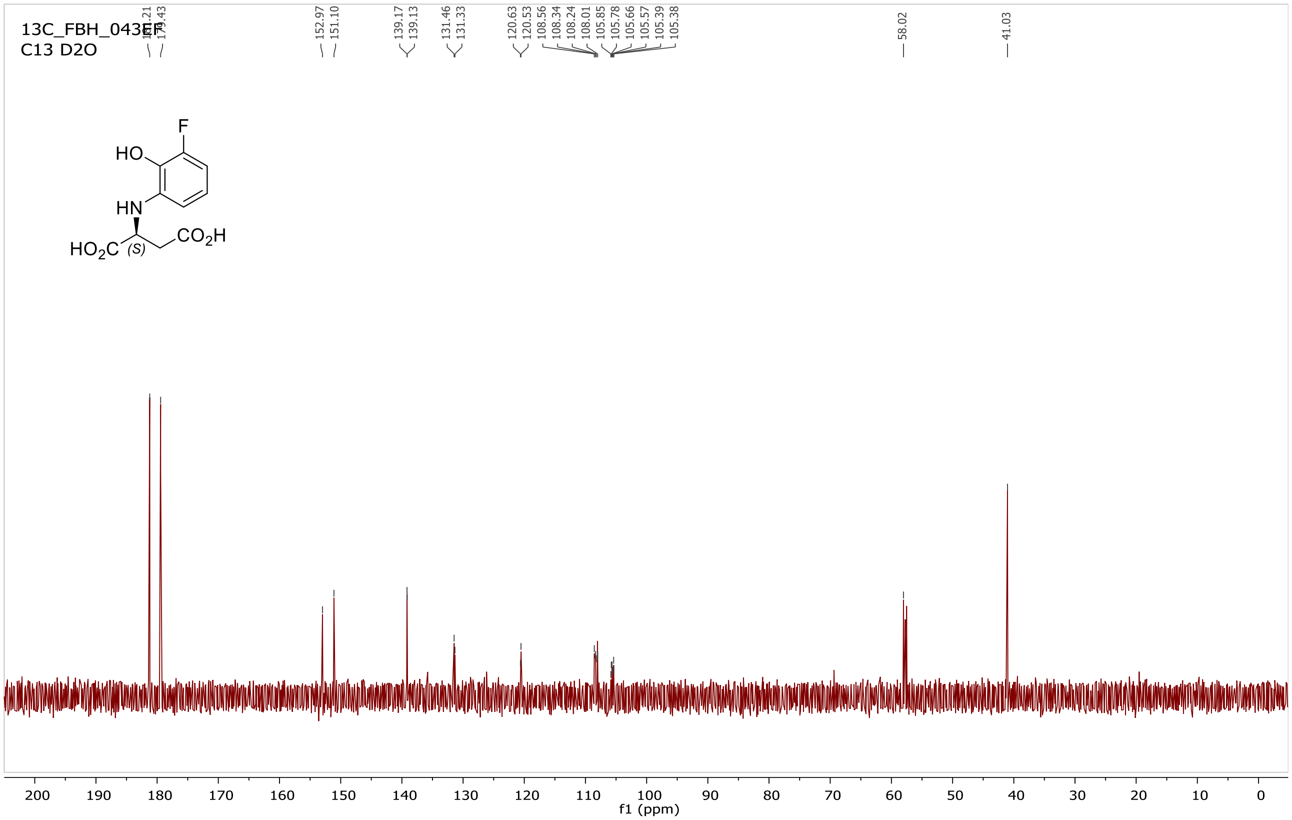


**Figure S9:** $^1$H NMR (top) and $^{13}$C NMR (bottom) of (*S*)-N-(3-fluoro-2-hydroxyphenyl) aspartic acid (**3i**)

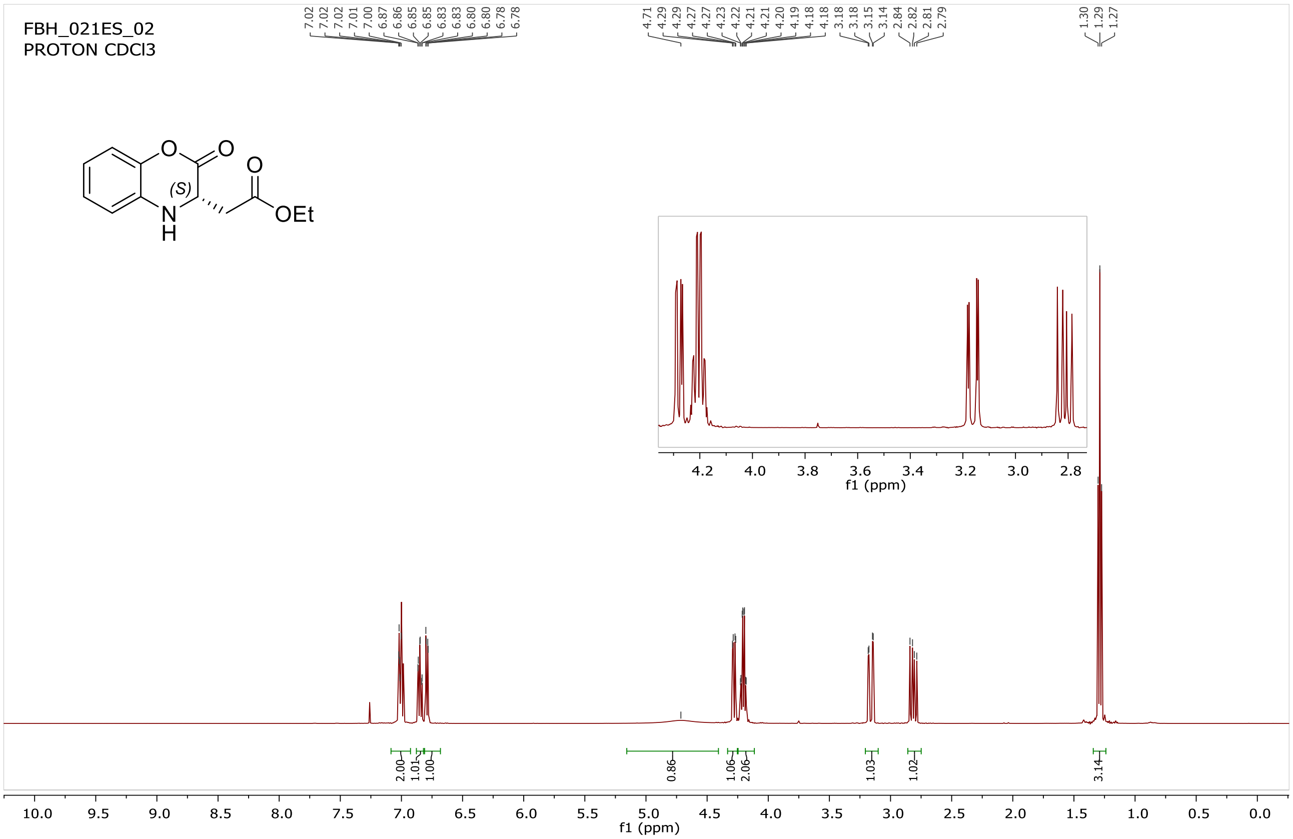


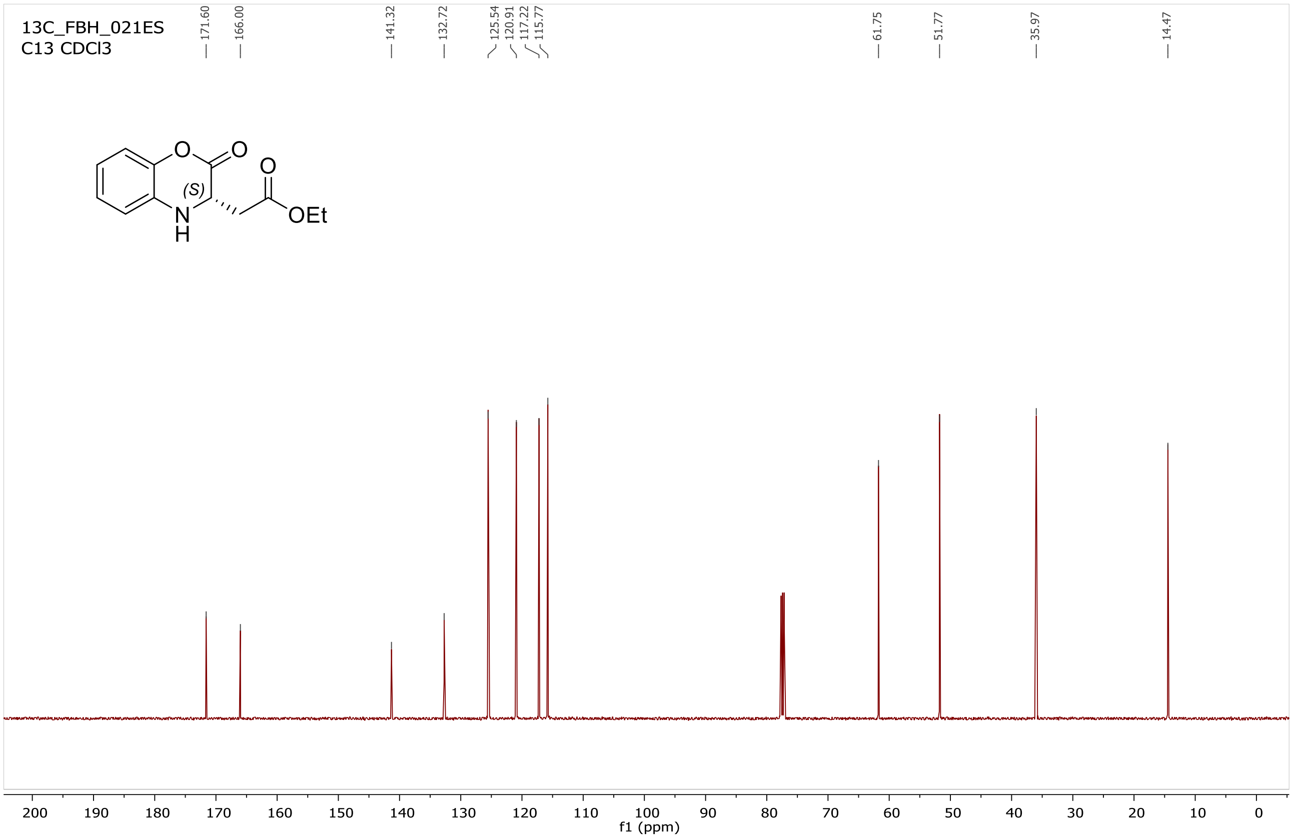


**Figure S10:**$^{1}$H NMR (top) and $^{13}$C NMR (bottom) of *(S)*-Ethyl 2-(2-oxo-3,4-dihydro-2H-benzo[b][1,4]oxazin-3-yl)acetate (**4a**)

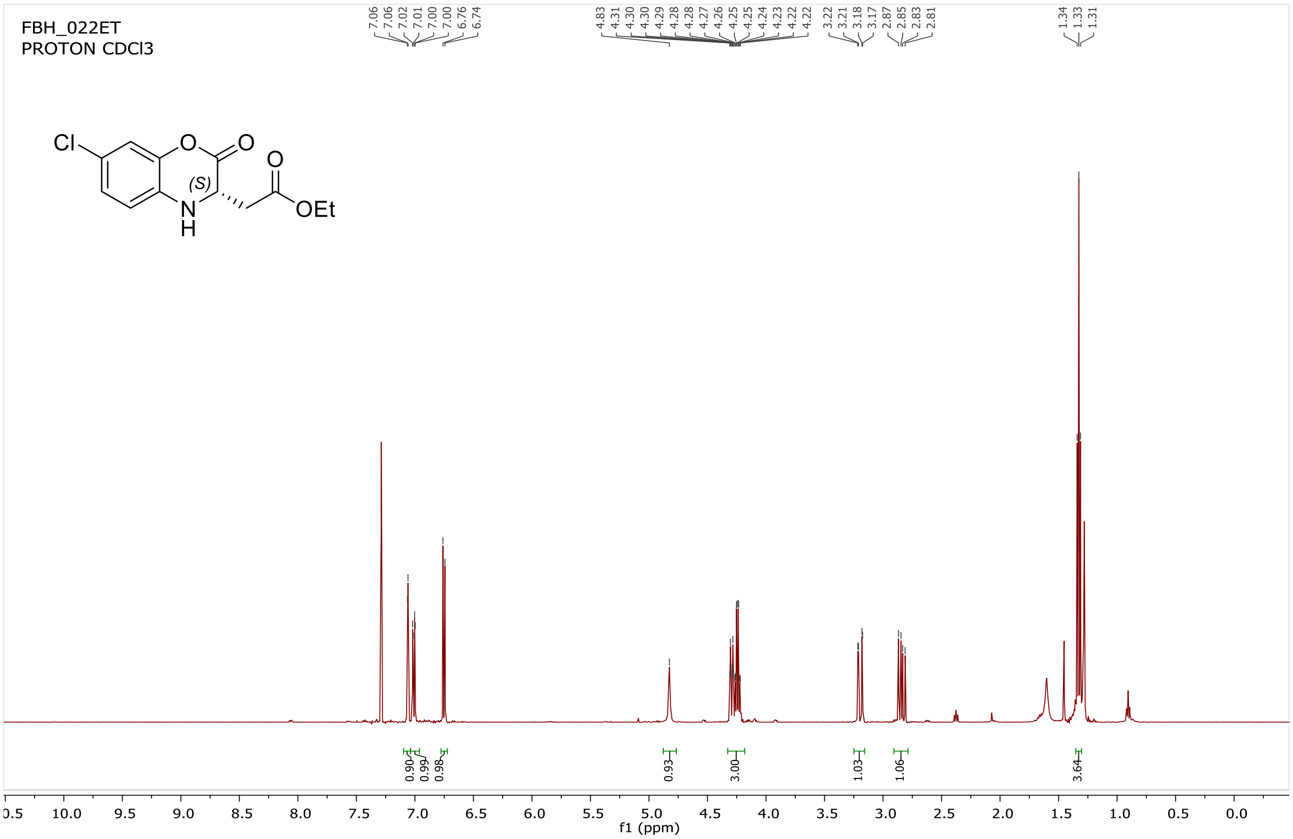


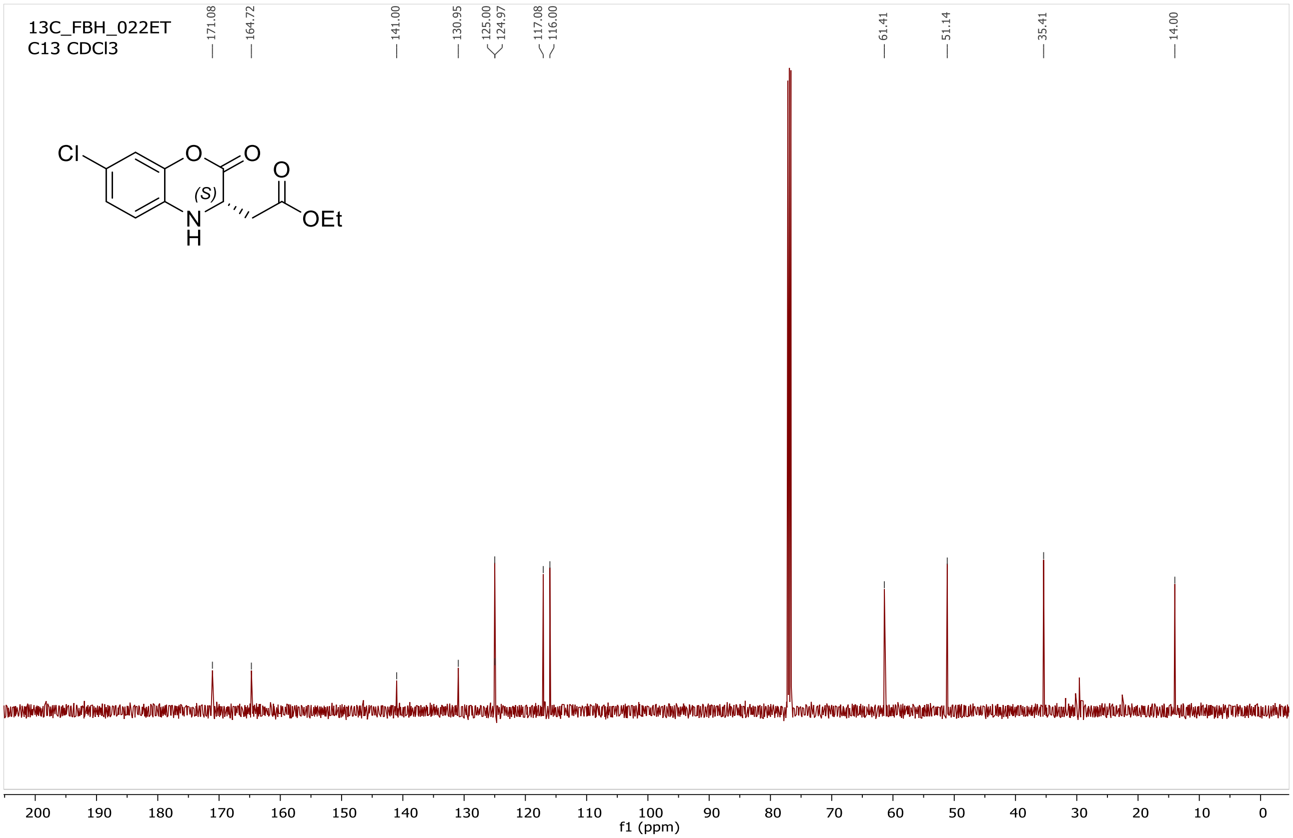


**Figure S11:** $^{1}$H NMR (top) and $^{13}$C NMR (bottom) of *(S)*-Ethyl 2-(7-chloro-2-oxo-3,4-dihydro-2H-benzo[b][1,4]oxazin-3-yl)acetate (**4b**)

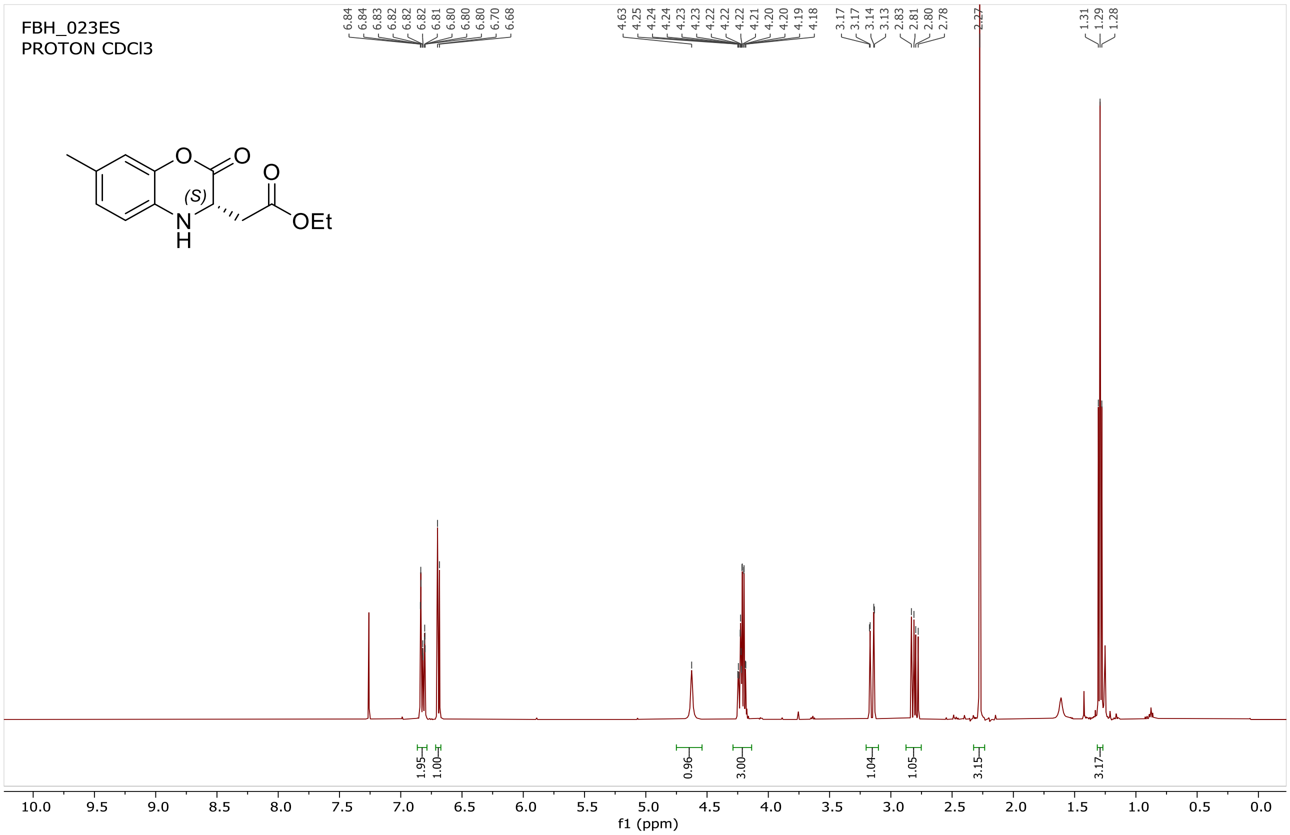


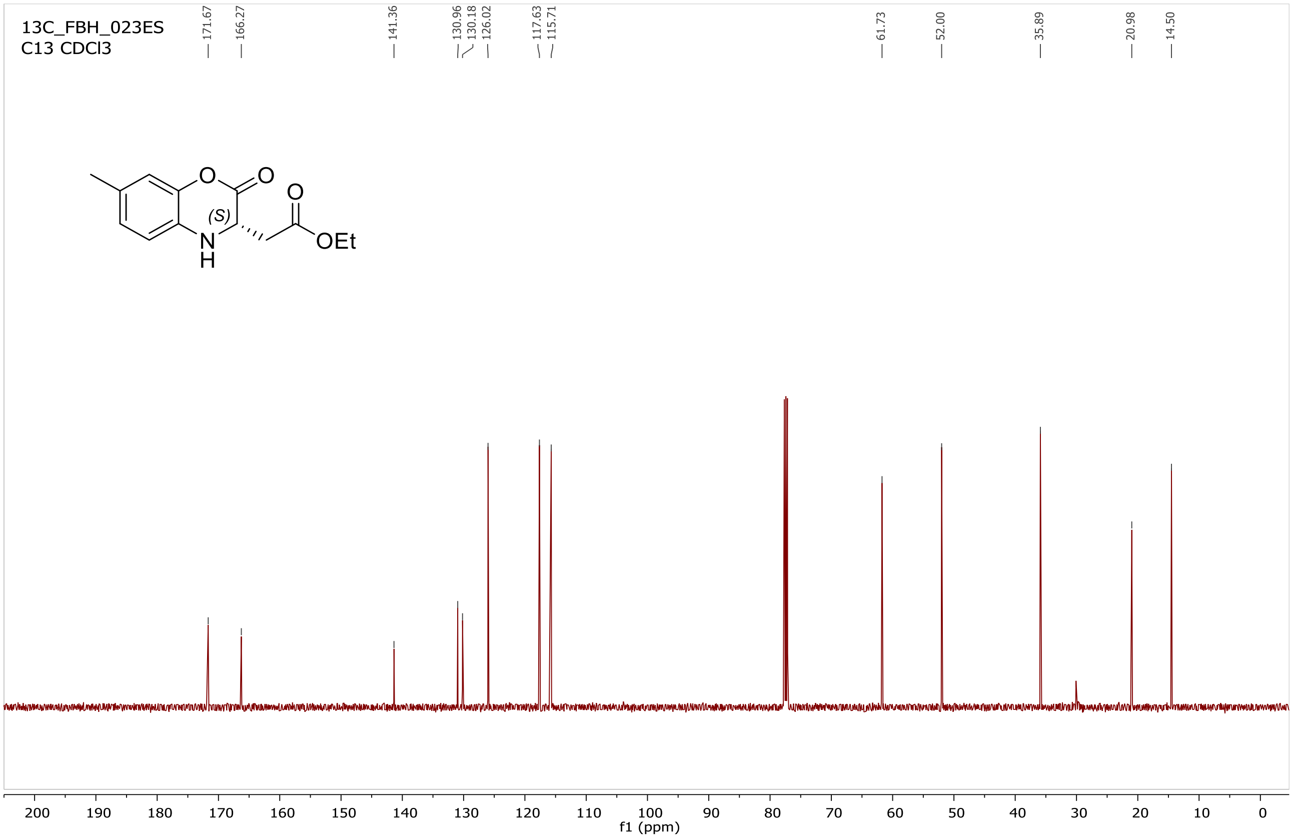


**Figure S12:**$^{1}$H NMR (top) and $^{13}$C NMR (bottom) of *(S)*-Ethyl 2-(7-methyl-2-oxo-3,4-dihydro-2H-benzo[b][1,4]oxazin-3-yl)acetate (**4c**)

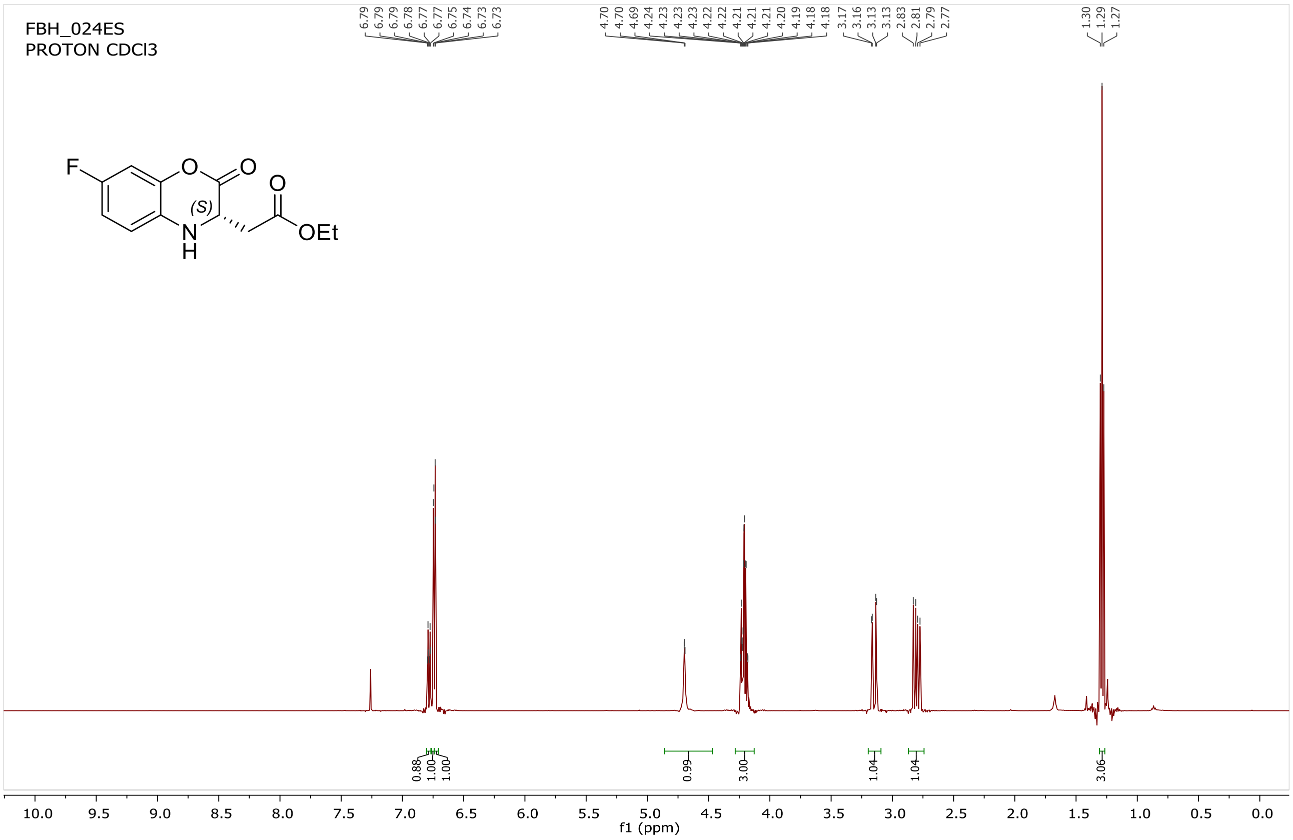


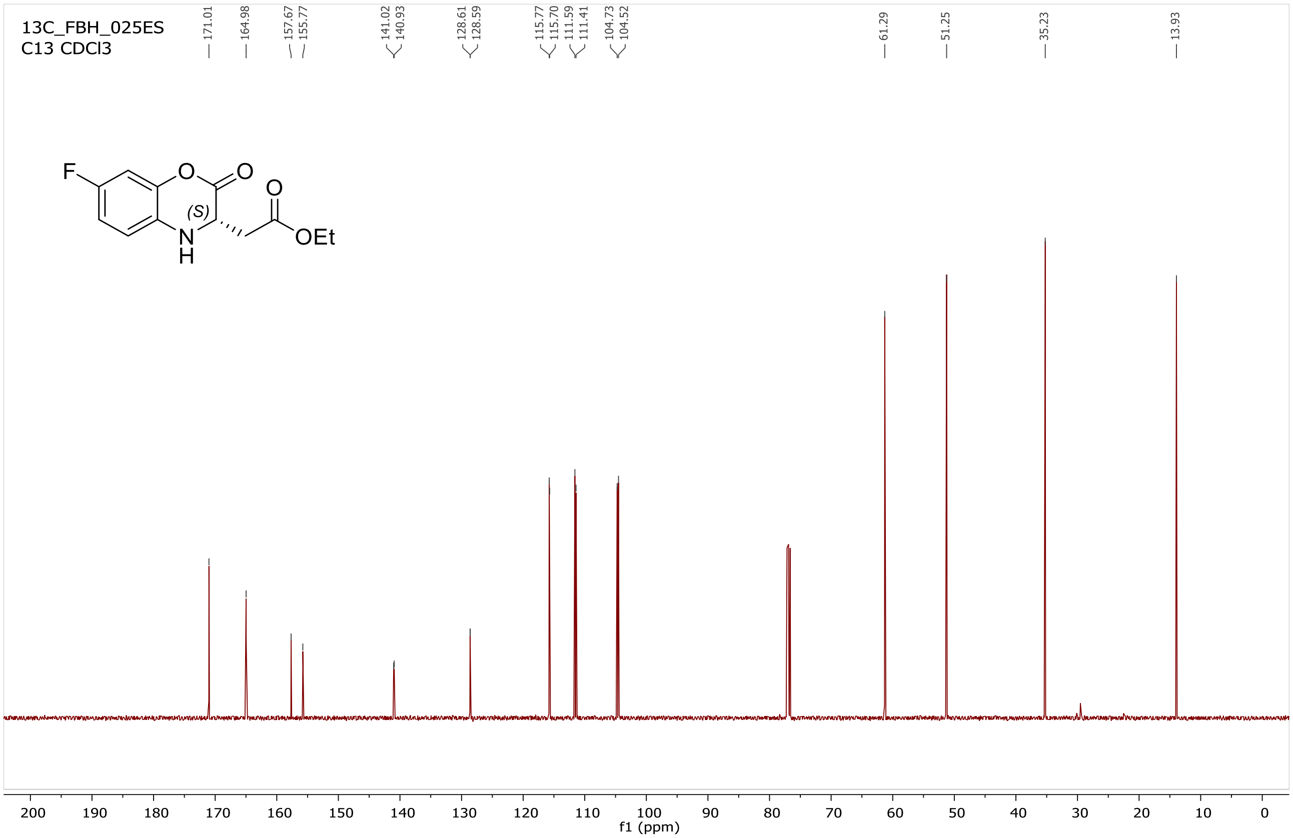


**Figure S13:**$^{1}$H NMR (top) and $^{13}$C NMR (bottom) of *(S)*-Ethyl 2-(7-fluoro-2-oxo-3,4-dihydro-2H-benzo[b][1,4]oxazin-3-yl)acetate (**4d**)

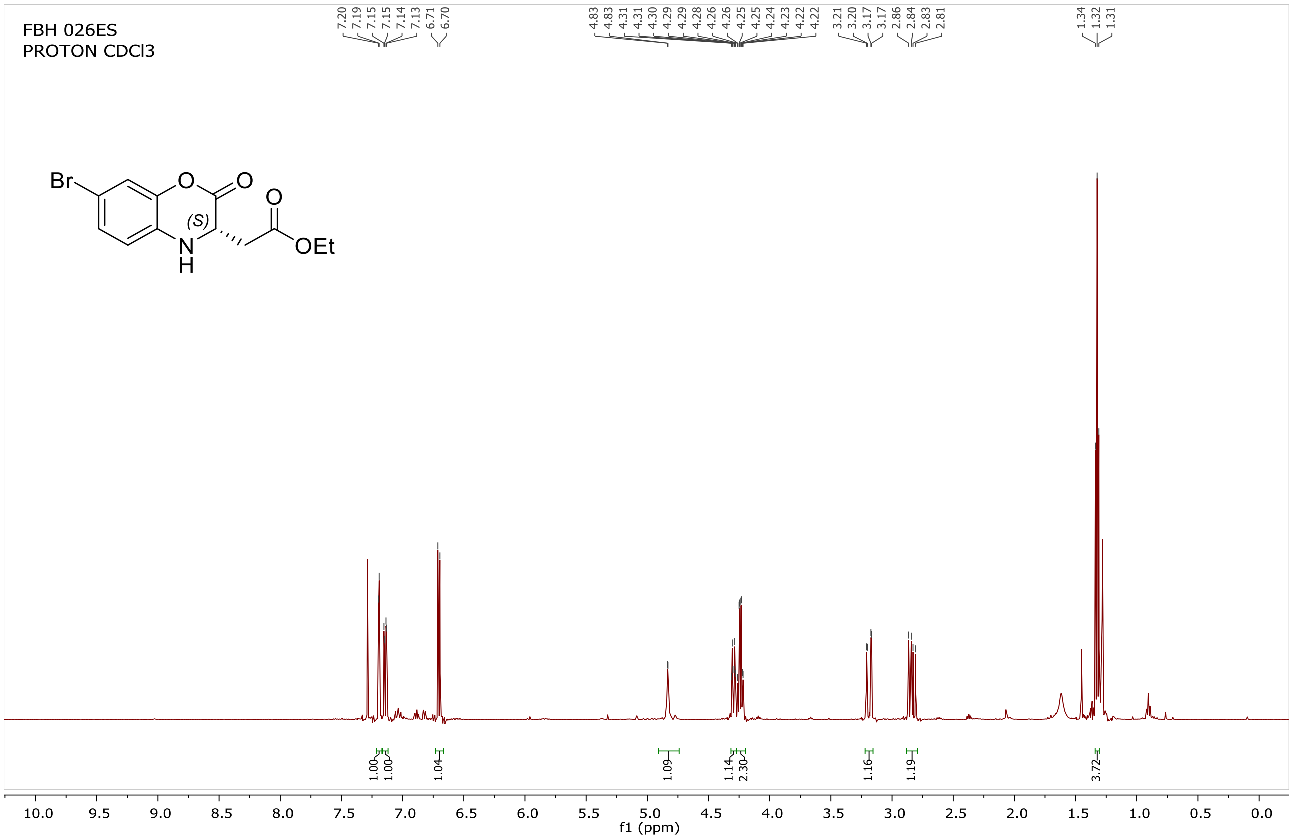


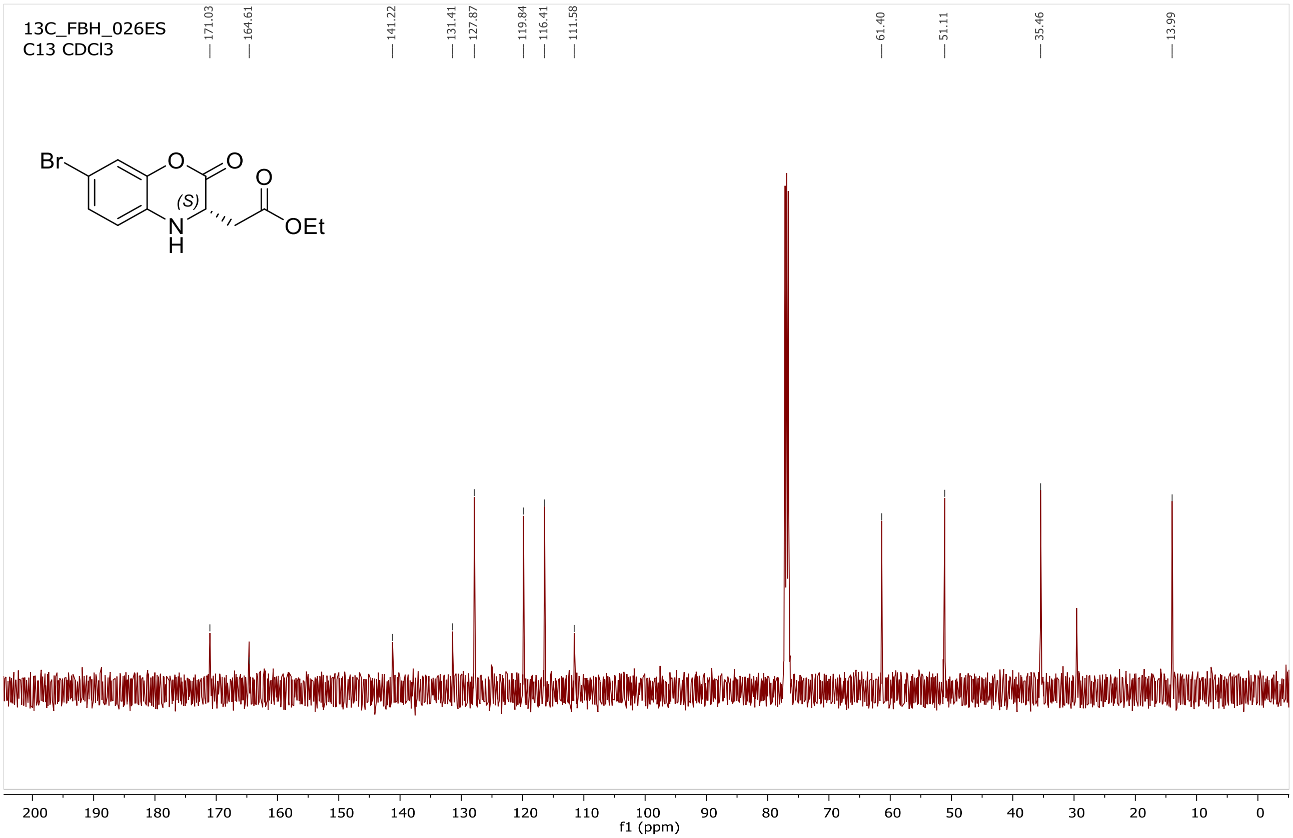


**Figure S14:**$^{1}$H NMR (top) and $^{13}$C NMR (bottom) of *(S)*-Ethyl 2-(7-bromo-2-oxo-3,4-dihydro-2H-benzo[b][1,4]oxazin-3-yl)acetate (**4e**)

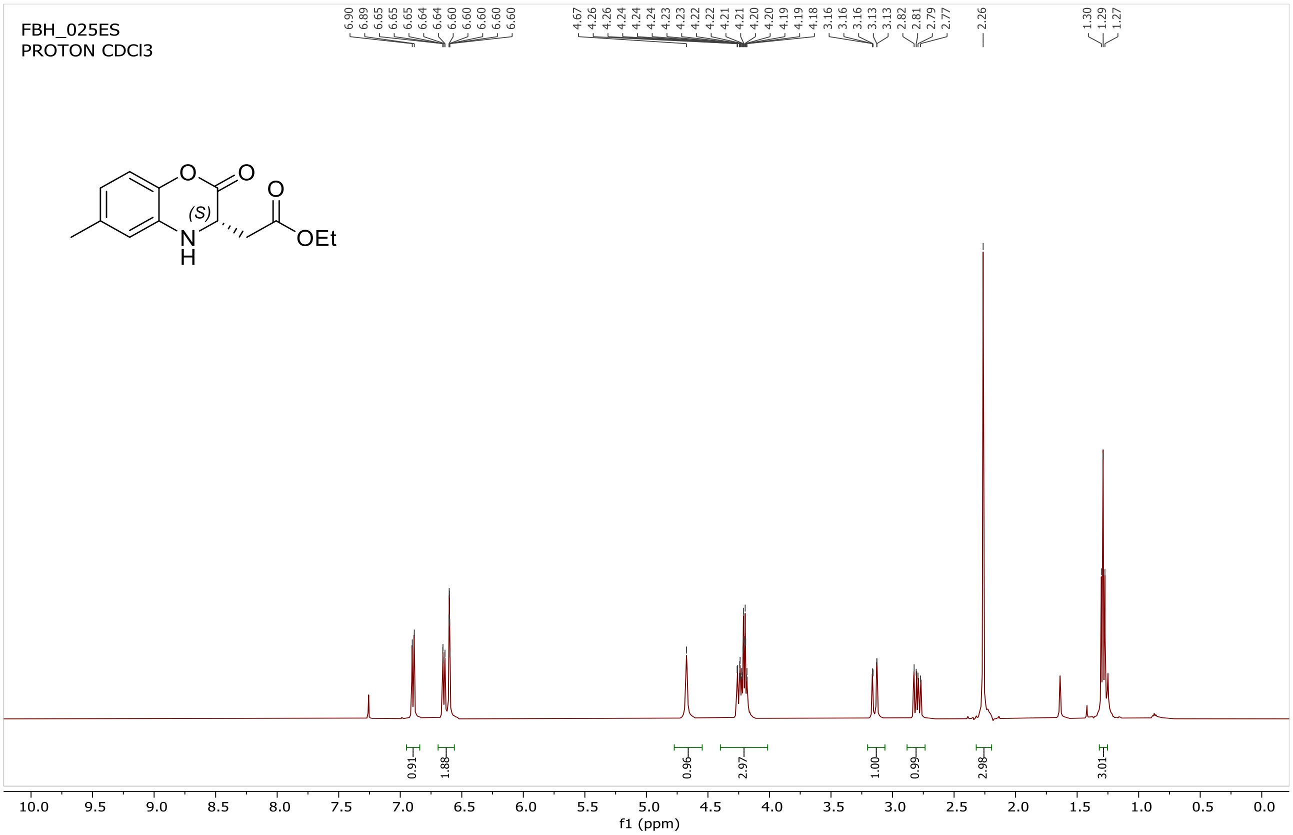


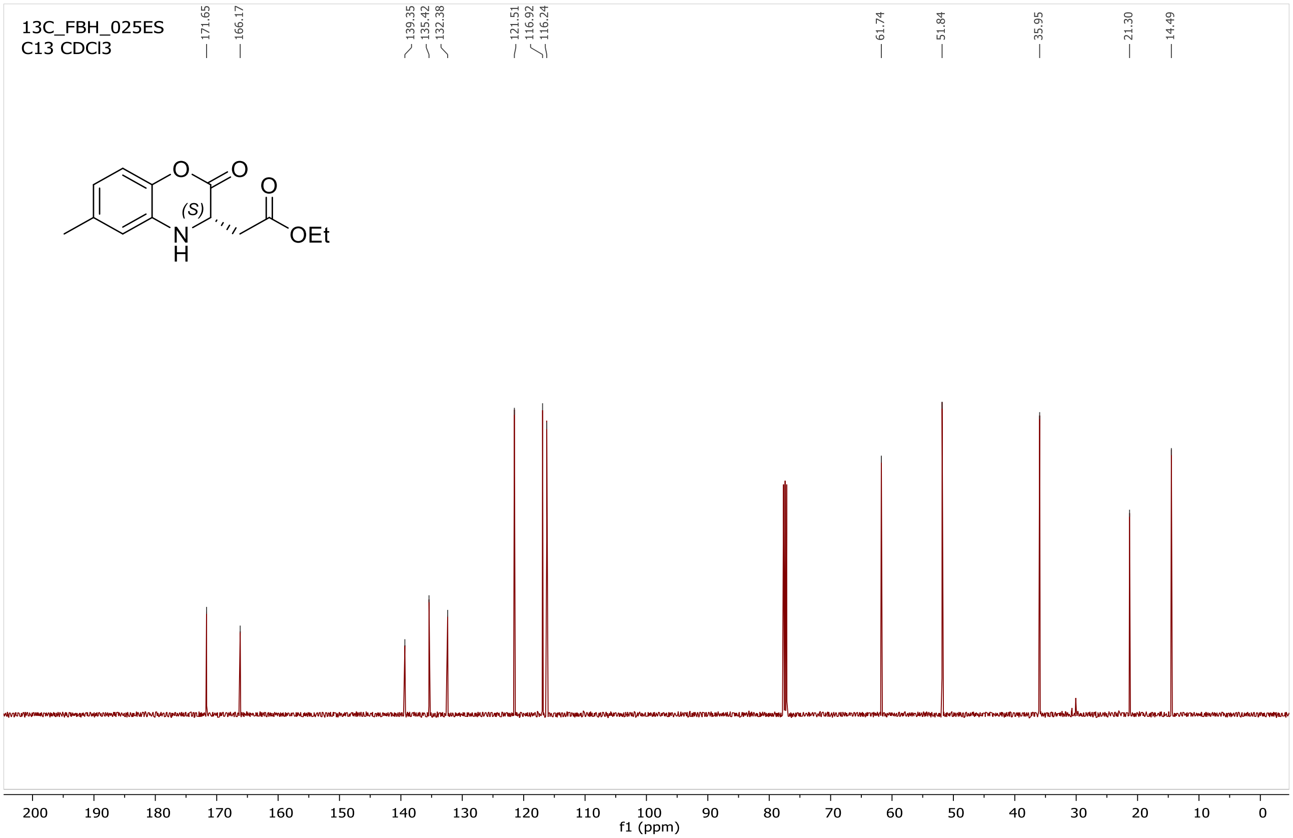


**Figure S15:**$^{1}$H NMR (top) and $^{13}$C NMR (bottom) of *(S)*-Ethyl 2-(6-methyl-2-oxo-3,4-dihydro-2H-benzo[b][1,4]oxazin-3-yl)acetate (**4f**)

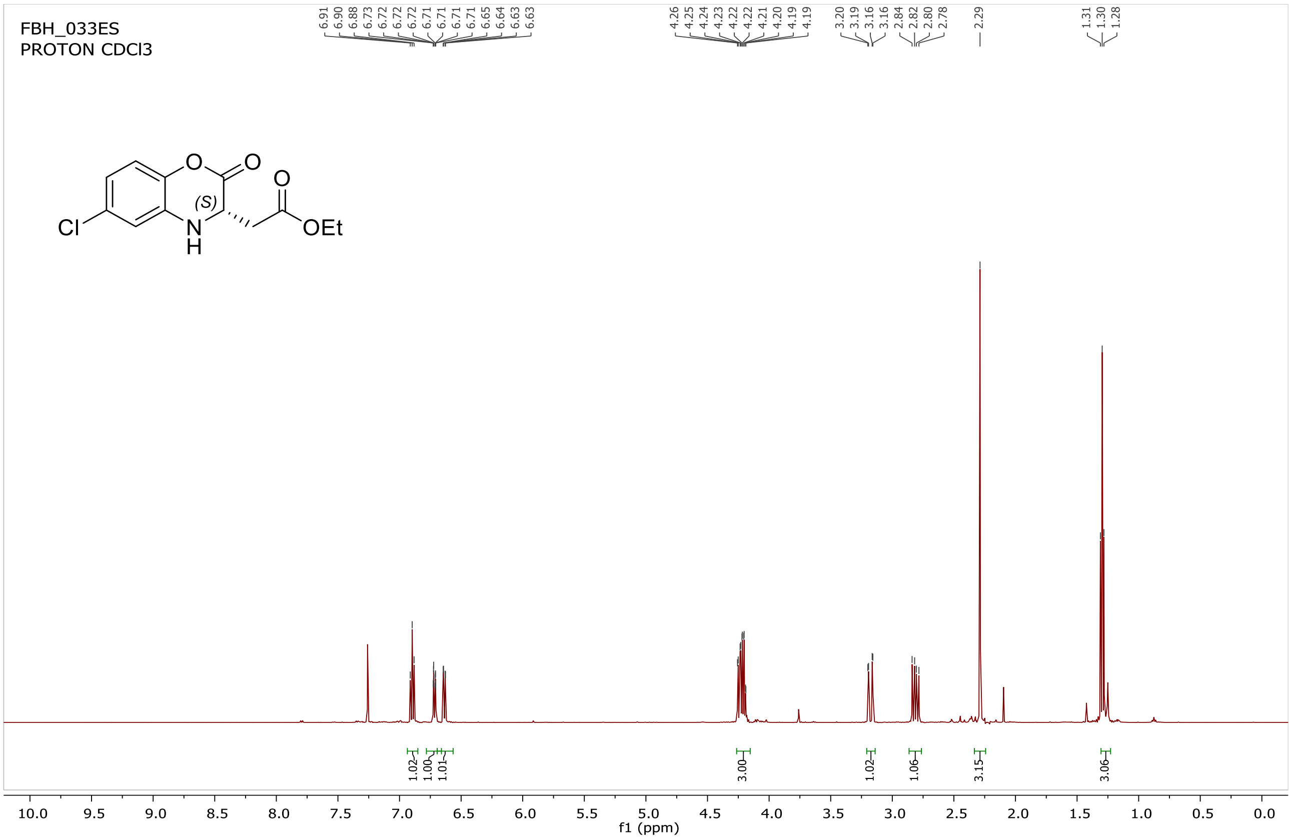


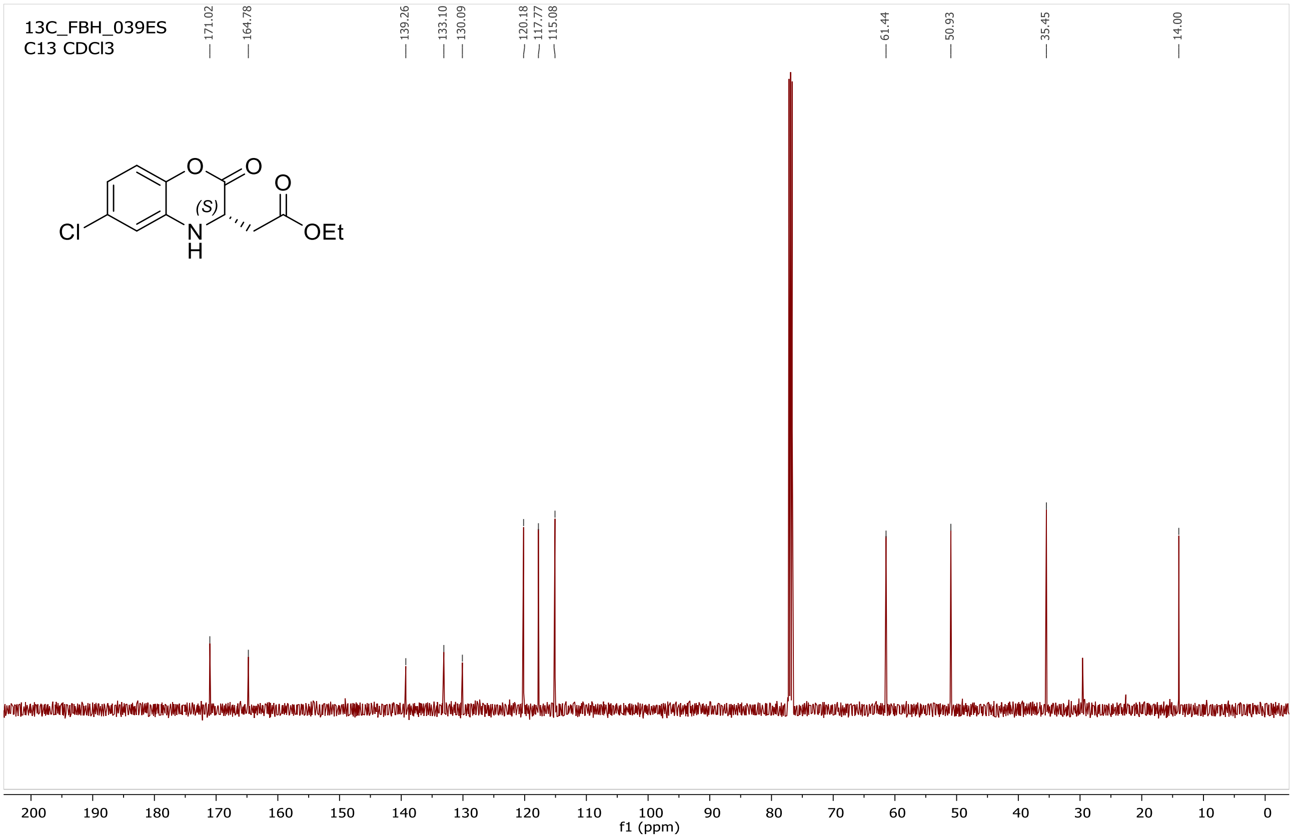


**Figure S16:**$^{1}$H NMR (top) and $^{13}$C NMR (bottom) of *(S)*-Ethyl 2-(6-Choro-2-oxo-3,4-dihydro-2H-benzo[b][1,4]oxazin-3-yl)acetate (**4g**).

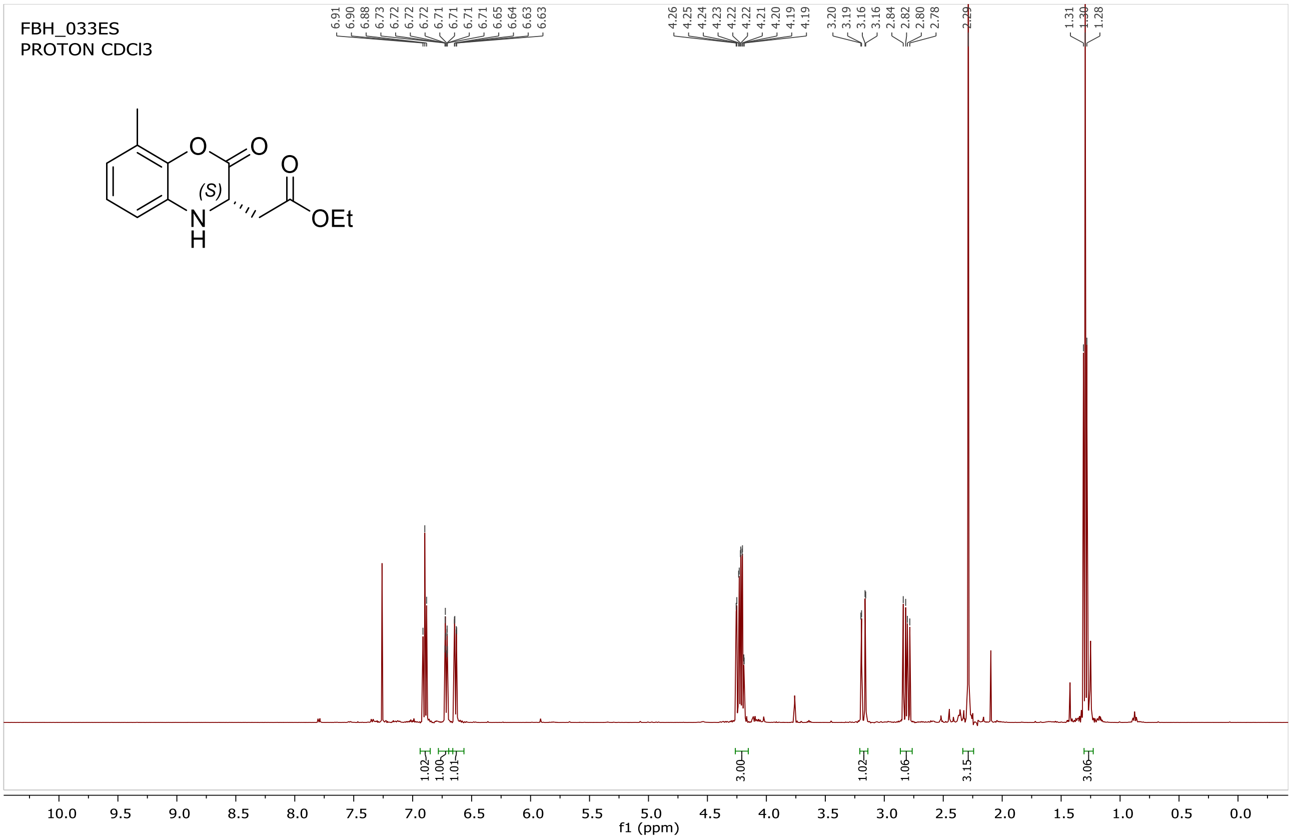



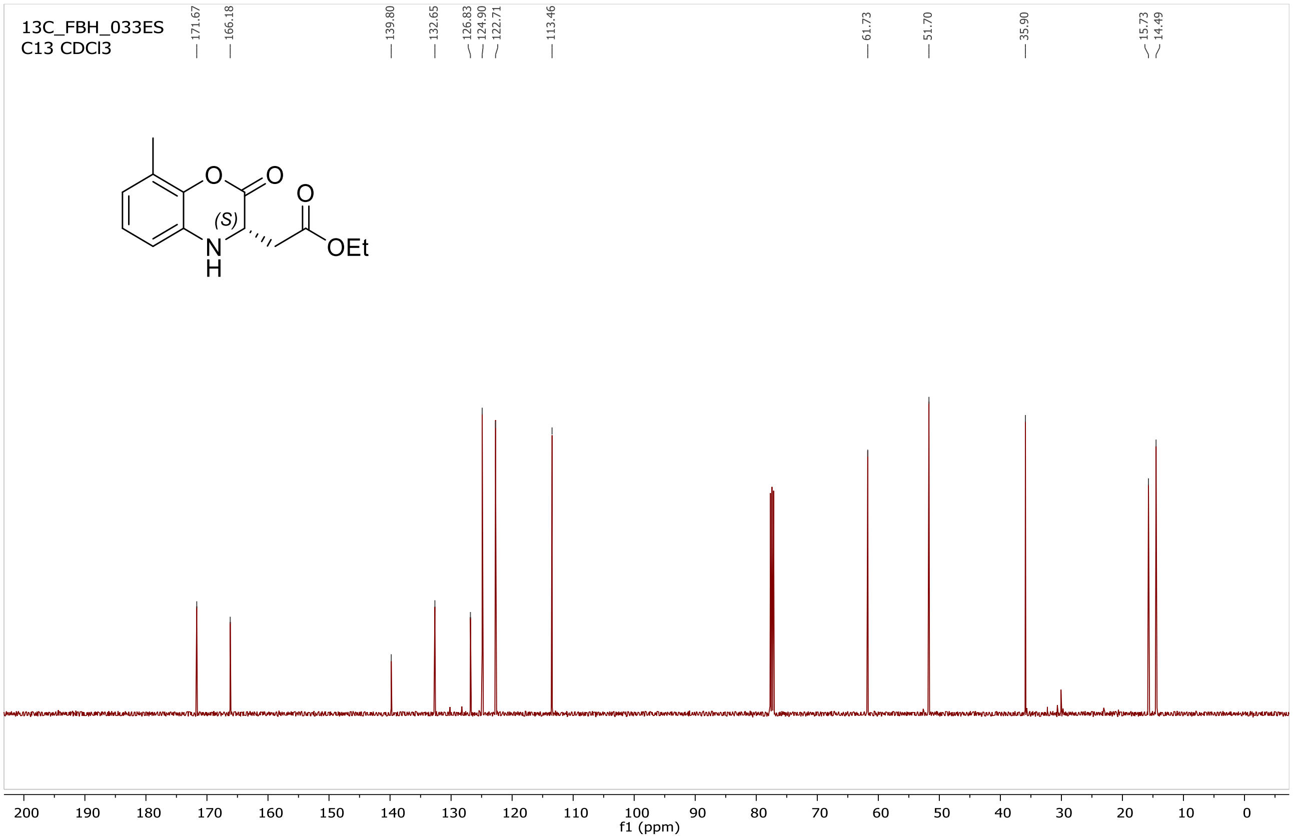



**Figure S17:** $^{1}$H NMR (top) and $^{13}$C NMR (bottom) of (*S*)-Ethyl 2-(8-methyl-2-oxo-3,4-dihydro-2H-benzo[b][1,4]oxazin-3-yl)acetate (**4h**)

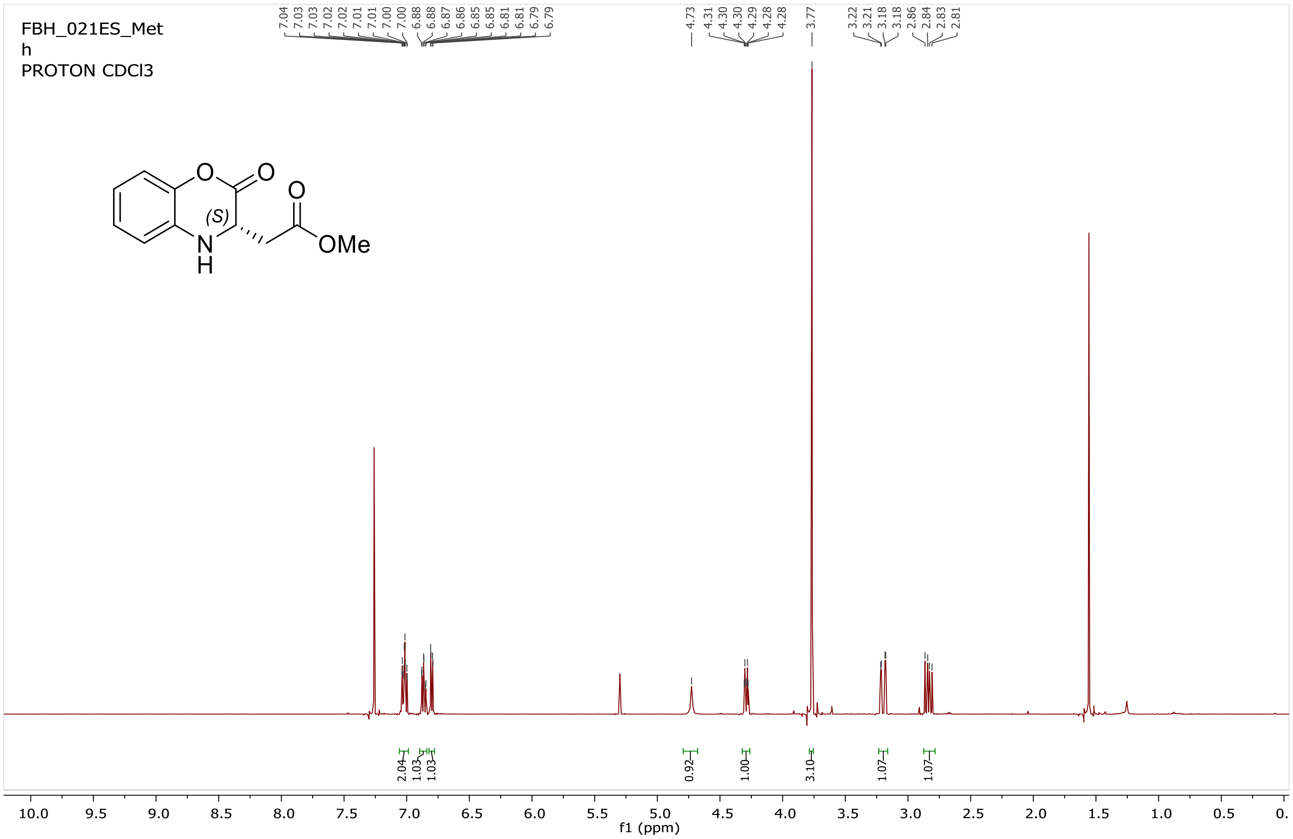


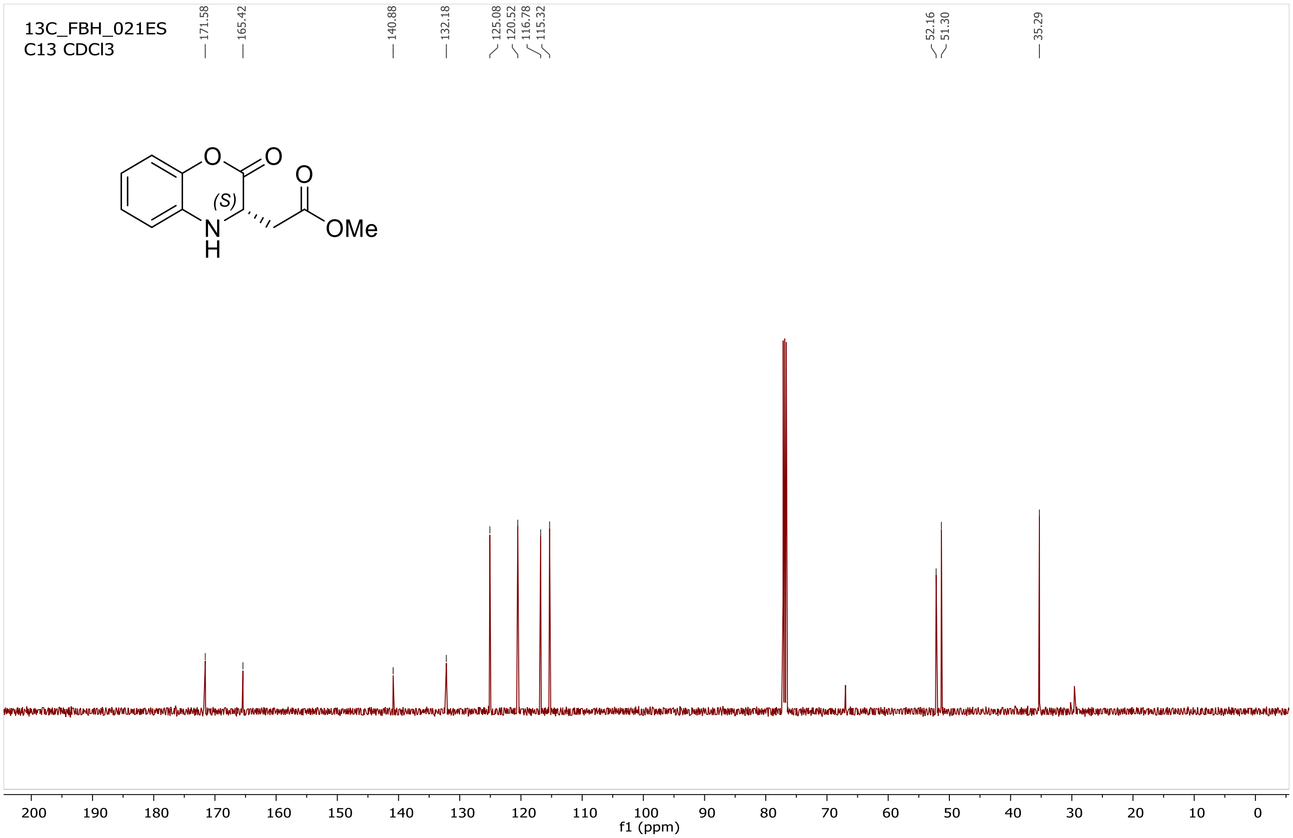


**Figure S18:** $^1$H NMR (top) and $^{13}$C NMR (bottom) of (*S*)-Methyl 2-(2-oxo-3,4-dihydro-2H-benzo[b][1,4]oxazin-3-yl)acetate (**4aa**)

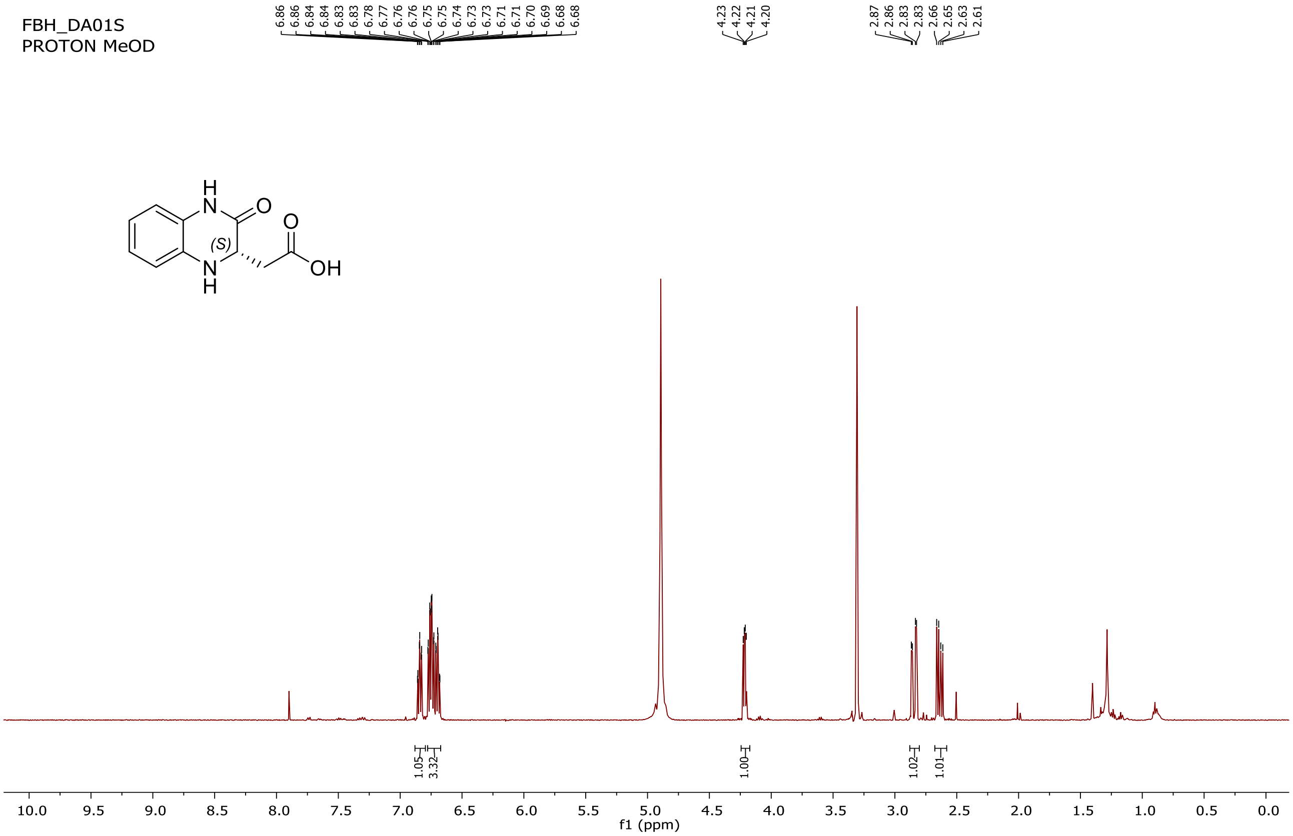


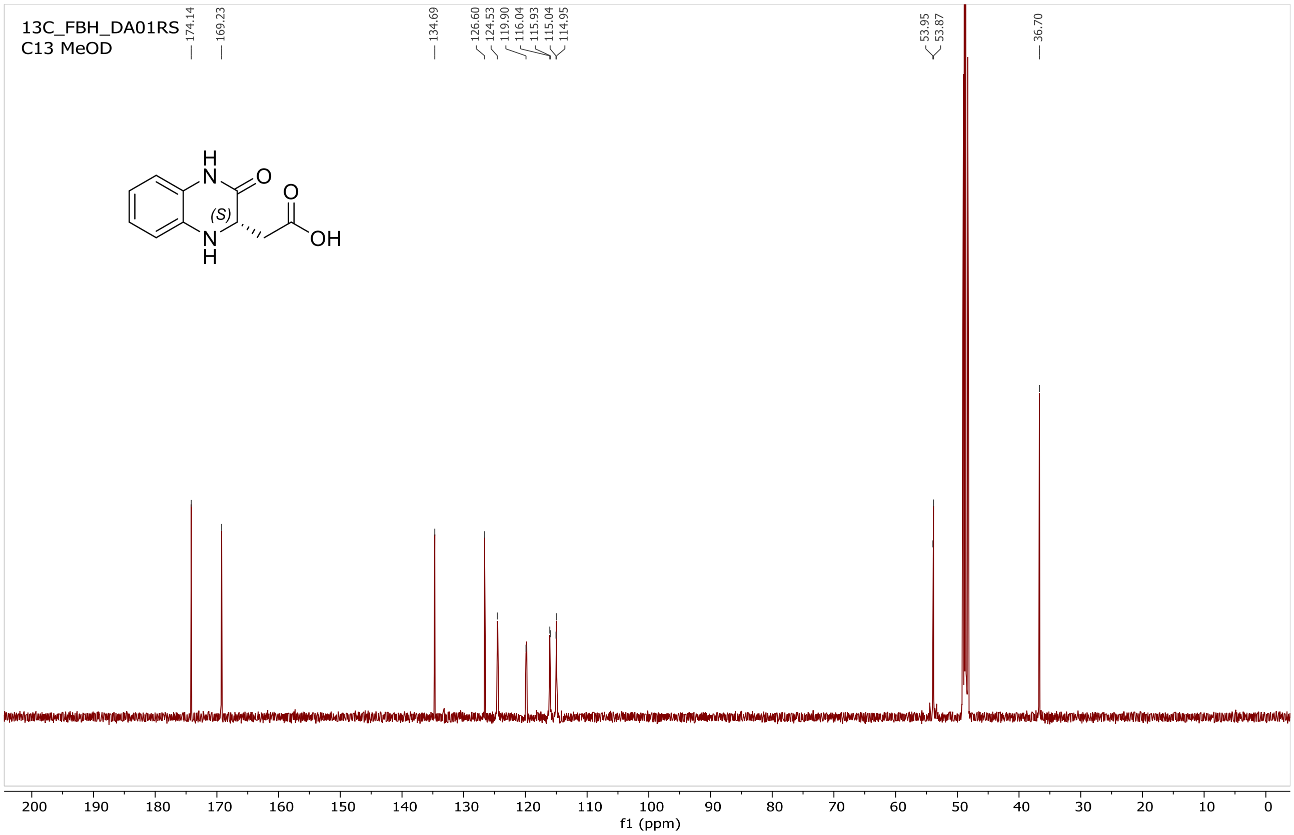


**Figure S19:** $^1$H NMR (top) and $^{13}$C NMR (bottom) of *(S)*-2-(3-oxo-1,2,3,4-tetrahydroquinoxalin-2-yl)acetic acid (**5p**)

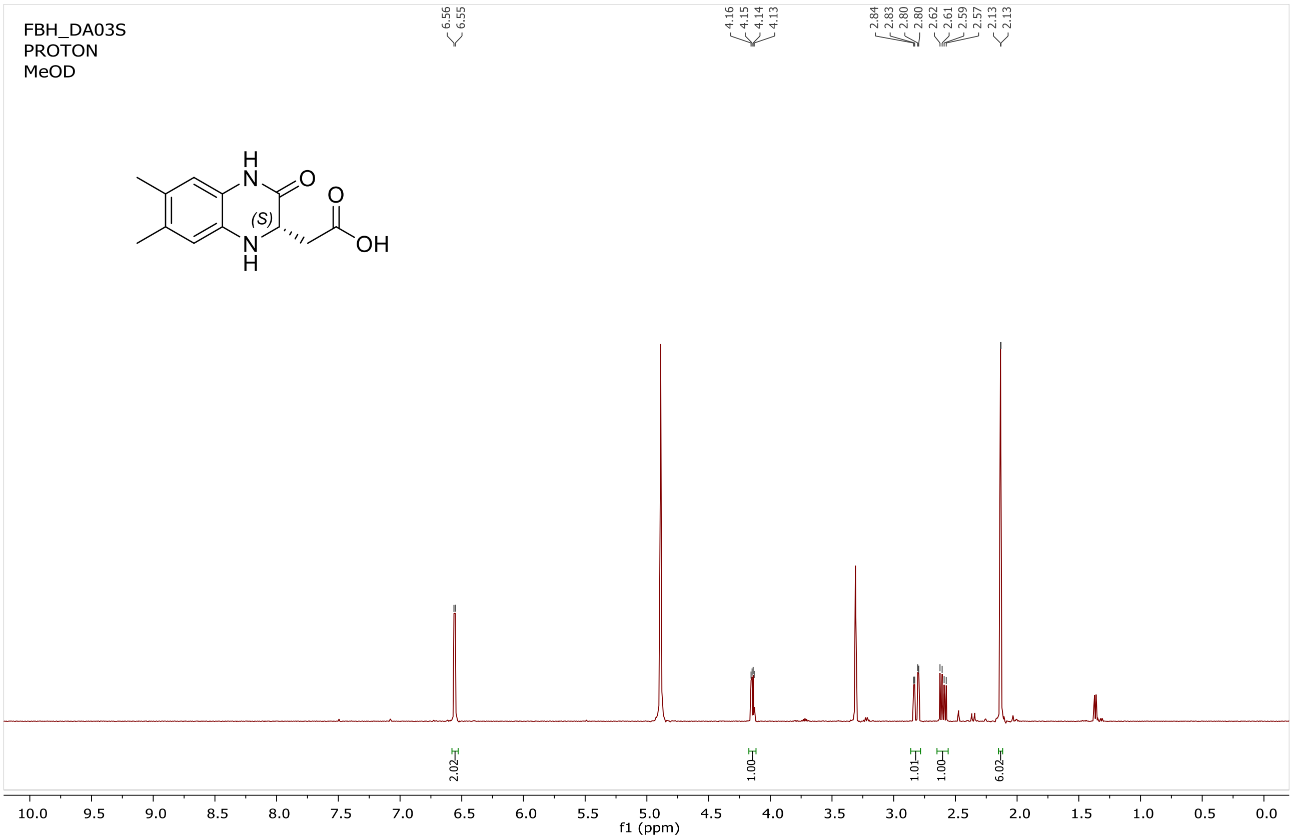


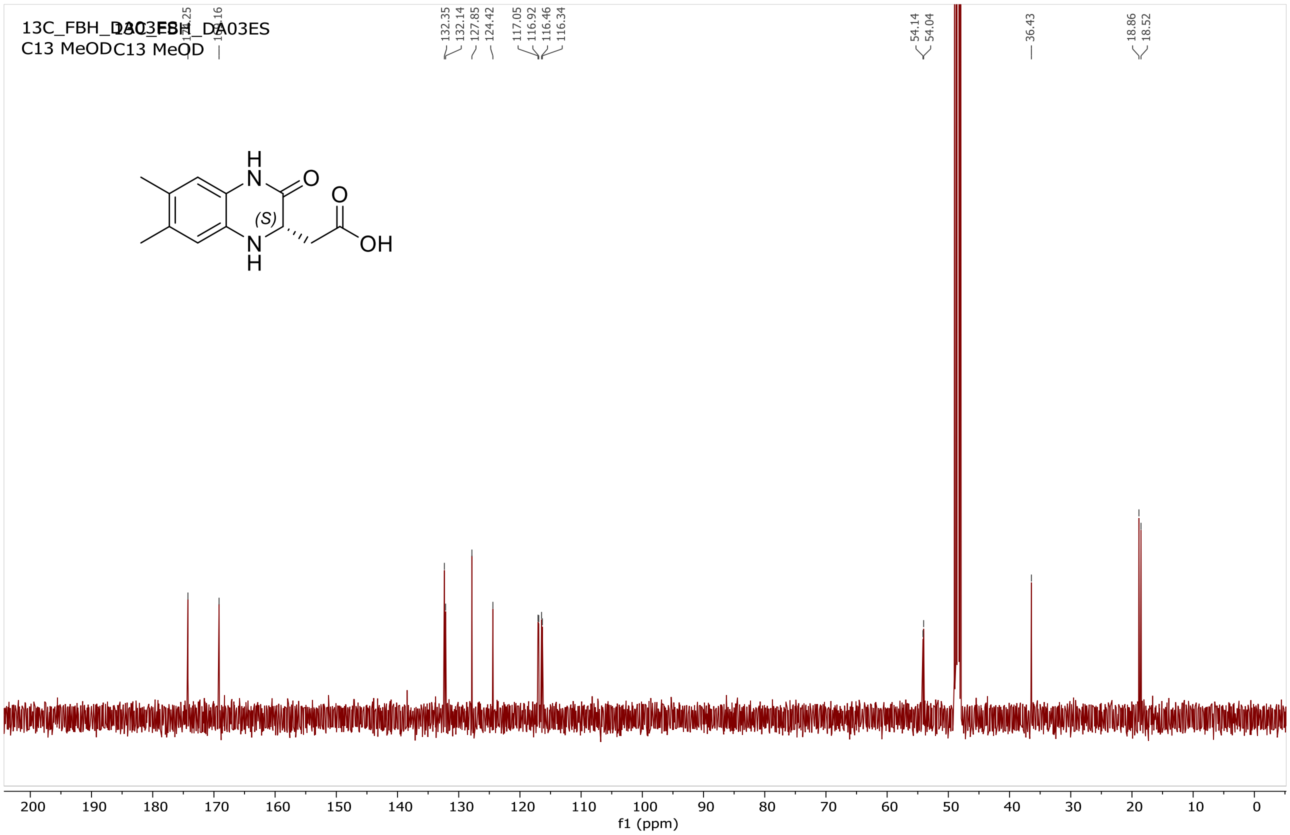


**Figure S20:** $^{1}$H NMR (top) and $^{13}$C NMR (bottom) of *(S)*-2-(6,7-dimethyl-3-oxo-1,2,3,4-tetrahydroquinoxalin-2-yl)acetic acid (**5q**)

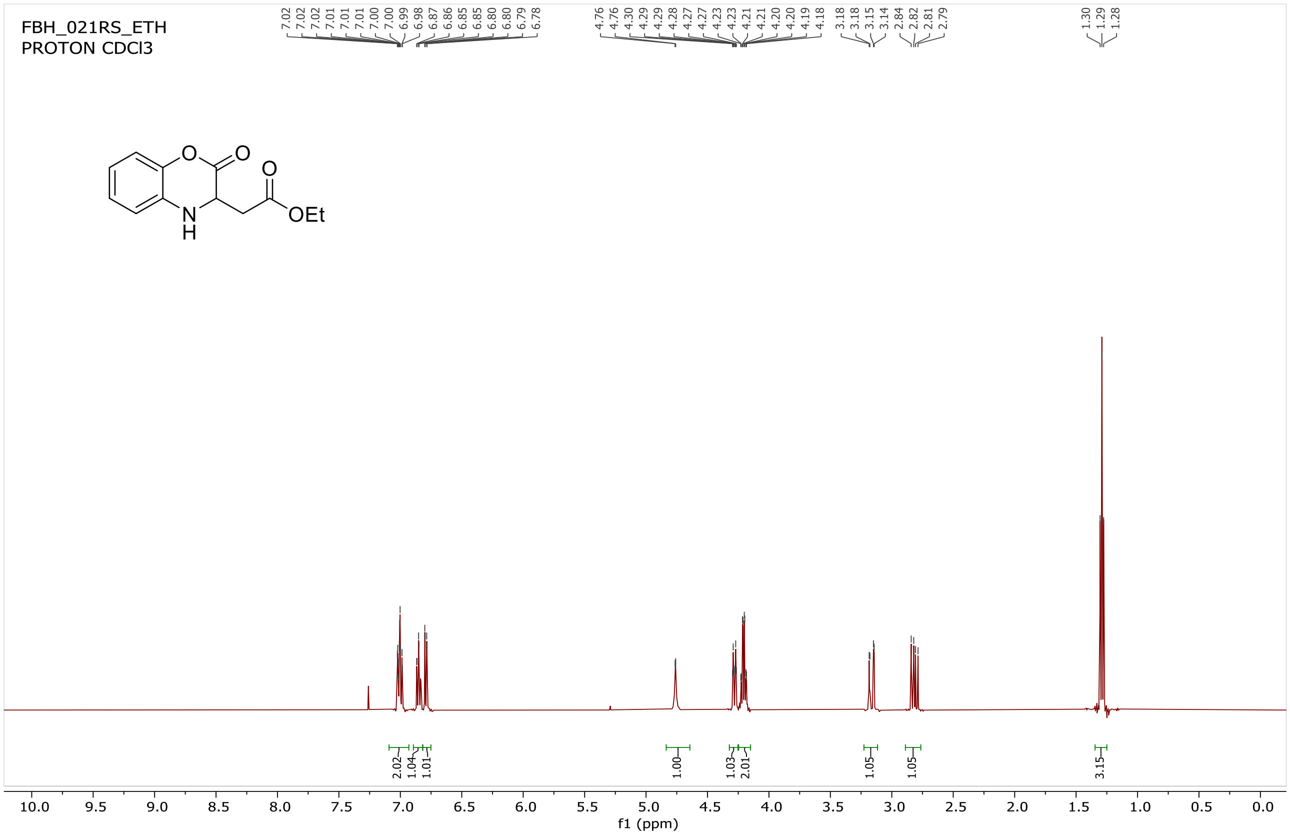


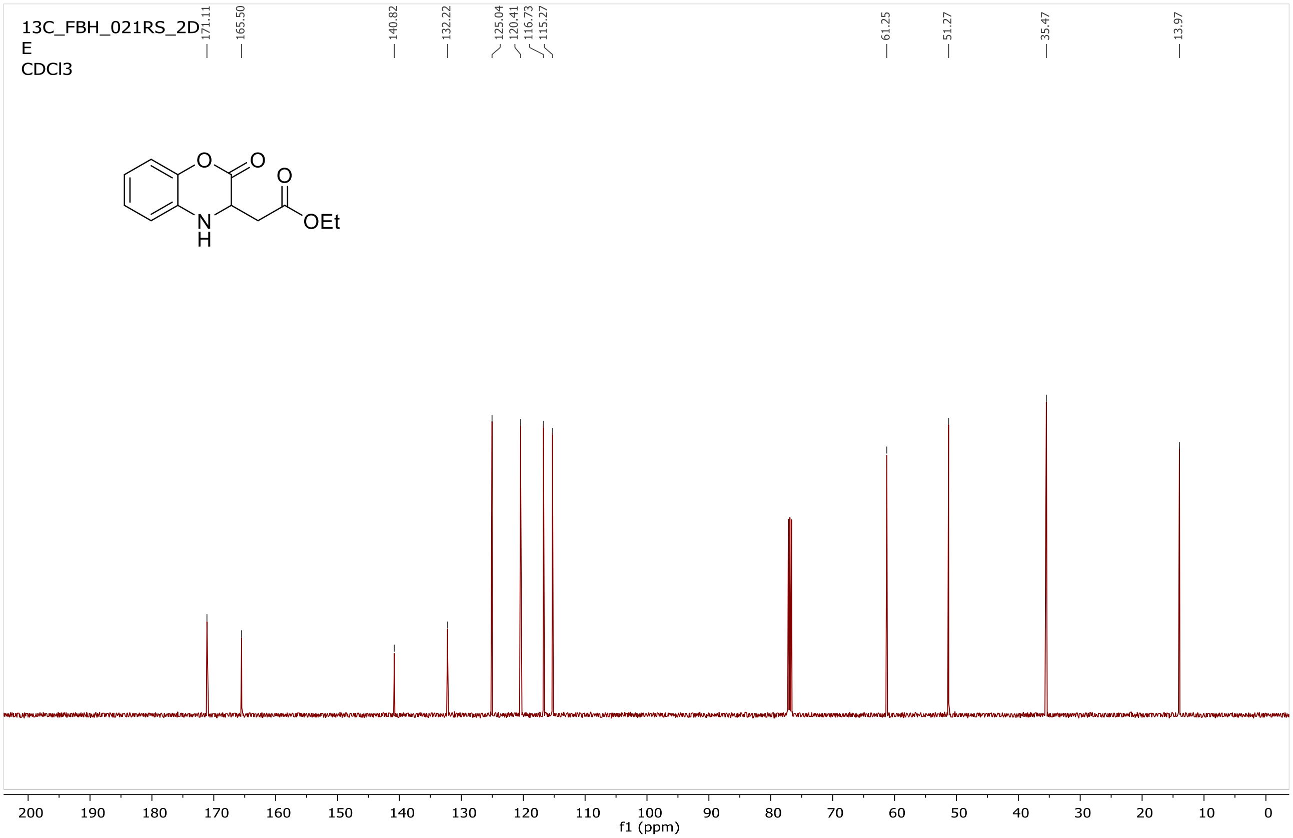


**Figure S21:** $^{1}$H NMR (top) and $^{13}$C NMR (bottom) of *rac*-Ethyl-2-(2-oxo-3,4-dihydro-2H-benzo[b][1,4]oxazin-3-yl)acetate (**7a**)

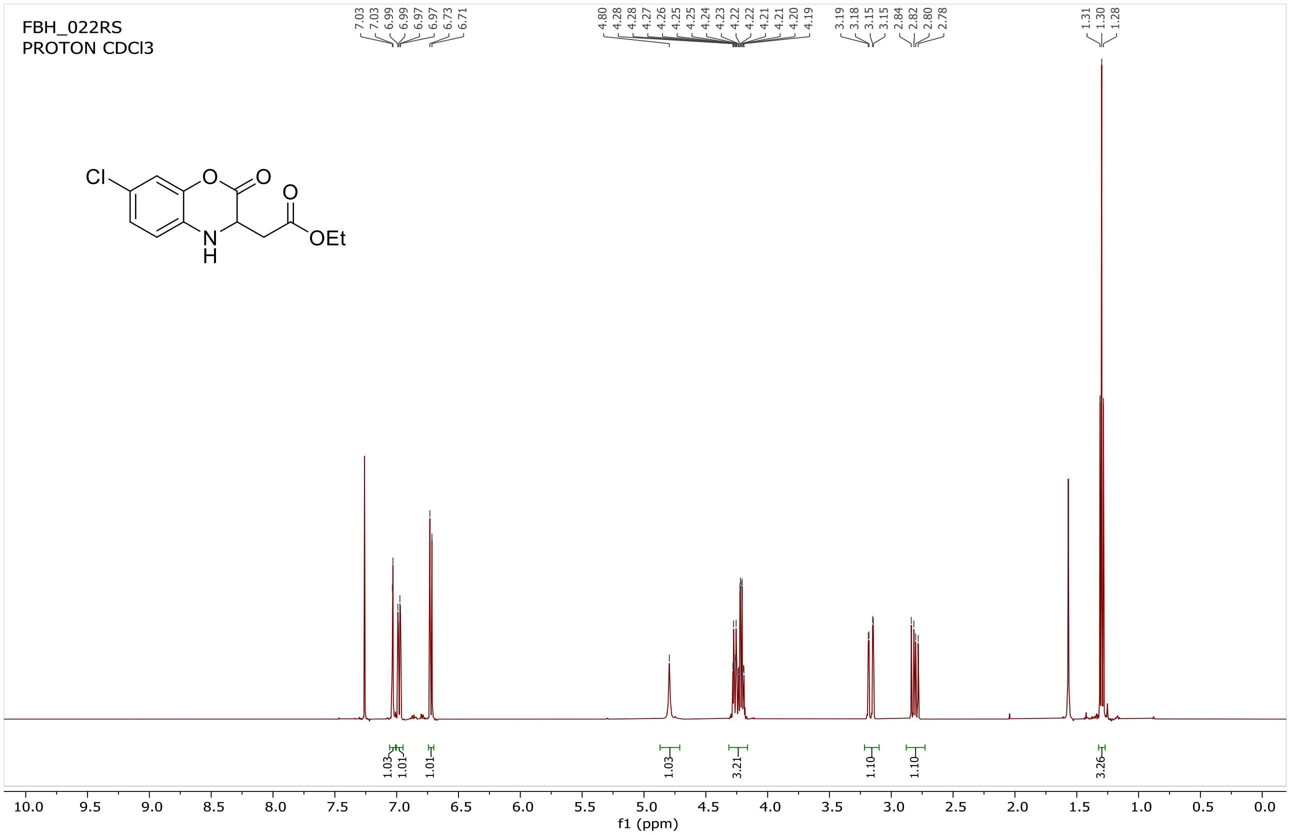


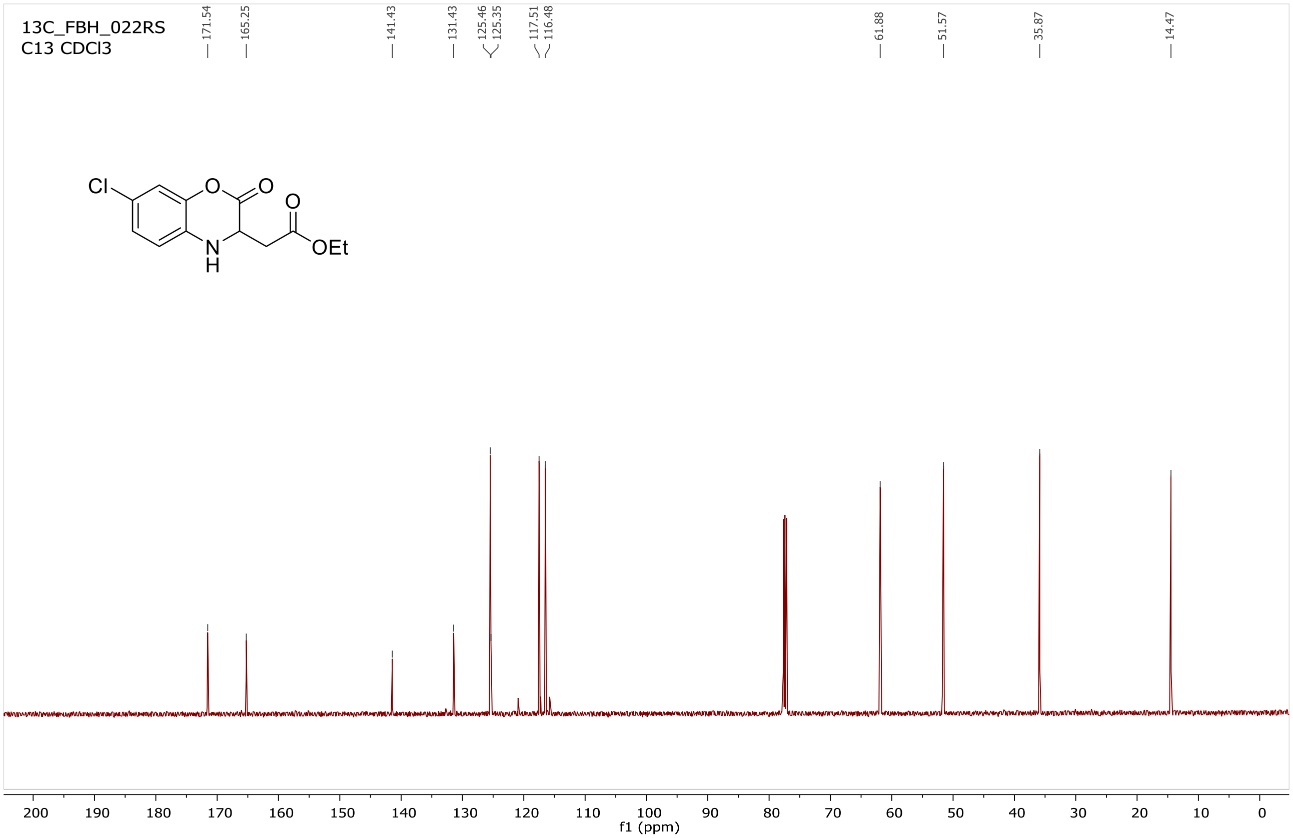


**Figure S22:**$^{1}$H NMR (top) and $^{13}$C NMR (bottom) of *rac*-Ethyl 2-(7-chloro-2-oxo-3,4-dihydro-2H-benzo[b][1,4]oxazin-3-yl)acetate (**7b**)

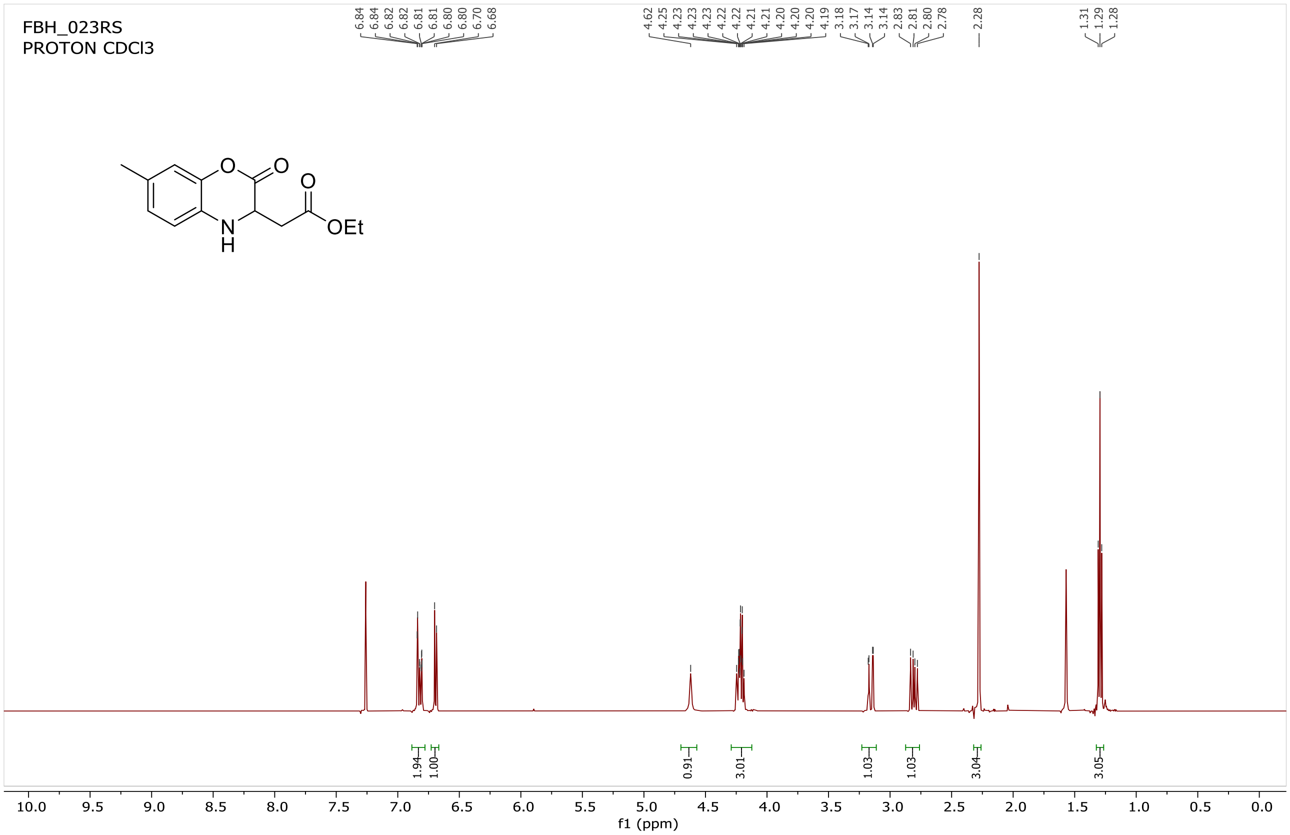


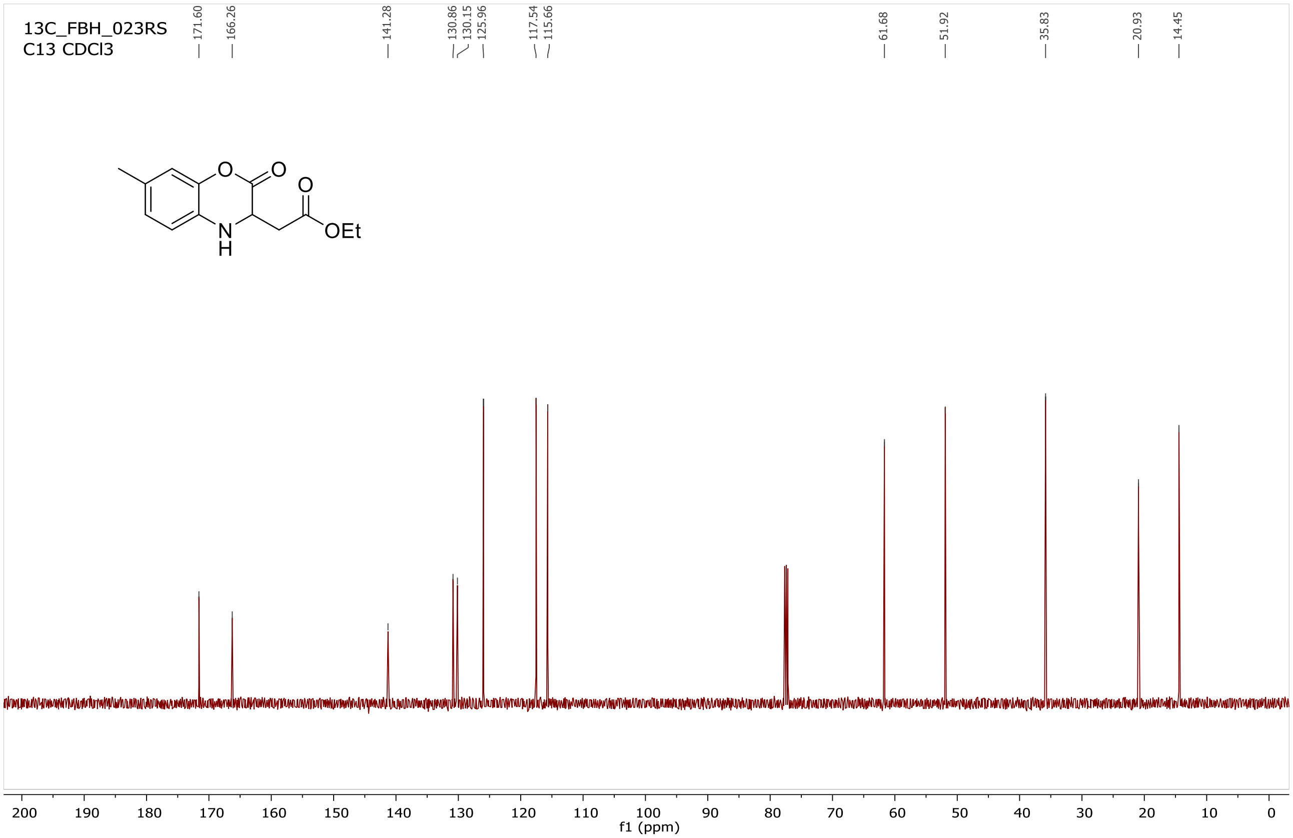


**Figure S23:** $^1$H NMR (top) and $^{13}$C NMR (bottom) of *rac*-Ethyl 2-(7-methyl-2-oxo-3,4-dihydro-2H-benzo[b][1,4]oxazin-3-yl)acetate(**7c**)

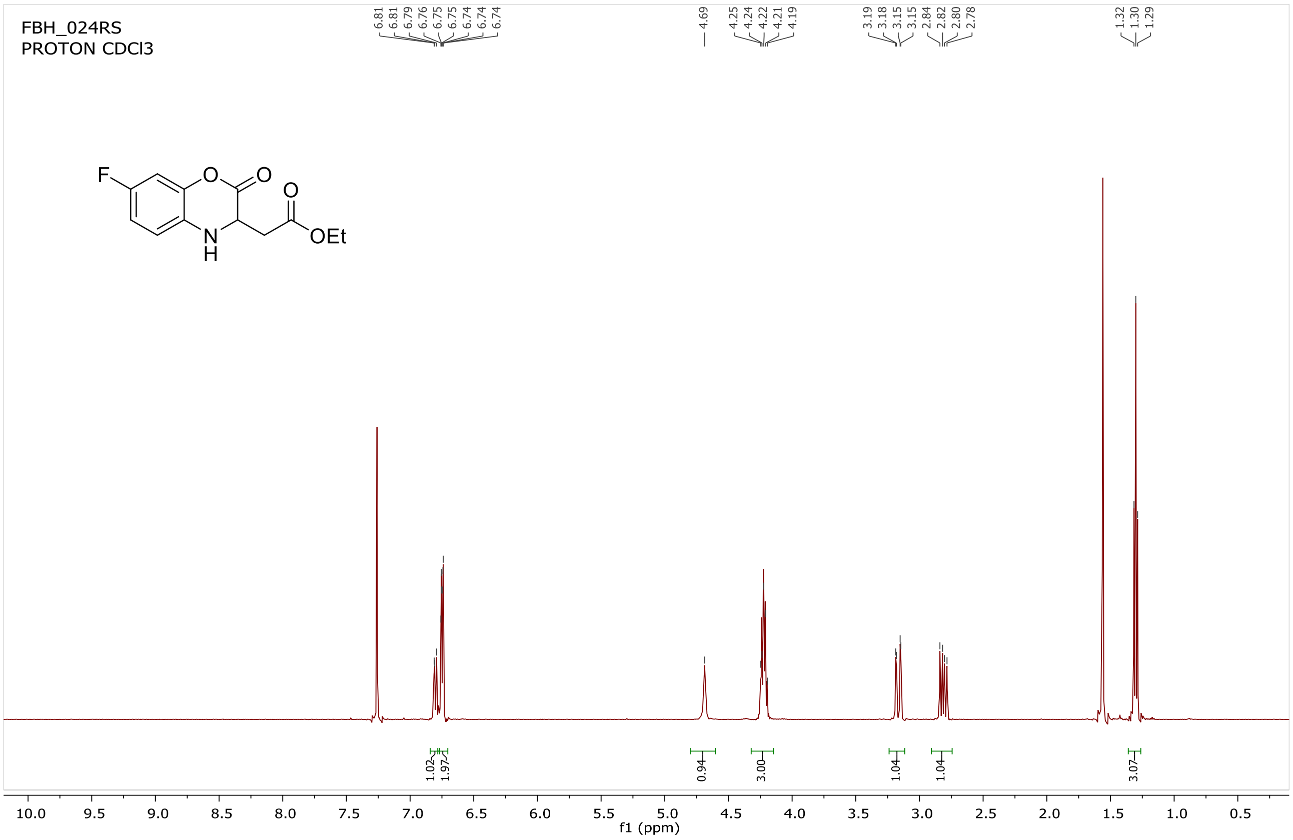

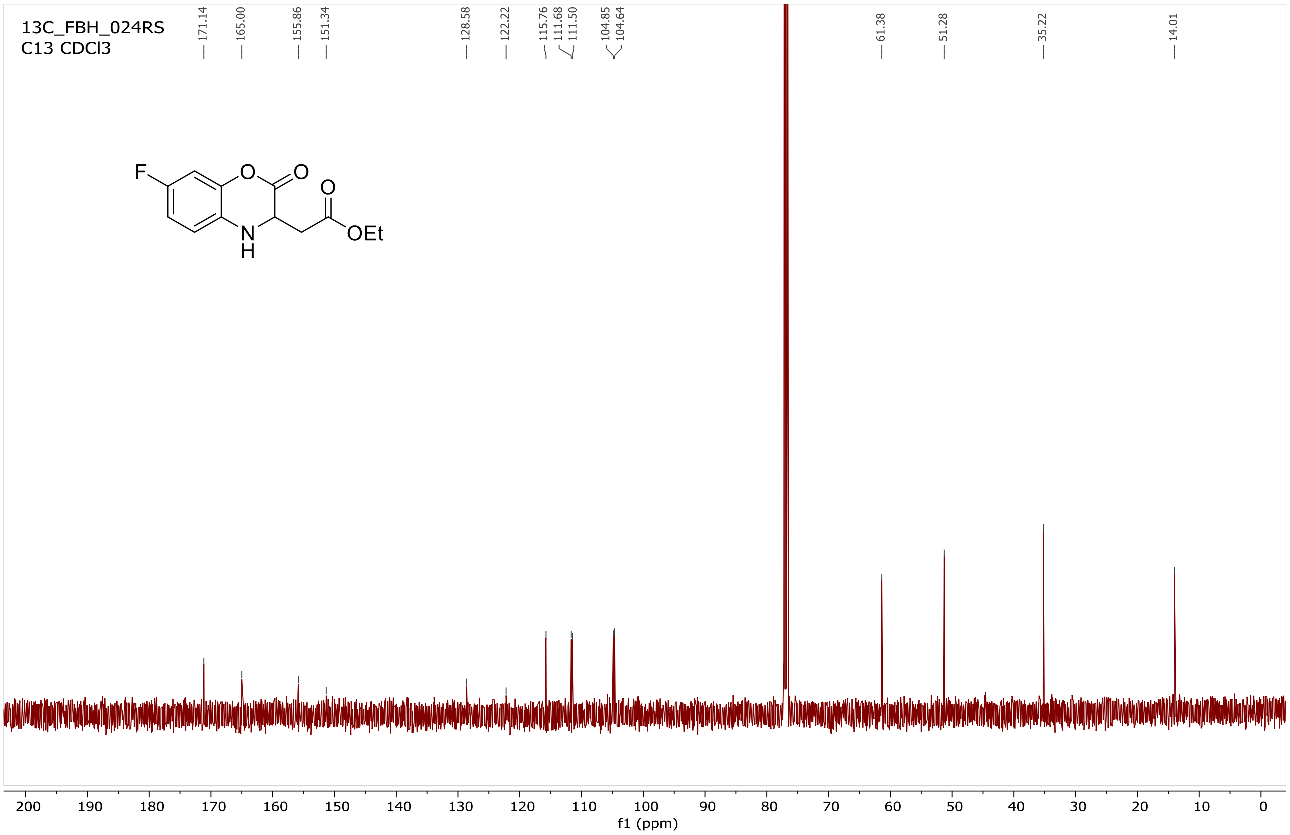

**Figure S24:**$^{1}$H NMR (top) and $^{13}$C NMR (bottom) of *rac*-Ethyl 2-(7-fluoro-2-oxo-3,4-dihydro-2H-benzo[b][1,4]oxazin-3-yl)acetate (**7d**)

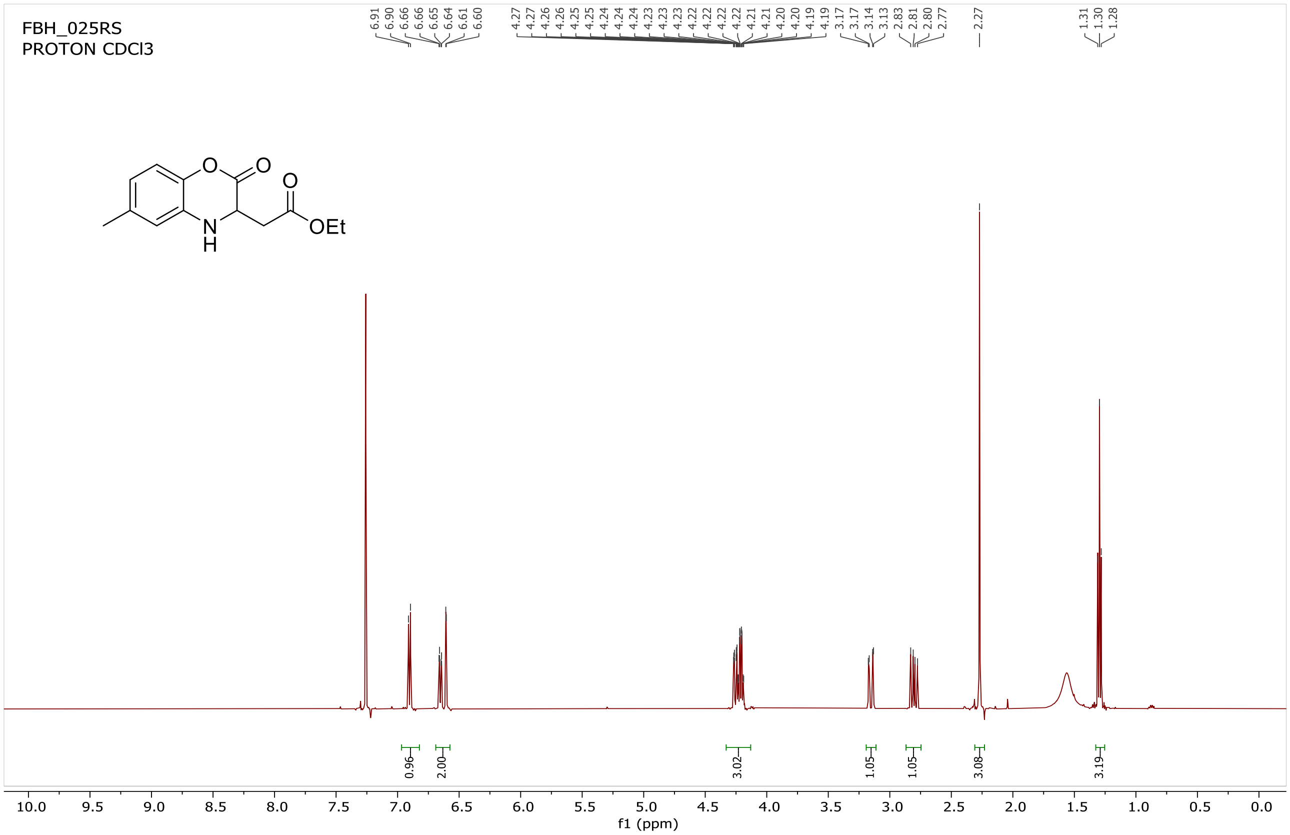


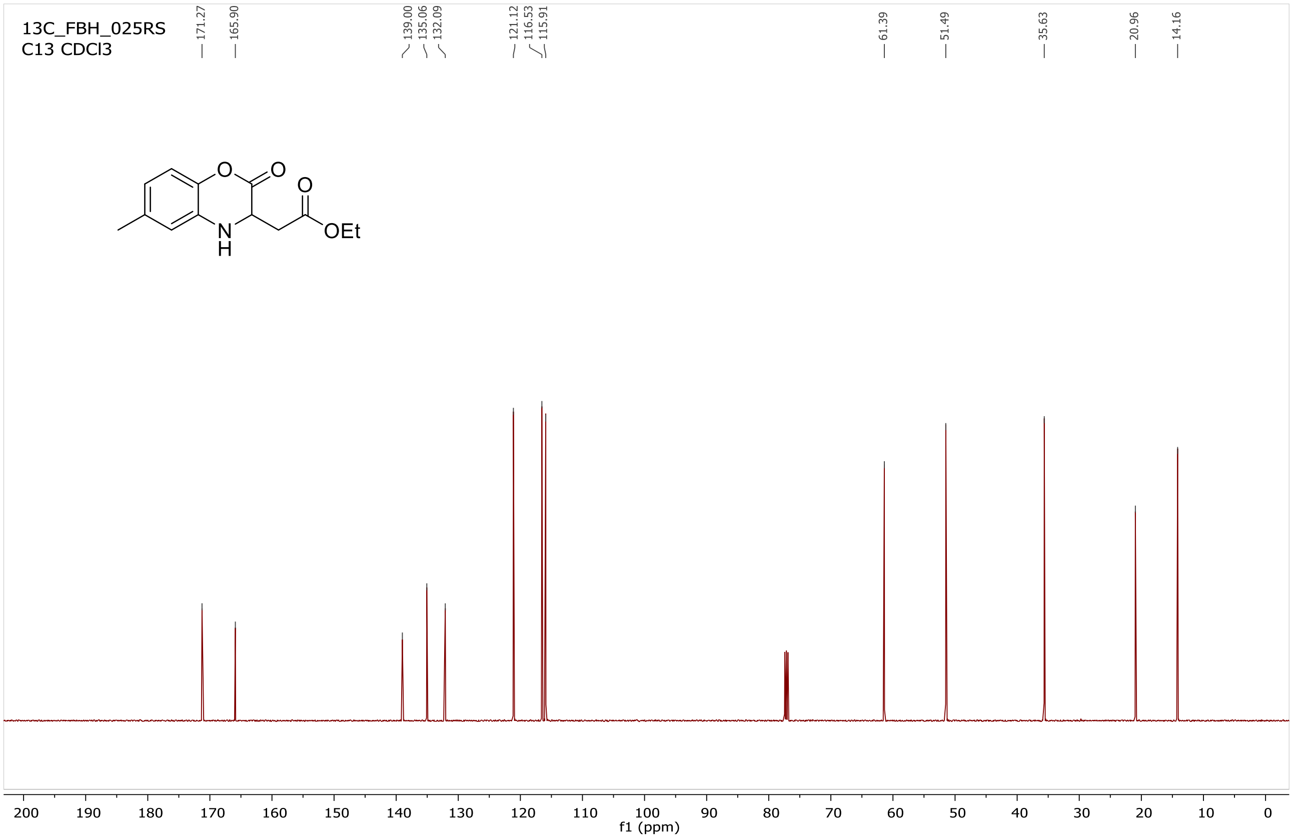


**Figure S25:** $^1$H NMR (top) and $^{13}$C NMR (bottom) of *rac*-Ethyl 2-(6-methyl-2-oxo-3,4-dihydro-2H-benzo[b][1,4]oxazin-3-yl)acetate (**7f**)

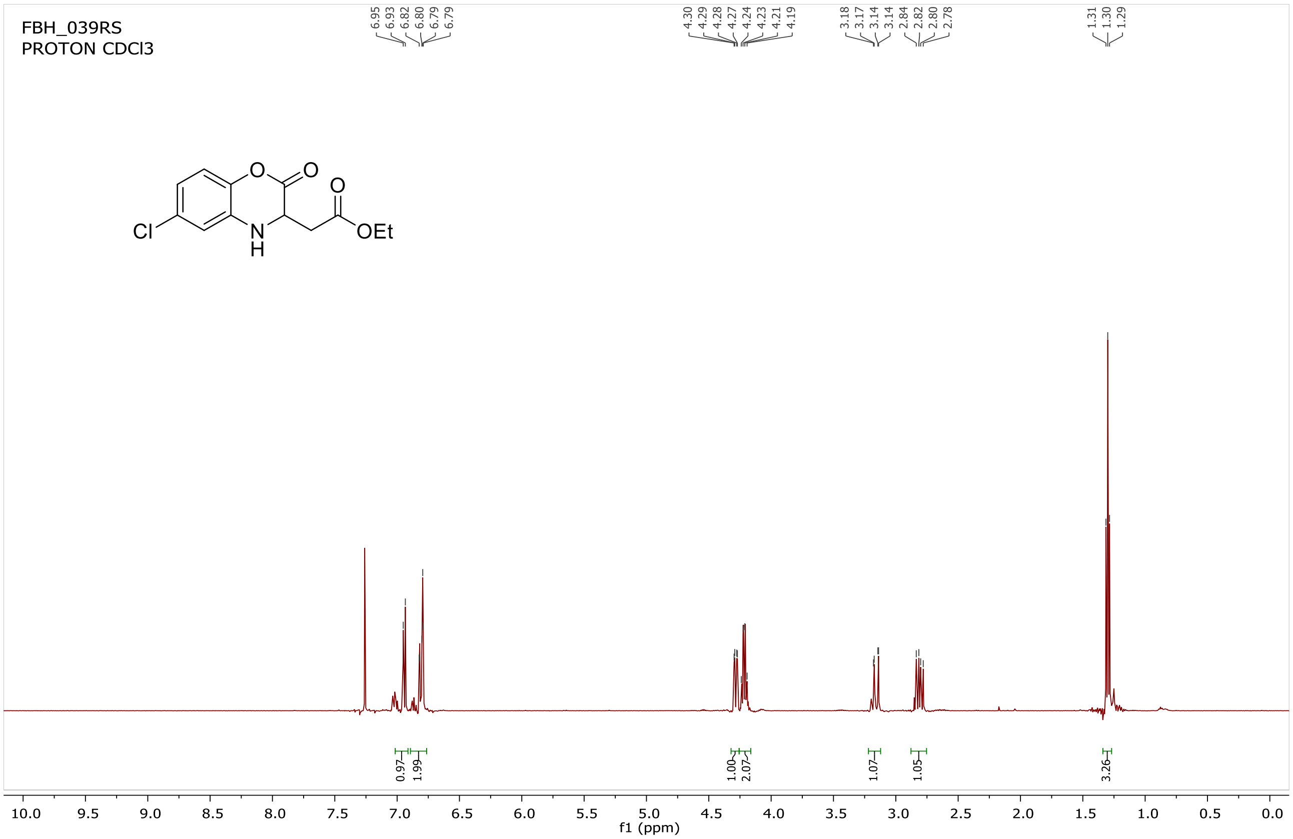


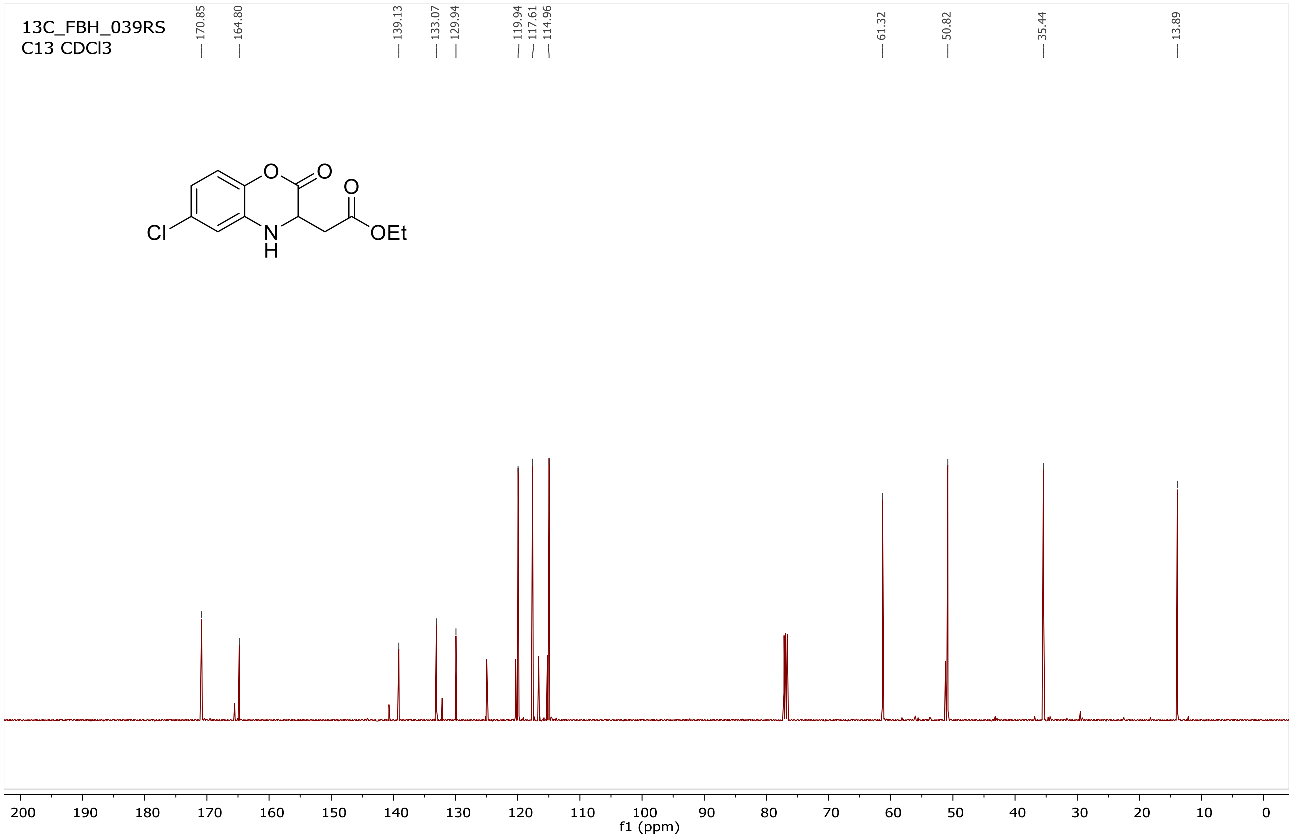


**Figure S26:**$^{1}$H NMR (top) and $^{13}$C NMR (bottom) of *rac*-Ethyl 2-(6-chloro-2-oxo-3,4-dihydro-2H-benzo[b][1,4]oxazin-3yl)acetate (**7g**)

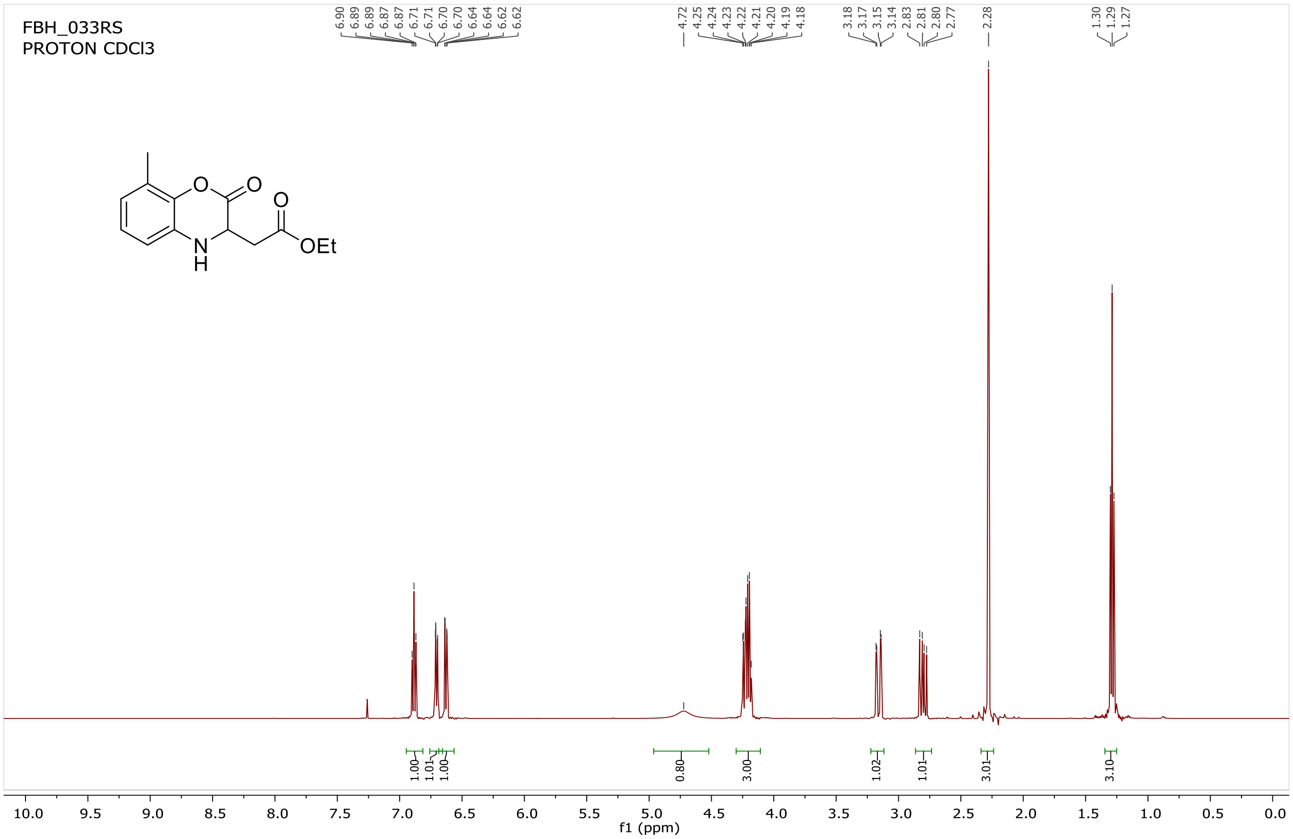


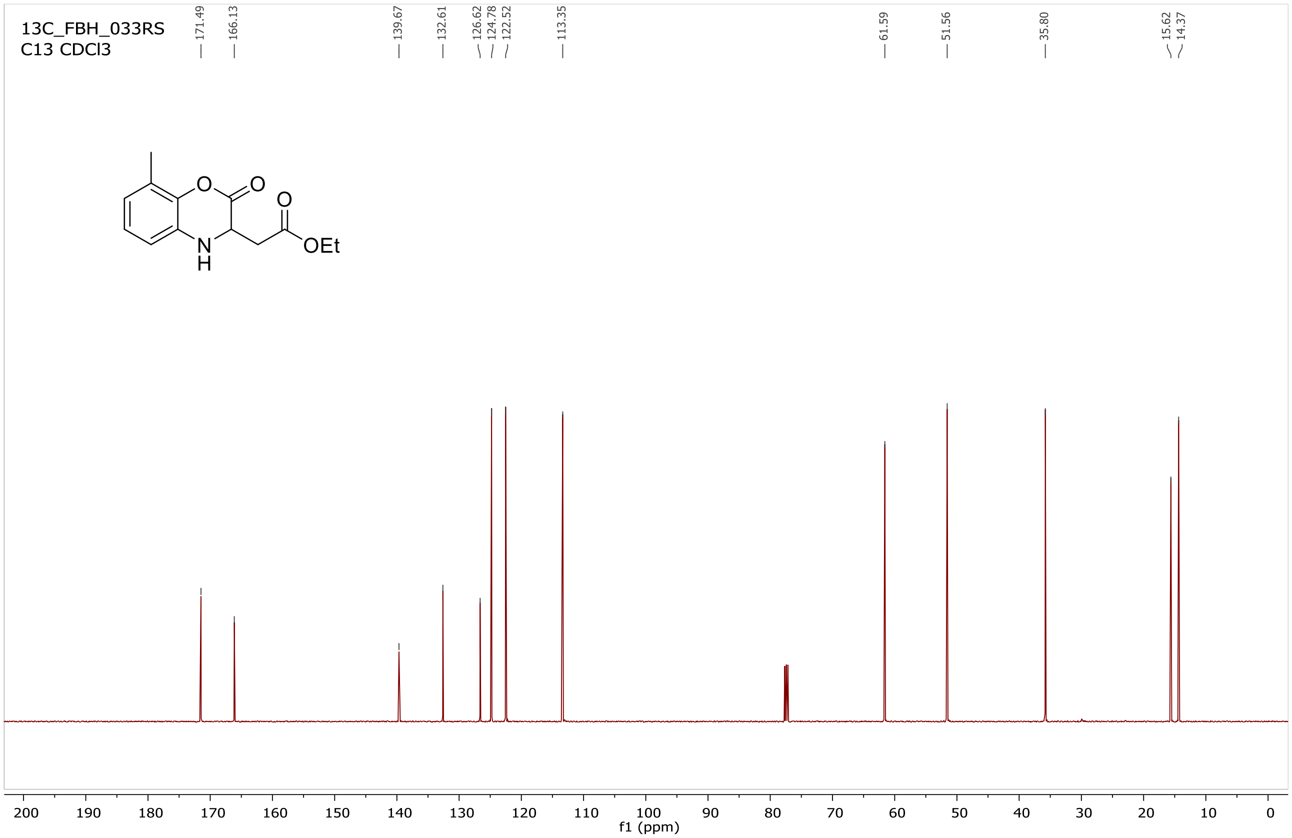


**Figure S27:**$^{1}$H NMR (top) and $^{13}$C NMR (bottom) of *rac*-Ethyl 2-(8-methyl-2-oxo-3,4-dihydro-2H-benzo[b][1,4]oxazin-3 yl)acetate (**7h**)

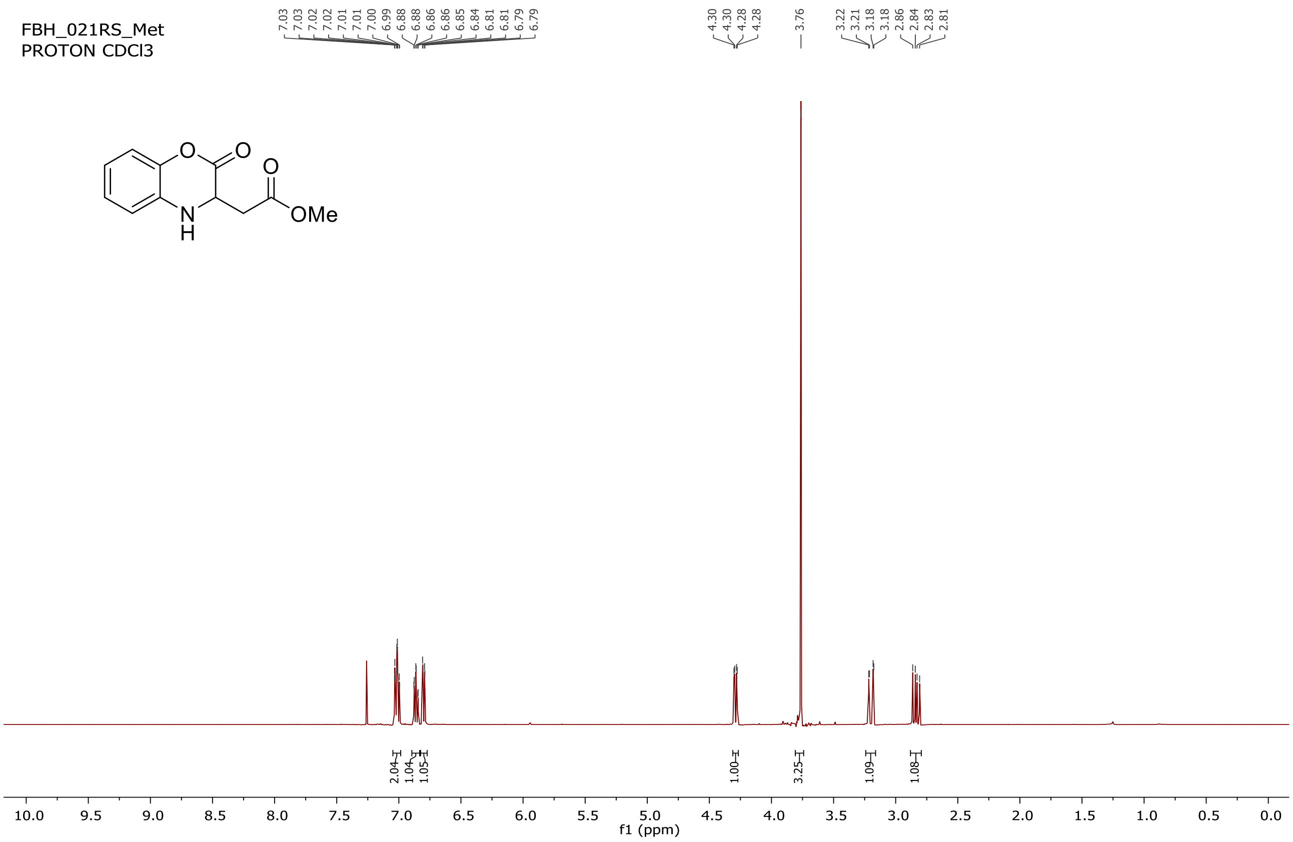


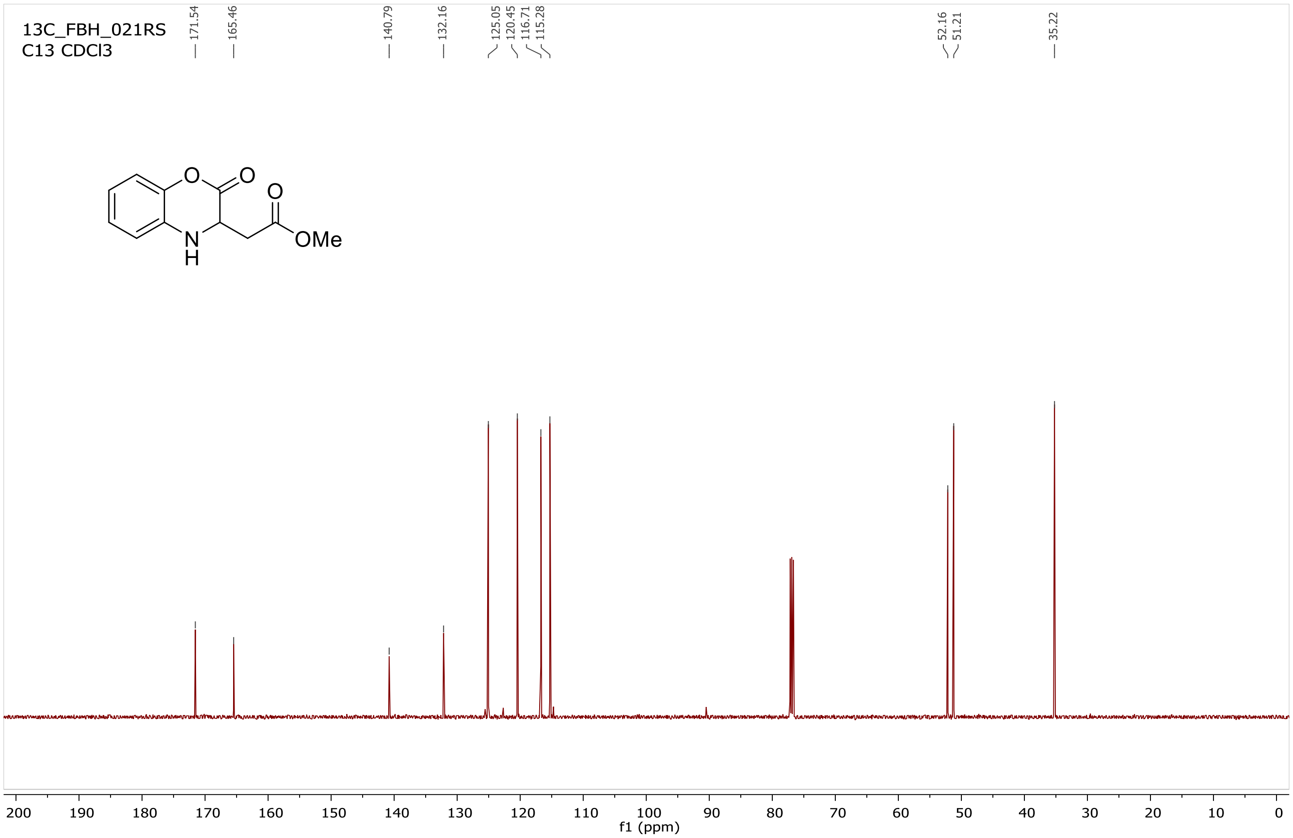


**Figure S28:** $^{1}H$ NMR (top) and $^{13}C$ NMR (bottom) of *rac*-Methyl 2-(2-oxo-3,4-dihydro-2H-benzo[b][1,4]oxazin-3-yl)acetate (**7aa**)

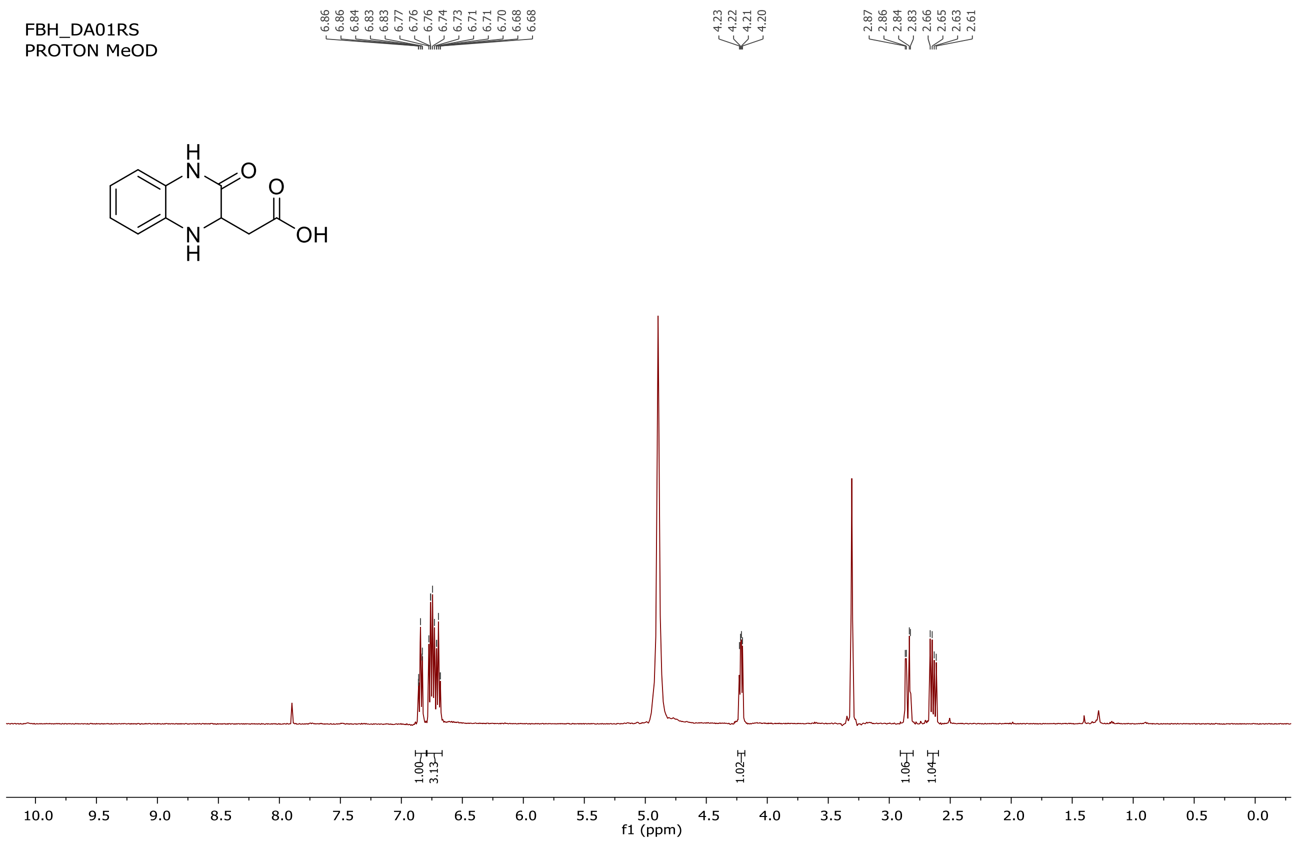


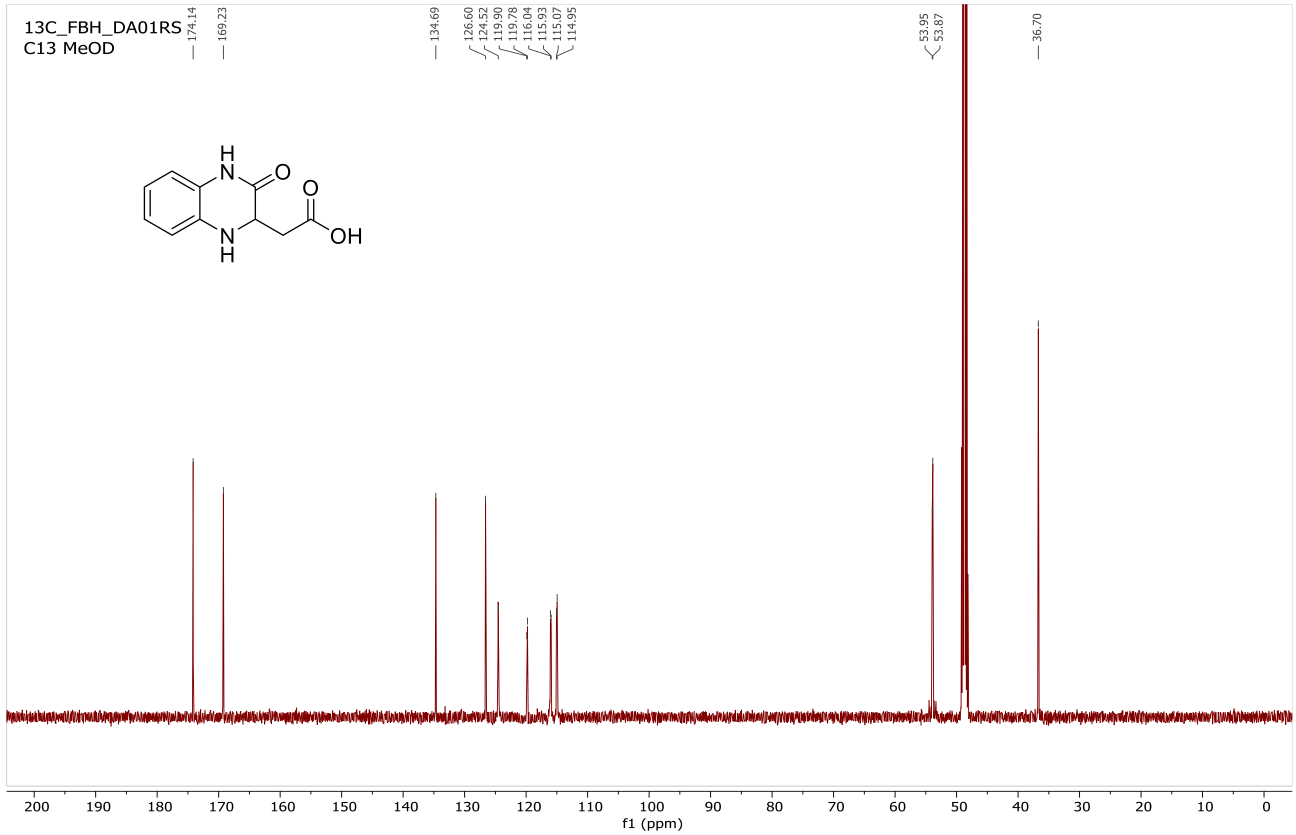


**Figure S29:** $^{1}H$ NMR (top) and $^{13}C$ NMR (bottom) of *rac*-2-(3-oxo-1,2,3,4-tetrahydroquinoxalin-2-yl)acetic acid (**9**)

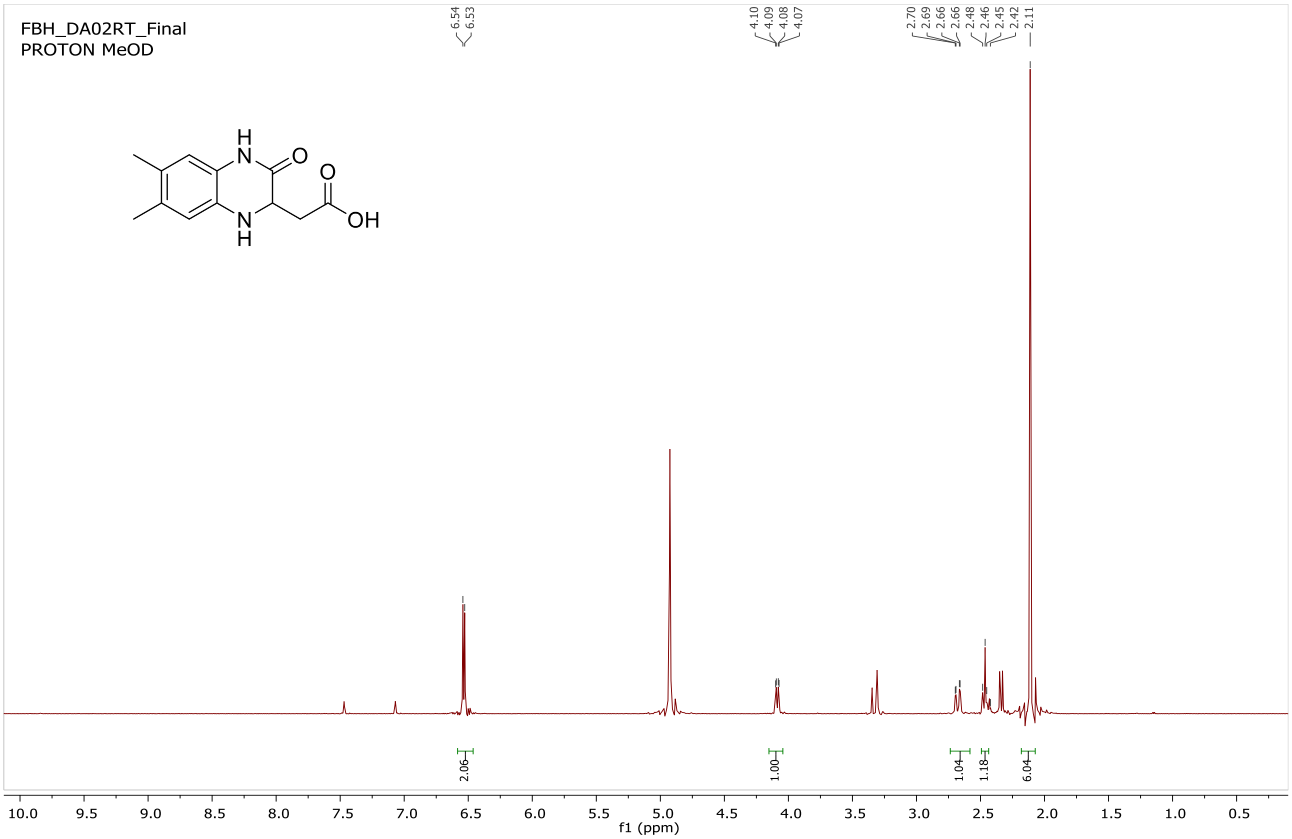


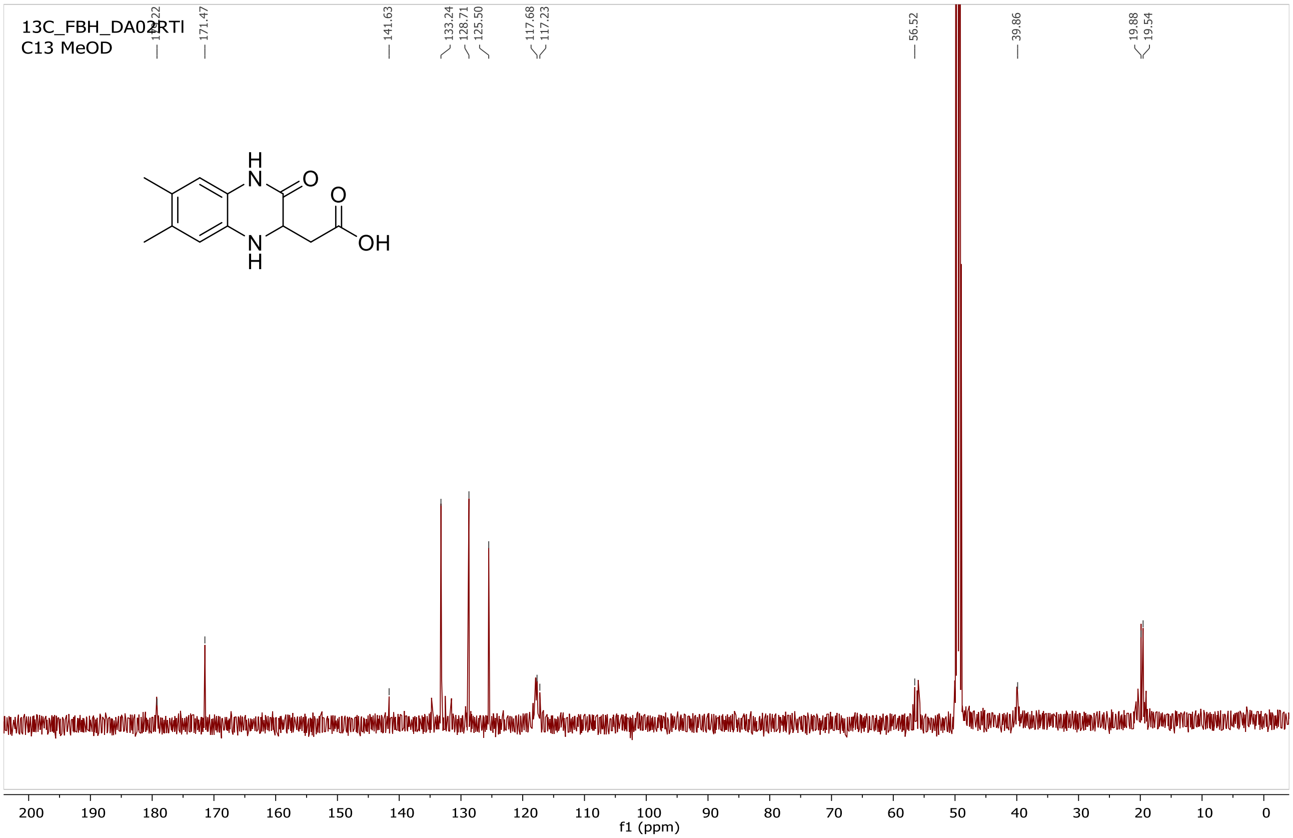


**Figure S30:**$^{1}$H NMR (top) and $^{13}$C NMR (bottom) of 2-(6,7-dimethyl-3-oxo-1,2,3,4-tetrahydroquinoxalin-2-yl)acetic acid (**11**)

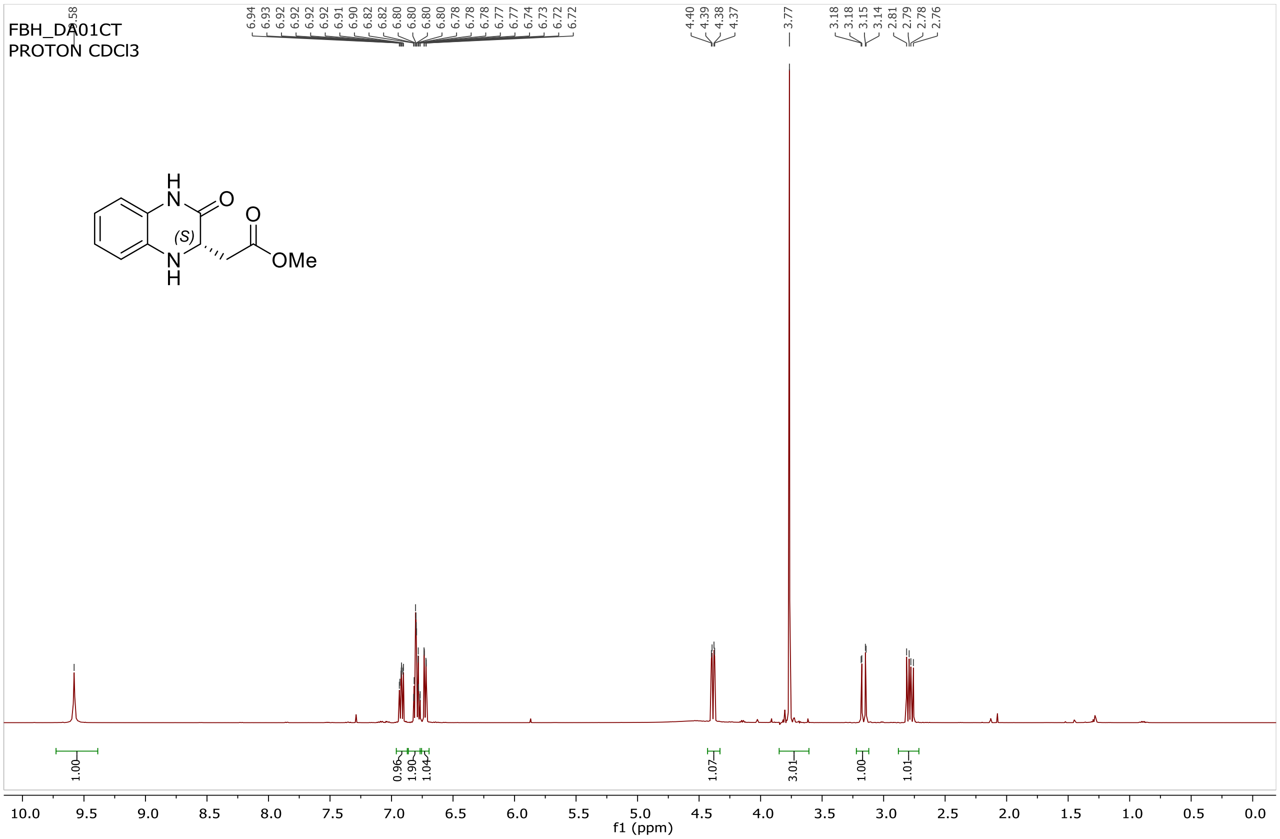


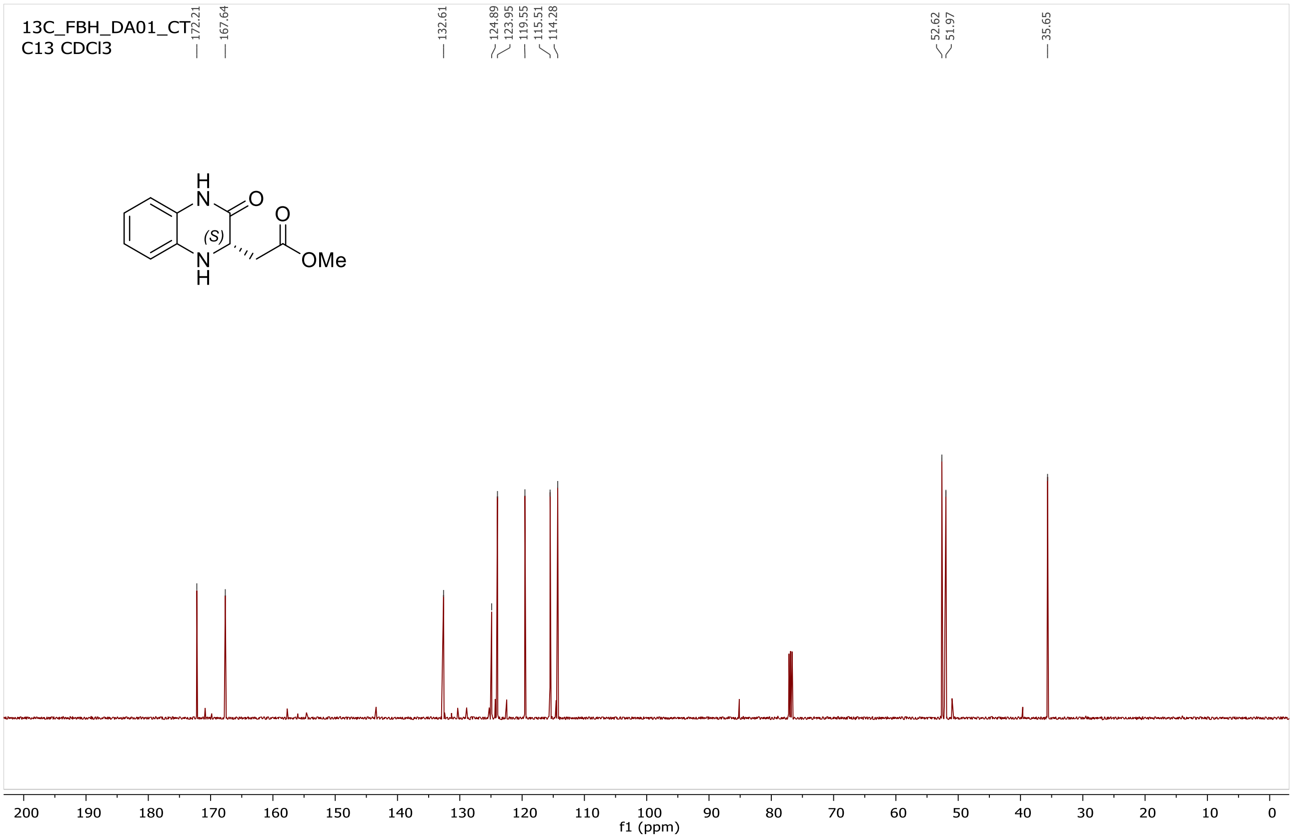


**Figure S31:** $^{1}$H NMR (top) and $^{13}$C NMR (bottom) of (*S*)-Methyl 2-(2-oxo-3,4-dihydro-2H-benzo[b][1,4]oxazin-3-yl)acetate (**S5**)

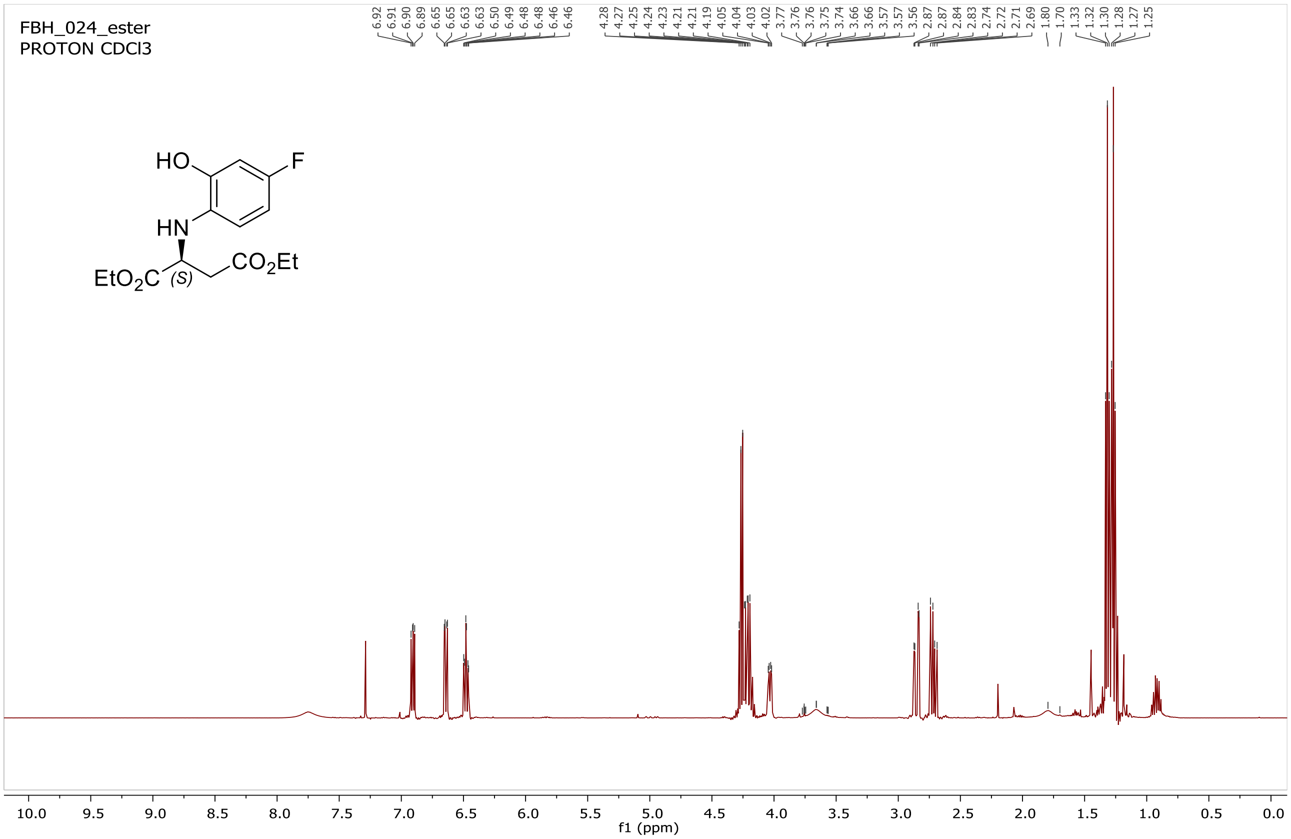


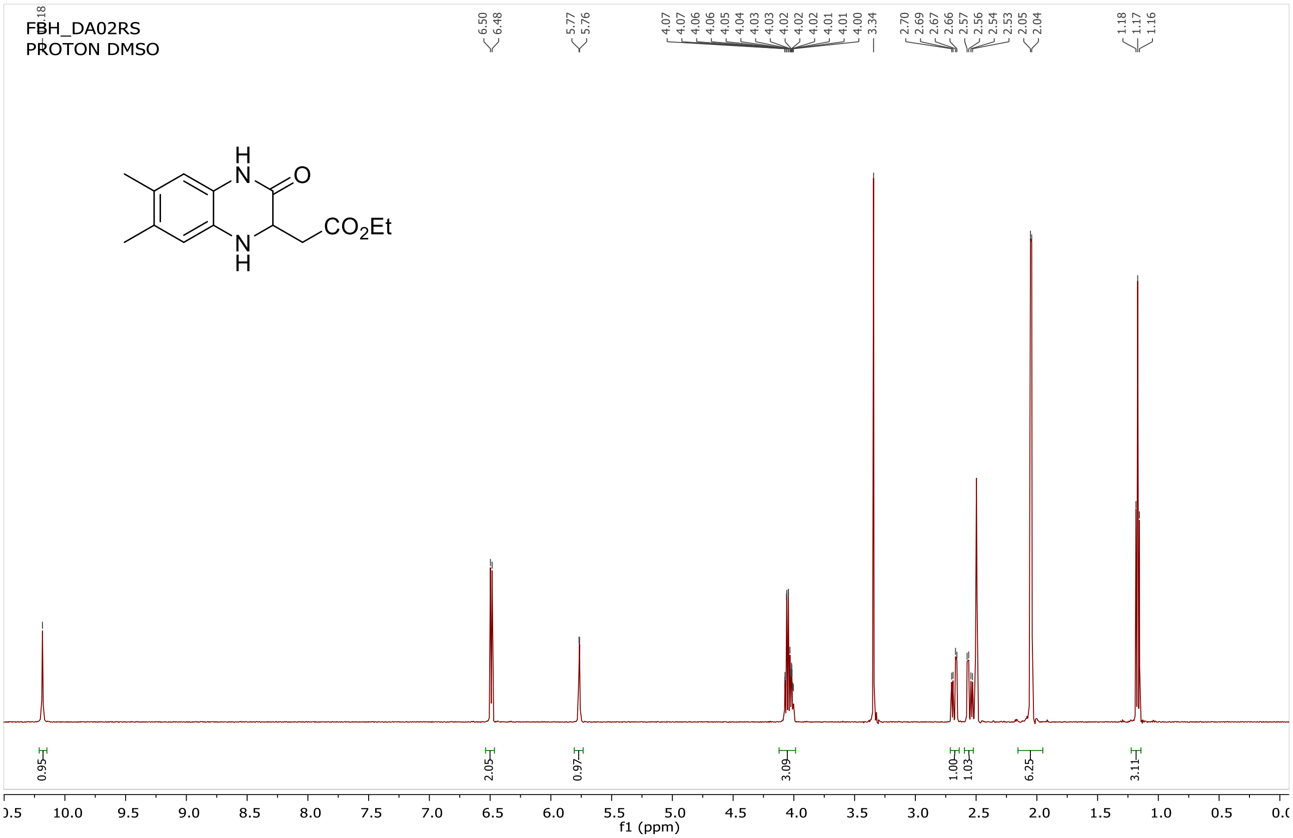


**Figure S32:** $^{1}$H NMR (top) of diethyl ester intermediate of (**4d**) and $^{1}$H NMR (bottom) of intermediate (**10**).

## IV) HPLC DATA:

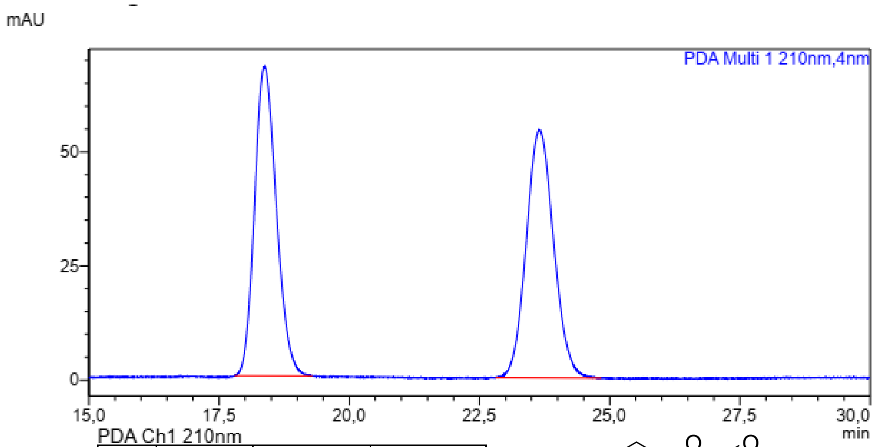


PDA Ch1 210nm

| Peak# | Ret. Time | Area | Area% |
|---|---|---|---|
| 1 | 18,367 | 1976196 | 49,778 |
| 2 | 23,650 | 1993797 | 50,222 |
| Total | | 3969993 | 100,000 |

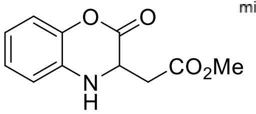


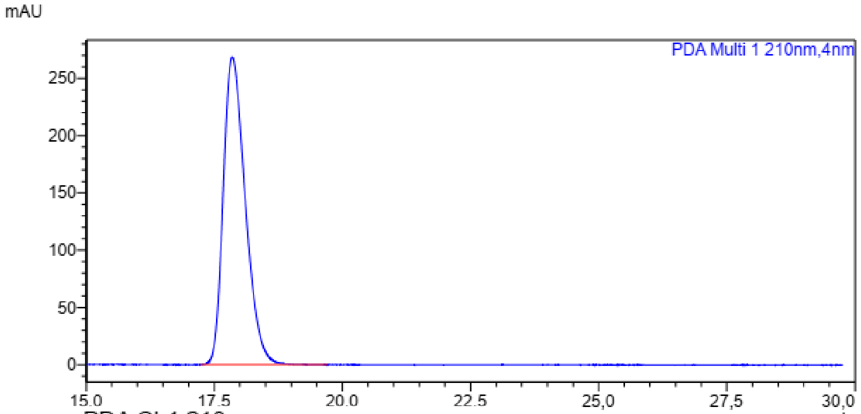


PDA Ch1 210nm

| Peak# | Ret. Time | Area | Area% |
|---|---|---|---|
| 1 | 17,851 | 8011268 | 100,000 |
| Total | | 8011268 | 100,000 |

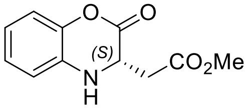


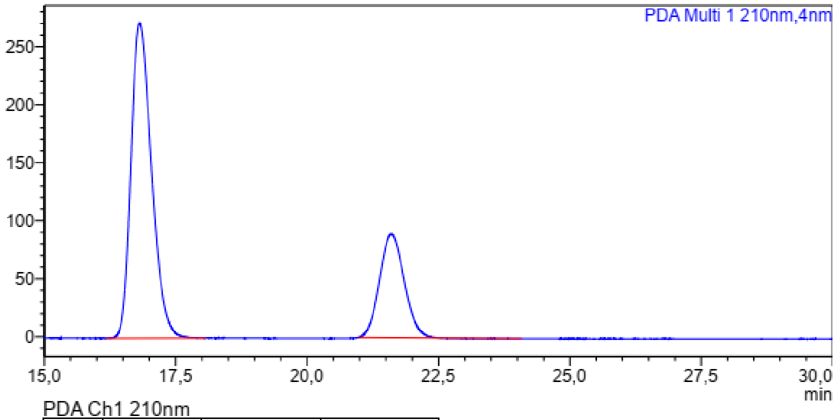


PDA Ch1 210nm

| Peak# | Ret. Time | Area | Area% |
|---|---|---|---|
| 1 | 16,813 | 7406730 | 71,822 |
| 2 | 21,603 | 2905849 | 28,178 |
| Total | | 10312578 | 100,000 |

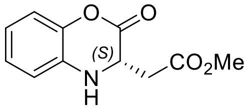


**Figure S33.**Chiral HPLC analysis ofracemic **7aa**(top), enzymatic product**4aa**(middle),and spiking (bottom)sample with enzymatic product and racemic reference showing enrichment of the (*S*)-enantiomer.

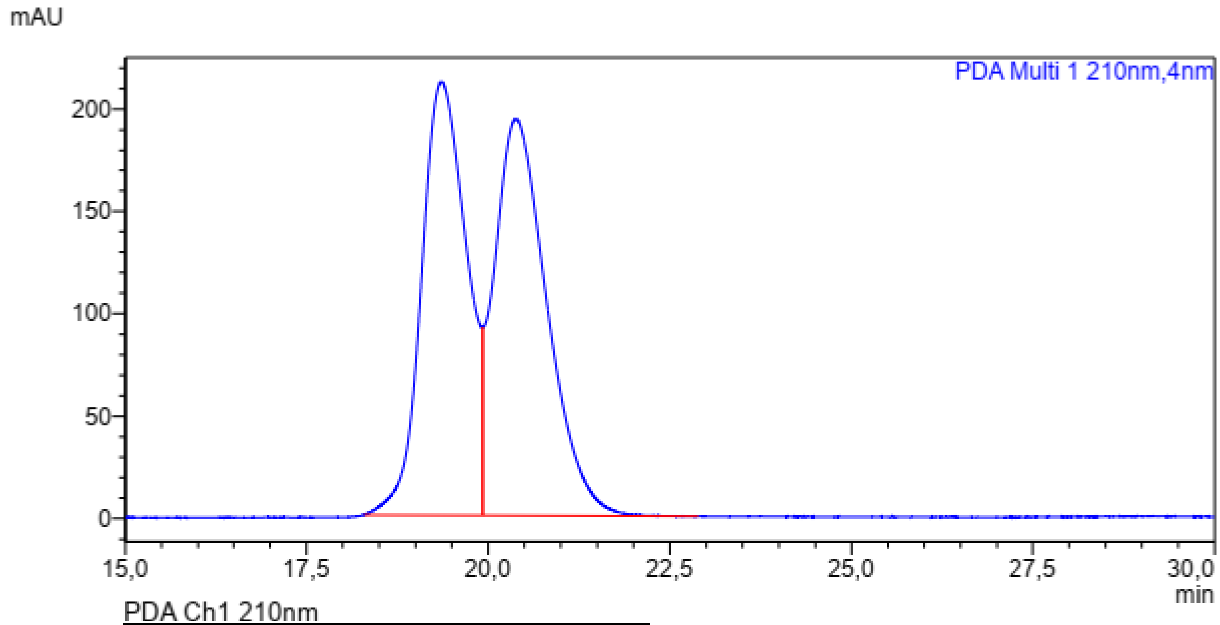


PDA Ch1 210nm

| Peak# | Ret. Time | Area | Area% |
|---|---|---|---|
| 1 | 19,357 | 9205032 | 48,476 |
| 2 | 20,381 | 9783682 | 51,524 |
| Total | | 18988714 | 100,000 |

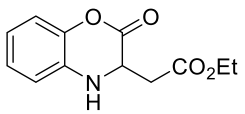


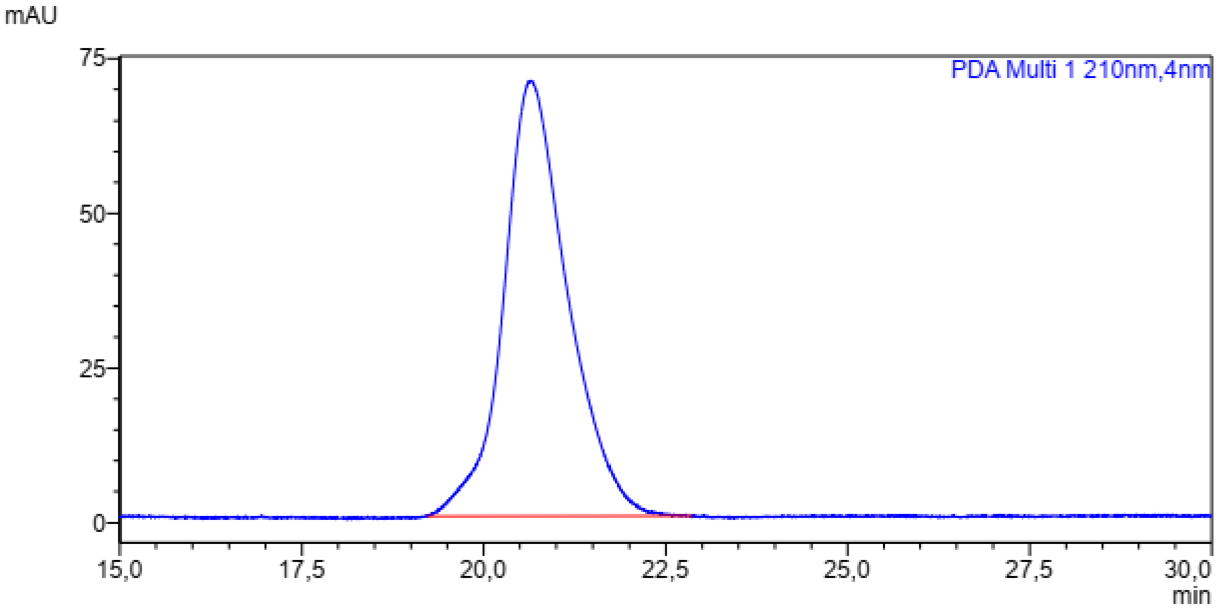


PDA Ch1 210nm

| Peak# | Ret. Time | Area | Area% |
|---|---|---|---|
| 1 | 20,645 | 4309647 | 100,000 |
| Total | | 4309647 | 100,000 |

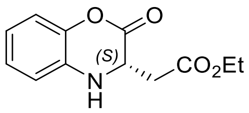


**Figure S34.** Chiral HPLC analysis of racemic **7a**(top) and enzymatic product **4a**(bottom) using OD-H column.

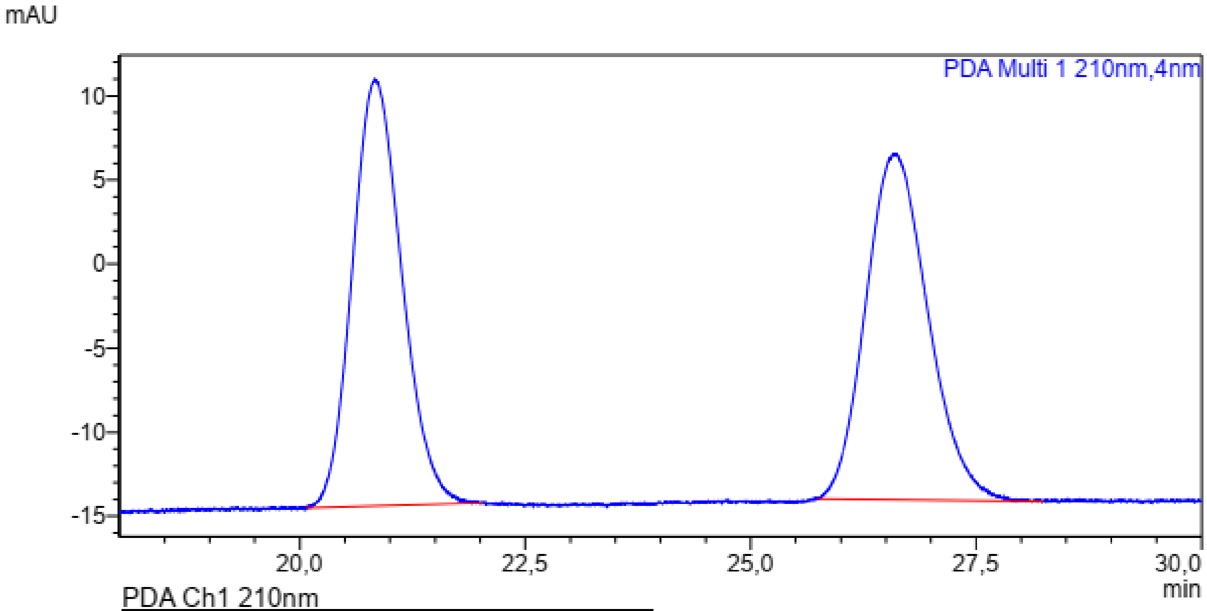


PDA Ch1 210nm

| Peak# | Ret. Time | Area | Area% |
|---|---|---|---|
| 1 | 20,839 | 971318 | 49,550 |
| 2 | 26,607 | 988943 | 50,450 |
| Total | | 1960262 | 100,000 |

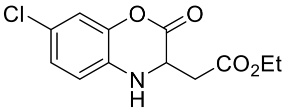


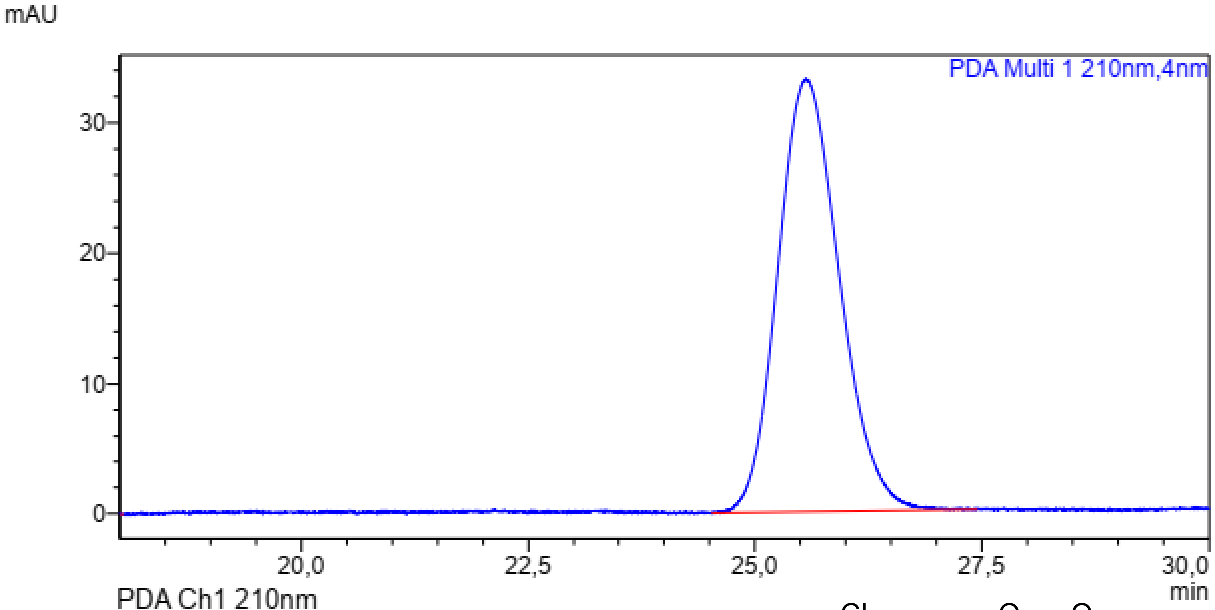


PDA Ch1 210nm

| Peak# | Ret. Time | Area | Area% |
|---|---|---|---|
| 1 | 25,559 | 1570343 | 100,000 |
| Total | | 1570343 | 100,000 |

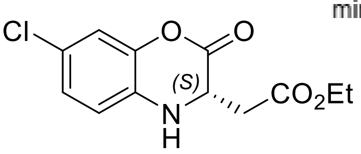


**Figure S35.**Chiral HPLC analysis of racemic **7b**(top) and enzymatic product **4b**(bottom) using OD-H column.

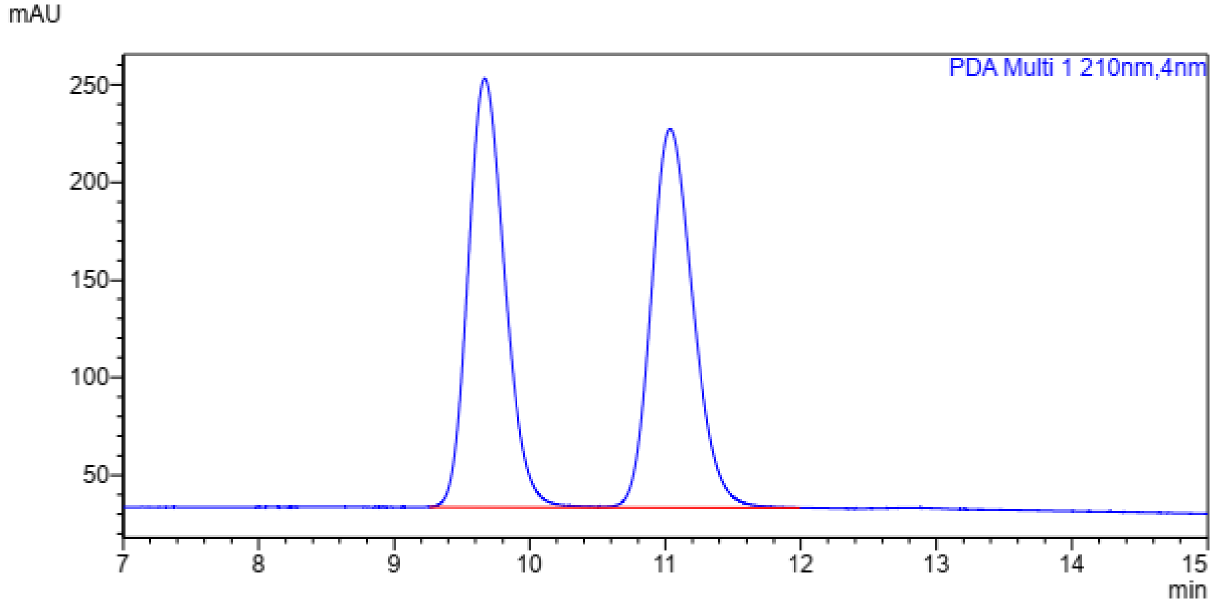


PDA Ch1 210nm

| Peak# | Ret. Time | Area | Area% |
|---|---|---|---|
| 1 | 9,667 | 4114597 | 49,788 |
| 2 | 11,035 | 4149574 | 50,212 |
| Total | | 8264170 | 100,000 |

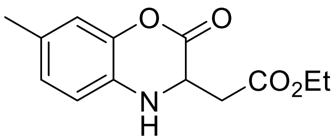


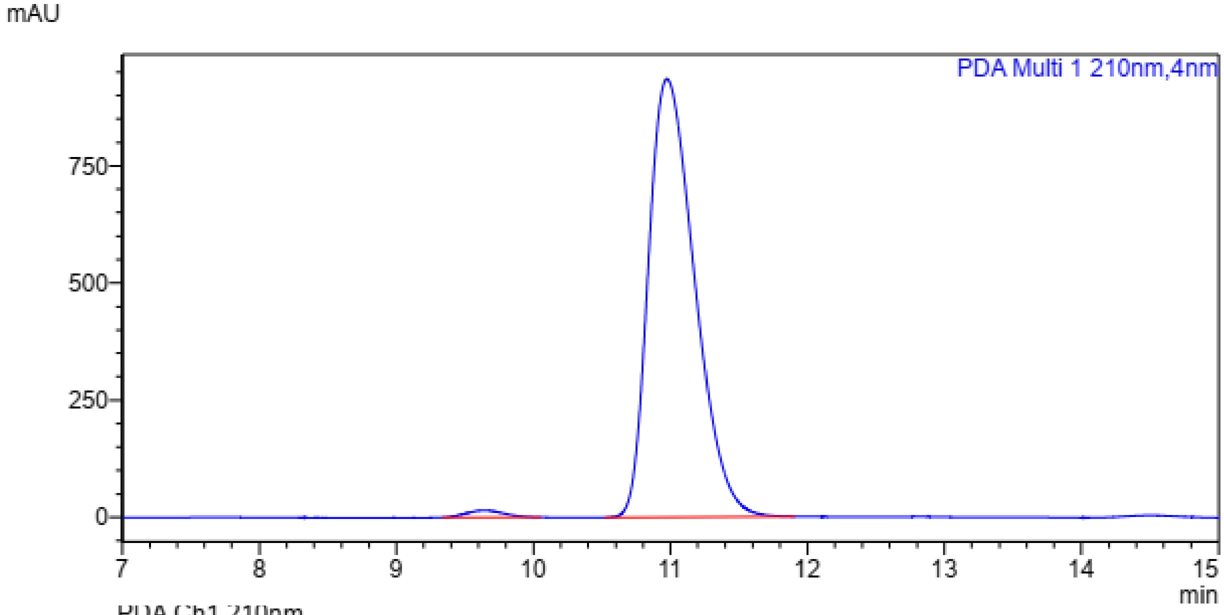


PDA Ch1 210nm

| Peak# | Ret. Time | Area | Area% |
|---|---|---|---|
| 1 | 9,641 | 287096 | 1,291 |
| 2 | 10,975 | 21953470 | 98,709 |
| Total | | 22240566 | 100,000 |

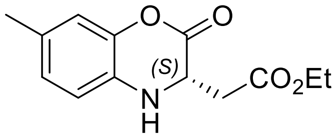


**Figure S36.**Chiral HPLC analysis of racemic**7c**(top) and enzymaticproduct **4c**(bottom)using OD-H column.

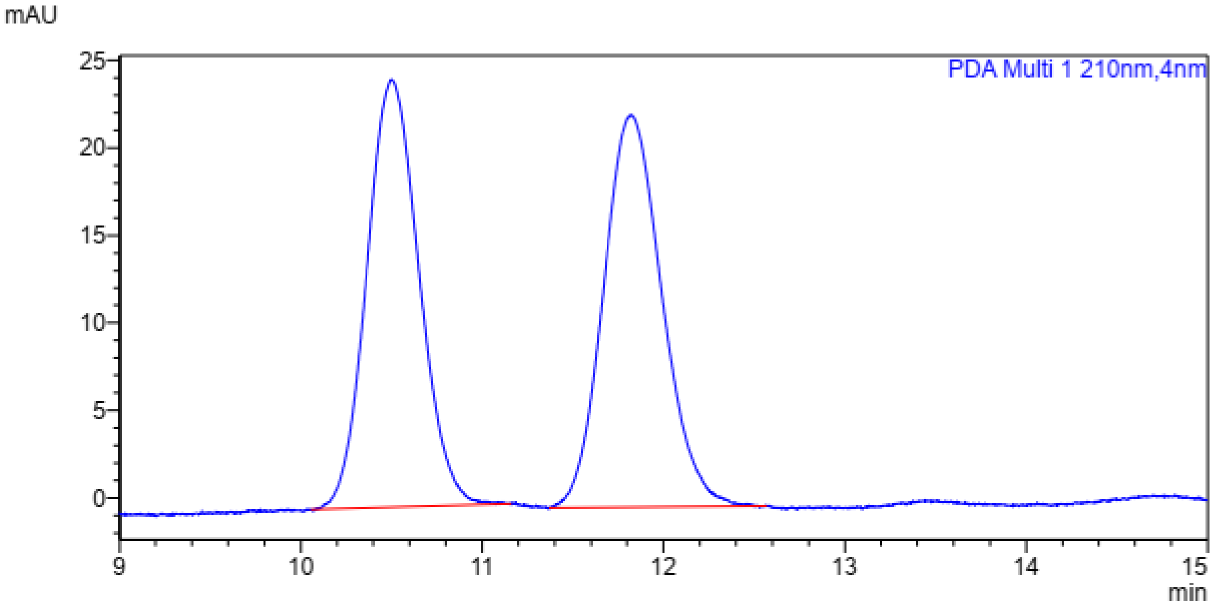


PDA Ch1 210nm

| Peak# | Ret. Time | Area | Area% |
|---|---|---|---|
| 1 | 10,502 | 489607 | 49,257 |
| 2 | 11,822 | 504378 | 50,743 |
| Total | | 993985 | 100,000 |

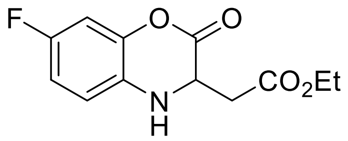


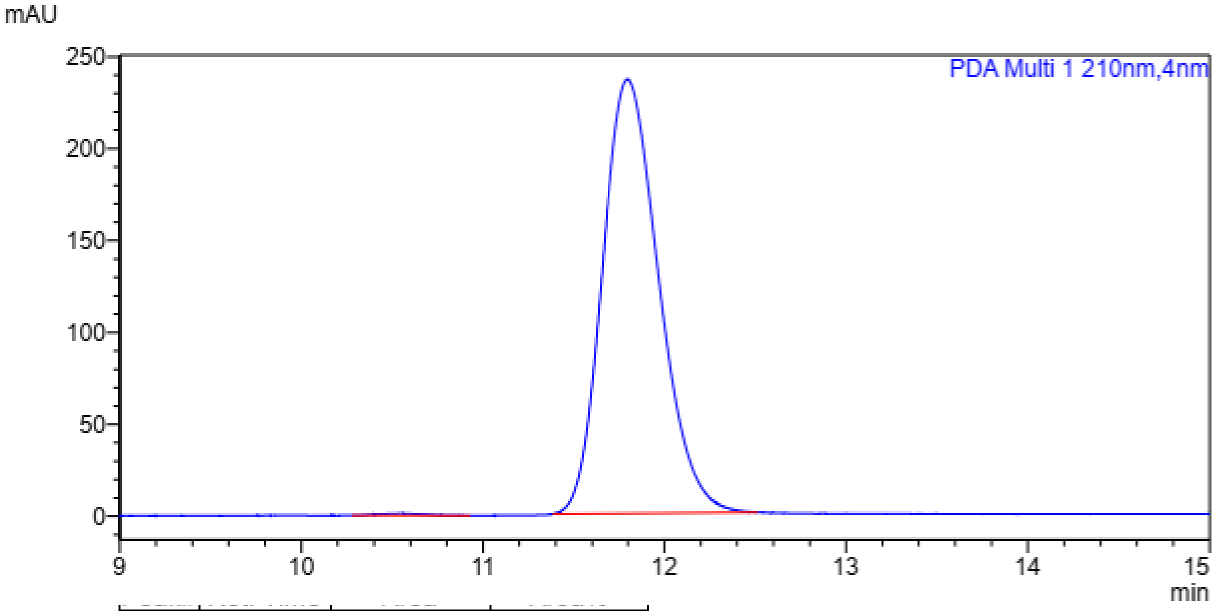


| | | | |
|---|---|---|---|
| 1 | 10,550 | 18663 | 0,363 |
| 2 | 11,796 | 5116459 | 99,637 |
| Total | | 5135122 | 100,000 |

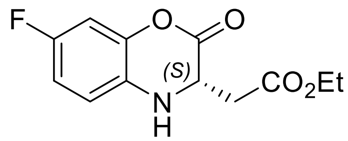


**Figure S37.**Chiral HPLC analysis of racemic **7d**(top) and enzymatic product **4d**(bottom)using OD-H column.

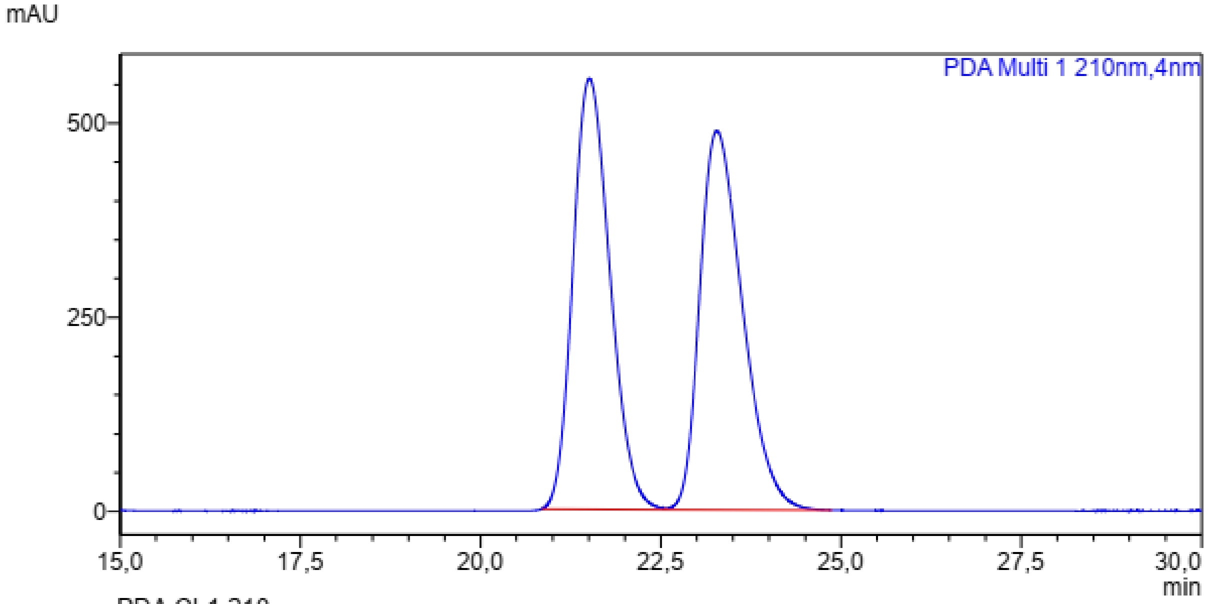


PDA Ch1 210nm

| Peak# | Ret. Time | Area | Area% |
|---|---|---|---|
| 1 | 21,510 | 19844951 | 49,744 |
| 2 | 23,275 | 20048883 | 50,256 |
| Total | | 39893834 | 100,000 |

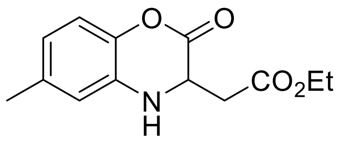


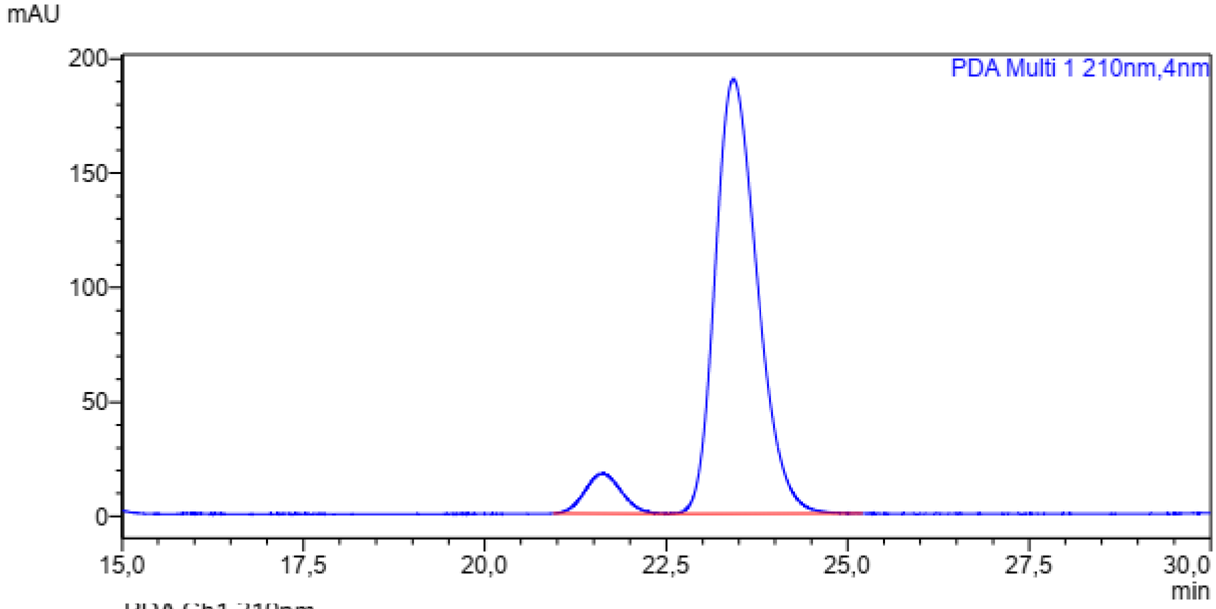


PDA Ch1 210nm

| Peak# | Ret. Time | Area | Area% |
|---|---|---|---|
| 1 | 21,617 | 610630 | 7,365 |
| 2 | 23,422 | 7680000 | 92,635 |
| Total | | 8290630 | 100,000 |

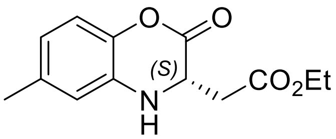


**Figure S38.**Chiral HPLC analysis of racemic **7f**(top) and enzymatic product **4f** (bottom)using OD-H column.

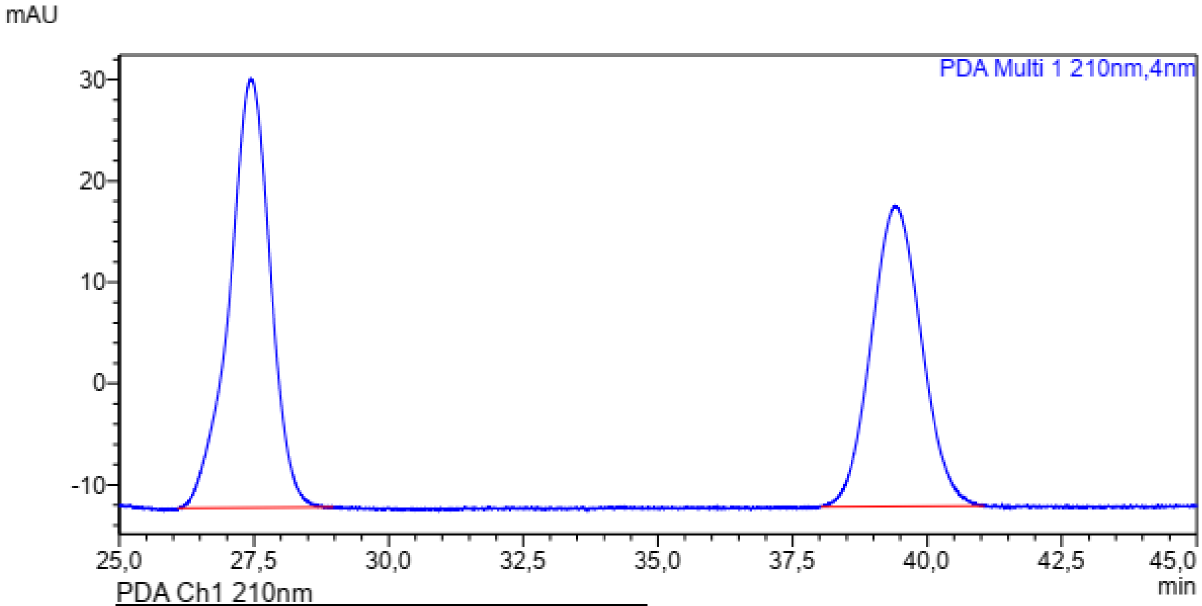


PDA Ch1 210nm

| Peak# | Ret. Time | Area | Area% |
|---|---|---|---|
| 1 | 27,446 | 2238349 | 53,745 |
| 2 | 39,411 | 1926429 | 46,255 |
| Total | | 4164778 | 100,000 |

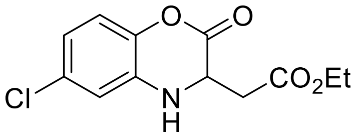


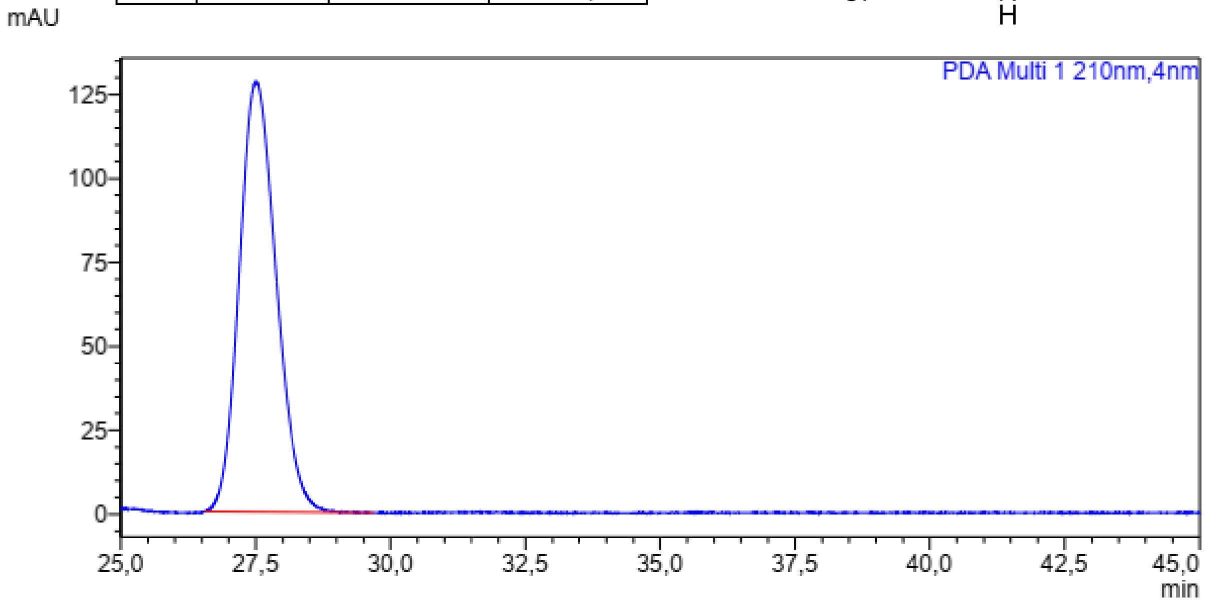


PDA Ch1 210nm

| Peak# | Ret. Time | Area | Area% |
|---|---|---|---|
| 1 | 27,503 | 6092922 | 100,000 |
| Total | | 6092922 | 100,000 |

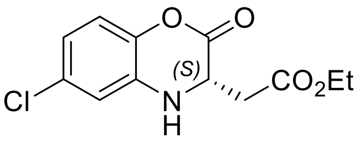


**Figure S39.**Chiral HPLC analysis of racemic**7g**(top) and enzymatic product **4g**(bottom)using AD-H column.

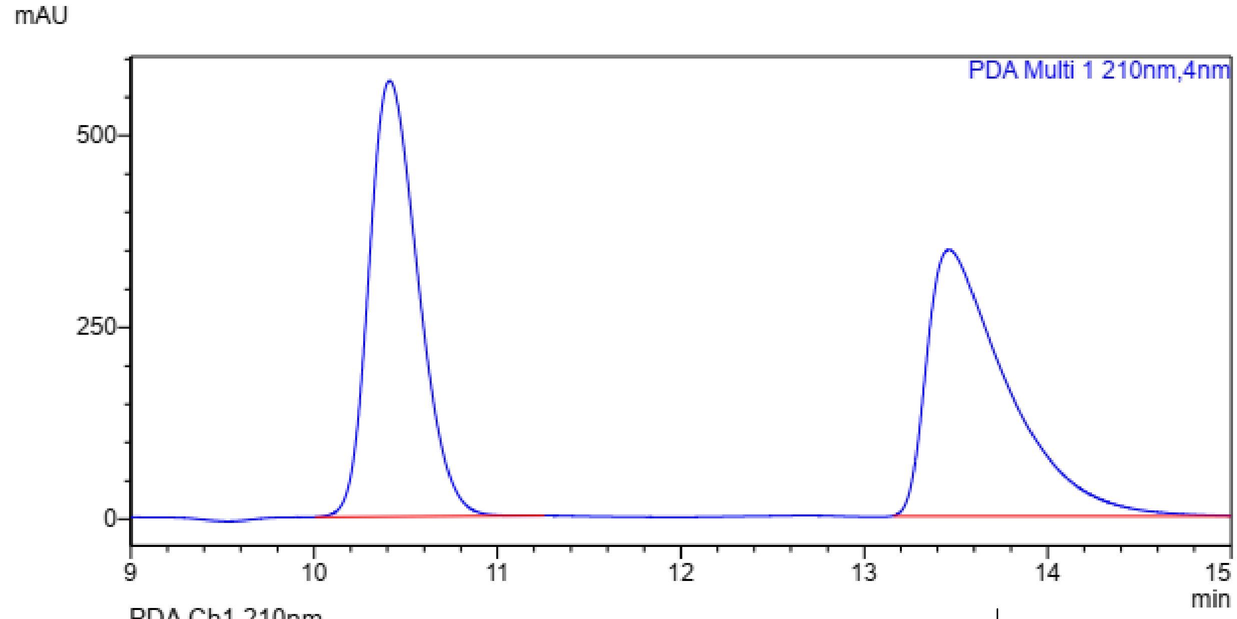


PDA Ch1 210nm

| Peak# | Ret. Time | Area | Area% |
|---|---|---|---|
| 1 | 10,412 | 10576890 | 49,945 |
| 2 | 13,462 | 10600235 | 50,055 |
| Total | | 21177125 | 100,000 |

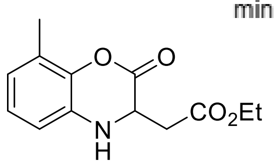


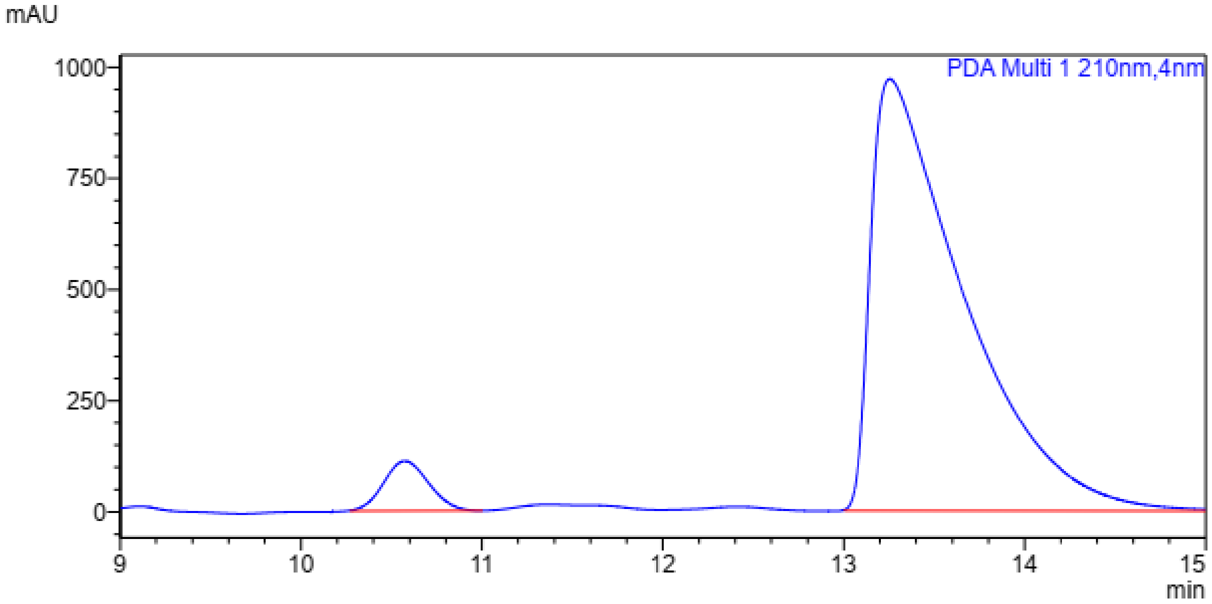


PDA Ch1 210nm

| Peak# | Ret. Time | Area | Area% |
|---|---|---|---|
| 1 | 10,573 | 1921725 | 5,241 |
| 2 | 13,260 | 34746333 | 94,759 |
| Total | | 36668058 | 100,000 |

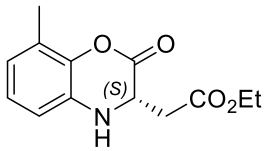


**Figure S40.** Chiral HPLC analysis of racemic **7h** (top) and enzymatic product **4h** (bottom) using ID column.

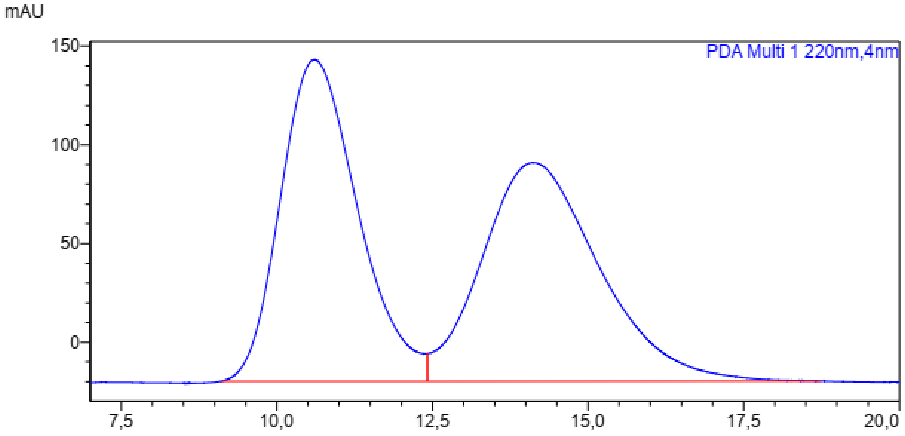


PDA Ch1 220nm

| Peak# | Ret. Time | Area | Area% |
|---|---|---|---|
| 3 | 10,603 | 14088896 | 49,349 |
| 4 | 14,120 | 14460627 | 50,651 |
| Total | | 28549523 | 100,000 |

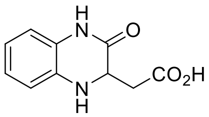


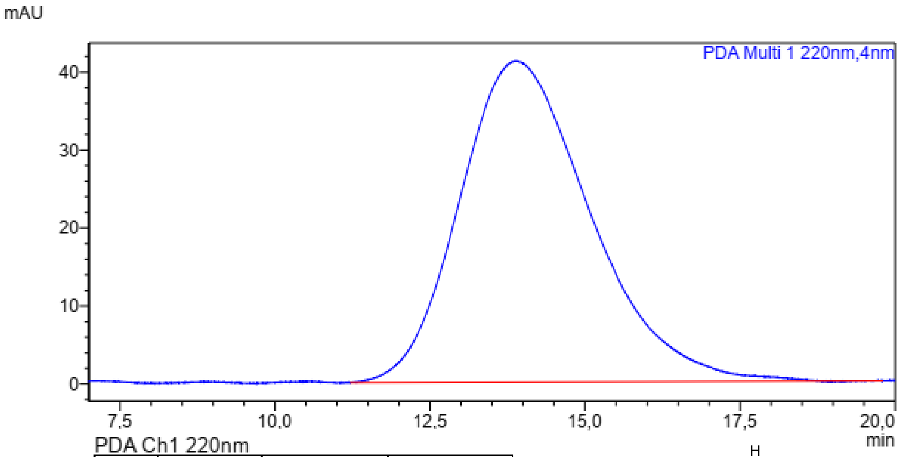


PDA Ch1 220nm

| Peak# | Ret. Time | Area | Area% |
|---|---|---|---|
| 3 | 13,875 | 6028018 | 100,000 |
| Total | | 6028018 | 100,000 |

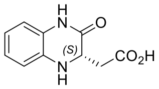


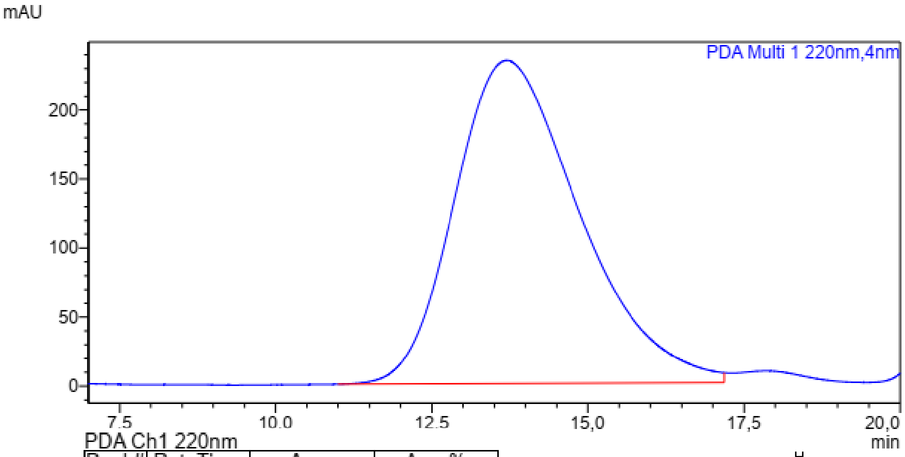


PDA Ch1 220nm

| Peak# | Ret. Time | Area | Area% |
|---|---|---|---|
| 3 | 13,700 | 32600092 | 100,000 |
| Total | | 32600092 | 100,000 |

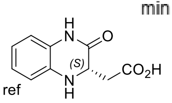


ref

**Figure S41.**Chiral HPLC analysis of racemic**9** (top), enzymatic**5p**(middle), and authentic (*S*) reference(bottom) product usingAD-RH column.

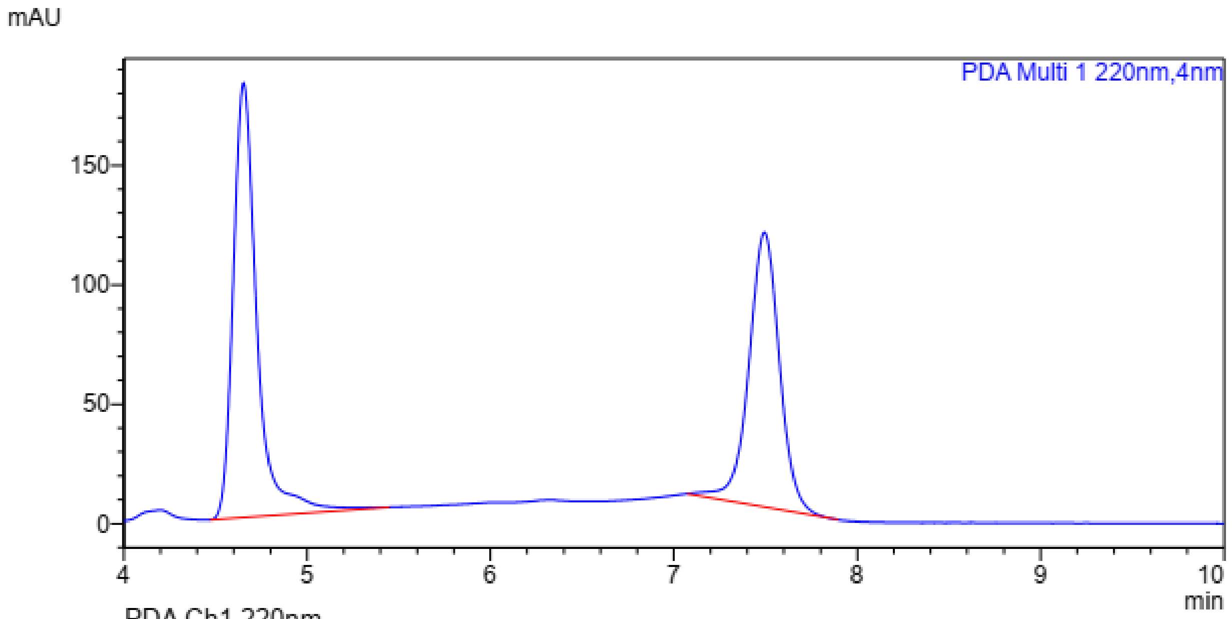


PDA Ch1 220nm

| Peak# | Ret. Time | Area | Area% |
|---|---|---|---|
| 1 | 4,652 | 1646303 | 54,980 |
| 2 | 7,493 | 1348043 | 45,020 |
| Total | | 2994347 | 100,000 |

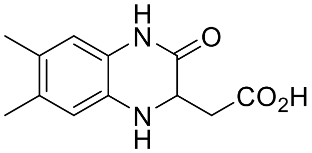


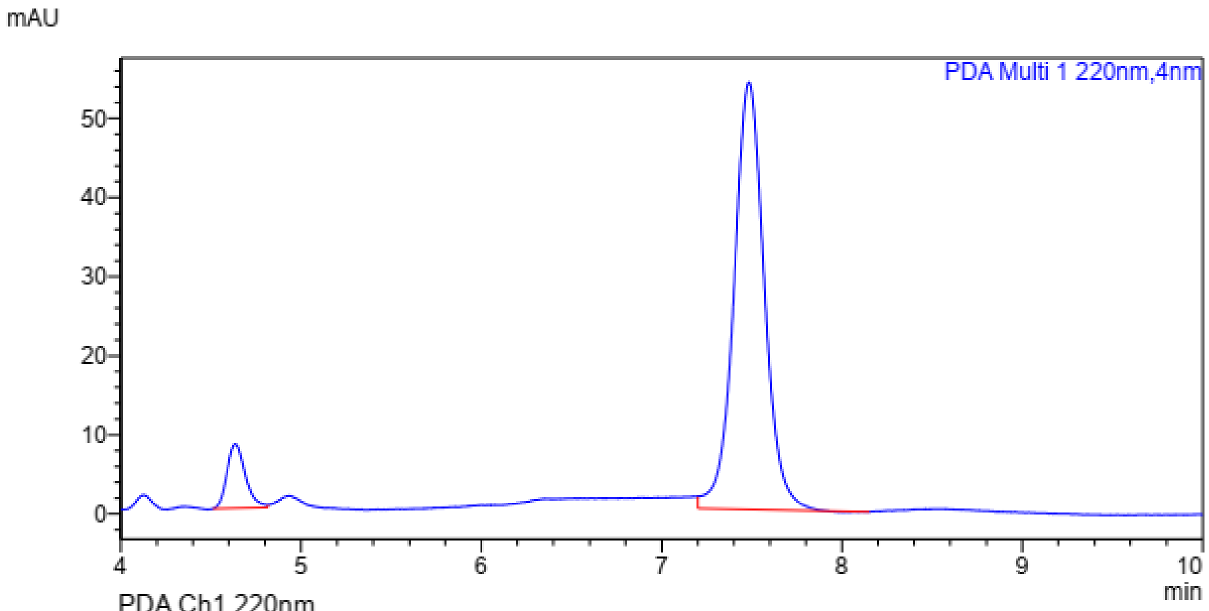


PDA Ch1 220nm

| Peak# | Ret. Time | Area | Area% |
|---|---|---|---|
| 1 | 4,635 | 58504 | 8,439 |
| 2 | 7,484 | 634764 | 91,561 |
| Total | | 693268 | 100,000 |

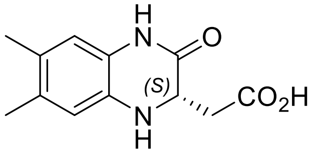


**Figure S42.**Chiral HPLC analysis of racemic**11** (top) and enzymatic **5q**(bottom) product using OD-RH column

**References:**


(1) Fu, H.; Prats Luján, A.; Bothof, L.; Zhang, J.; Tepper, P. G.; Poelarends, G. J. Biocatalytic Asymmetric Synthesis of N-Aryl-Functionalized Amino Acids and Substituted Pyrazolidinones. *ACS Catal.*2019, *9* (8), 7292–7299.

(2) Chen, Z.; Yin, X.; Dong, X.-Q.; Zhang, X. Efficient Access to Chiral Dihydrobenzoxazinones via Rh-Catalyzed Hydrogenation. *RSC Adv.*2019, *9* (27), 15466–15469.

(3) Santos-Sanchez, N. F.; Salas-Coronado, R.; Colorado-Peralta, R.; Pena-Hueso, A.; Sanchez-Ruiz, S. A.; Flores-Parra, A. Novel Synthesis of 1-Alkyl-4-Tosyl-3-Carboxymethyl-1, 2, 3, 4-Tetrahydroquinoxalin-2-Ones. *Arkivoc*2008, 187–199.

(4) Ismail, M. M. F.; Ammar, Y. A.; Ibrahim, M. K.; El-Zahaby, H. S. A.; Mahmoud, S. S. Synthesis and Pharmacological Evaluation of Novel Quinoxalines as Potential Nonulcerogenic Anti-Inflammatory and Analgesic Agents. *Arzneimittelforschung*2005, *55* (12), 738–743.

(5) Li, D.; Ollevier, T. Iron-or Zinc-Mediated Synthetic Approach to Enantiopure Dihydroquinoxalinones. *European J. Org. Chem.*2019, *2019* (6), 1273–1280.
